# Vegetarian and vegan diets and cancer incidence: a systematic review and meta-analysis of prospective studies

**DOI:** 10.1007/s10654-026-01380-8

**Published:** 2026-03-25

**Authors:** Dagfinn Aune, Sabrina Schlesinger, Jakub G. Sobiecki

**Affiliations:** 1 Department of Epidemiology and Biostatistics, School of Public Health, Imperial College London, White City Campus, 90 Wood Lane, London, W12 0BZ UK; 2 Department of Research, Cancer Registry of Norway, Norwegian Institute of Public Health, Oslo, Norway; 3 Department of Nutrition, Oslo New University College, Oslo, Norway; 4 Institute for Biometrics and Epidemiology, German Diabetes Center, Leibniz Center for Diabetes Research at Heinrich Heine University Düsseldorf, 40225 Düsseldorf, Germany; 5 German Center for Diabetes Research (DZD), Partner Düsseldorf, München-Neuherberg, 40225 Düsseldorf, Germany

**Keywords:** Vegetarian, Vegan, Cancer, Prospective study, Meta-analysis

## Abstract

Several studies have suggested that vegetarian and vegan vs. non-vegetarian diets are associated with lower cancer risk overall, however, results for specific cancer sites have been less consistent. We conducted a systematic review and meta-analysis of prospective studies on vegetarian and vegan diets and cancer incidence to clarify the associations across cancer sites. PubMed and Embase databases were searched for relevant studies up to 5 July 2025. Summary relative risks (RRs) and 95% confidence intervals (95% CIs) were calculated for the association between vegetarian and vegan diets and cancer incidence. World Cancer Research Fund (WCRF) criteria was used to evaluate the strength of the evidence. Seventeen publications (seven prospective studies) were included. The summary RRs (95% CIs) for vegetarians vs. non-vegetarians was 0.87 (0.84–0.91, I^2^ = 0%, *n* = 4 studies) for total cancer incidence, 0.55 (0.36–0.86, I^2^ = 32%, *n* = 4) for stomach cancer, 0.86 (0.76–0.97, I^2^ = 14%, *n* = 6) for colorectal cancer, 0.79 (0.67–0.93, I^2^ = 38%, *n* = 7) for colon cancer, 0.55 (0.31–0.97, I^2^ = 0%, *n* = 2) for proximal colon cancer, 0.77 (0.62–0.95, I^2^ = 0%, *n* = 5) for pancreatic cancer, 0.79 (0.66–0.94, I^2^ = 0%, *n* = 4) for melanoma, 0.92 (0.86–0.99, I^2^ = 0%, *n* = 7) for breast cancer, 0.81 (0.69–0.95, I^2^ = 0%, *n* = 3) for postmenopausal breast cancer, 0.78 (0.62–0.98, I^2^ = 0%, *n* = 5) for bladder cancer, and 0.76 (0.63–0.93, I^2^ = 0%, *n* = 4) for non-Hodgkin’s lymphoma. In addition, non-statistically significant inverse associations were observed for some cancers, with summary RRs of 0.85 (0.70–1.04, I^2^ = 0%, *n* = 6) for lung cancer, 0.83 (0.68–1.02, I^2^ = 0%, *n* = 5) for ovarian cancer, and 0.87 (0.75-1.00, I^2^ = 43%, *n* = 6) for prostate cancer. Results for other cancer sites were imprecise or near the null. The summary RRs (95% CIs) for vegans vs. non-vegetarians were 0.77 (0.70–0.85, I^2^ = 0%, *n* = 3) for total cancer, 1.02 (0.71–1.48, I^2^ = 42%, *n* = 3) for colorectal cancer, 0.80 (0.64–1.00, I^2^ = 0%, *n* = 4) for breast cancer, and 0.87 (0.50–1.49, I^2^ = 49%, *n* = 3) for prostate cancer. BMI explained a moderate part of the associations. The strength of evidence [judging the likelihood of causality] for vegetarian diets and total, colorectal, colon and breast cancer was judged as probable, and limited suggestive for stomach, pancreatic, and bladder cancers, melanoma and non-Hodgkin’s lymphoma, and for vegan diets and total cancer and breast cancer was considered limited-suggestive. Vegetarian diets compared to non-vegetarian diets are associated with reduced risk of total cancer and seven specific cancer types, while vegan diets are associated with reduced risk of total and breast cancer. Although further studies are needed to assess the long-term adherence to vegetarian and vegan diets and cancer incidence and across less investigated cancers, these results support recommendations to adopt much more plant-based diets for cancer prevention.

**Supplementary Information:**

The online version contains supplementary material available at 10.1007/s10654-026-01380-8.

## Introduction

Cancer is a major cause of morbidity and the second leading cause of death globally with 20 million new cases and 9.7 million deaths occurring in 2022 [1]. The observations of large international variation in cancer rates [1], secular trends in cancer rates over time within countries with Westernization of lifestyles [2], and changing cancer rates in individuals migrating from countries with a low incidence to countries with a high incidence of certain cancers (e.g. colorectal, breast, prostate cancers) [3], provides strong evidence that modifiable risk factors are important in cancer etiology. Smoking [4], alcohol consumption [5], adiposity [5] and low physical activity [5, 6] are established risk factors for a wide range of cancers. Dietary factors also are of major importance for some individual cancers, but less is known with certainty across different cancer types [5].

A growing body of evidence suggests that plant-based diets, high in fruits, vegetables, whole grains, nuts, and legumes and with low or no intake of red and processed meat, is associated with reduced cancer risk [5, 7–9]. Consistent with this, some studies have suggested vegetarian diets are associated with reduced incidence of digestive tract, colorectal, breast and prostate cancer, however, not all studies have been consistent. In the Adventist Health Study, vegetarians had nearly half the risk of colon cancer compared to meat eaters [10, 11], and in the Adventist Health Study 2 vegetarians had a 21% lower risk of colorectal cancer compared to meat eaters [12]. Other studies reported non-statistically significant inverse associations between vegetarian diets and colorectal cancer [13, 14]; however, in a combined analysis of the EPIC-Oxford Study and the Oxford Vegetarian Study, no association was observed [15]. Studies on vegetarian diets and breast cancer incidence have reported non-statistically significant inverse associations [14, 16–18] or associations close to unity [12, 15]. Studies have also suggested potential inverse associations with stomach [12, 15, 19], lung [10, 12, 15, 18, 19], prostate [12, 14, 15, 18, 20], and bladder [12, 15, 19] cancers, non-Hodgkin’s lymphoma or lymphoma [12, 15, 19] and multiple myeloma [12, 15, 19], however, the associations have not always been consistent across studies or statistically significant.

Studies on vegan diets have reported inverse associations with total cancer [12, 15] and breast cancer [12, 15, 16], while results for colorectal [12, 15], and prostate cancer [12, 15, 21] have been more mixed. Although the previous conclusions from the Third Expert Report of the World Cancer Research Fund (WCRF) on vegetarian diets and cancer risk have been inconclusive [5], those conclusions were hampered by a limited number of studies published at the time and are now likely outdated as several additional cohort studies have since been published across cancer sites [12–15, 18, 19, 22, 23].

We therefore conducted a systematic review and meta-analysis to clarify the associations between vegetarian and vegan diets and cancer incidence to provide an up-to-date summary of the evidence across cancer sites.

## Materials and methods

A protocol was developed for the project as part of a grant application for funding. Although this was not pre-registered before the meta-analysis was conducted, it is available through the Open Science Framework (https://osf.io/c3k54/overview).

### Search strategy

We searched PubMed and Embase databases up to 5 July 2025 for eligible studies. The search terms used are provided in the Supplementary text 1. We followed the PRISMA criteria for reporting of systematic reviews and meta-analyses [24]. The reference lists of the included publications were screened for further potentially relevant studies.

### Eligibility, inclusion criteria, and study selection

The review question was framed using the PECO(S) elements [25]. Studies of healthy individuals in the general population with no restrictions with regard to age, sex, or pregnancy status (P), who adhered to vegetarian (excluding meat, poultry or fish) and vegan (excluding meat, poultry, fish, eggs, and dairy products) diets (E) vs. non-vegetarian (including meat, poultry, fish, eggs, dairy) diets (C) and were followed up for cancer incidence (O) were eligible for inclusion. Prospective cohort studies (S) that reported adjusted relative risk (RR) estimates (including hazard ratios, risk ratios and odds ratios) and 95% confidence intervals (CIs) for the association between vegetarian and vegan vs. non-vegetarian diets and cancer incidence were eligible for inclusion. Retrospective case-control and cross-sectional studies were excluded. When several publications were published from the same cohort on the same diet-cancer association we included the publication with the largest number of cases. However, when one publication only reported on the main cancer outcome and a second publication reported on a subtype of this cancer, both publications were included in the respective analyses, but each study was only included once in each analysis to avoid double-counting. DA did the full screening and DA and JGS screened the initial selection of studies (*n* = 242) in duplicate. Any discrepancies were resolved by discussion. A list of the excluded studies and the exclusion reasons can be found in Supplementary Table 1.

### Data extraction

The following data were extracted from each study: The first author’s last name, publication year, country where the study was conducted, the name of the study, study period and duration of follow-up, sample size, sex, age, number of cases, type of diet and comparison, subgroup, RRs and 95% CIs and variables adjusted for in the analysis. The data extraction was conducted by DA and checked for accuracy by JGS.

### Study quality assessment

Study quality was assessed in duplicate by DA and JGS, using a modified version of the Newcastle Ottawa Scale (NOS) which rates studies according to selection, comparability and outcome assessment [26]. The modifications are described in detail in Supplementary text 2.

### Evidence grading

We used WCRF grading criteria to assess the strength of evidence for the associations between vegetarian and vegan diets and cancer incidence [5, 27]. This grading system assesses evidence from different study types, the number of studies available, heterogeneity, study quality, dose-response relationship (when relevant), biological plausibility, and experimental evidence. The evidence is graded as (1) substantial effect on risk unlikely, (2) limited-no conclusion, (3) limited-suggestive, (4) probable, and (5) convincing evidence of a causal relationship using these criteria [5, 27]. The evidence grading was done by discussion between all authors.

### Statistical methods

We used DerSimonian and Laird random effects models to calculate summary RRs (95% CIs) for the association between vegetarian and vegan diets and cancer incidence [28]. The average of the natural logarithm of the RRs was estimated and the RR from each study was weighted using random effects weights [28]. For three articles from the Adventist Health Study that reported on total meat and fish consumption, but not vegetarian status, and risk of colon [11], ovarian [29] and bladder [30] cancer incidence, we pooled categories of meat and fish consumers (vs. non-consumers) using a fixed-effects model and inverted the risk estimates so the comparison became vegetarians vs. non-vegetarians. When estimates were presented without and with BMI adjustment in the same study, estimates without BMI adjustment were prioritized for the main analysis, as there is evidence from randomized trials that vegetarian and vegan diets can help with weight loss [31], and BMI can therefore be considered a mediator. We additionally directly compared analyses without and with BMI adjustment when such results were presented in the same study to quantify how much of any observed associations were due to differences in BMI.

Heterogeneity between studies was evaluated using the Q test and I^2^ statistics [32] and because of a small to moderate number of studies we also present 95% CIs for the I^2^. I^2^ is a measure of how much of the heterogeneity is due to between study variation rather than chance, ranging from 0 to 100%. We conducted main meta-analyses (all studies combined) and stratified by study characteristics such as sex, duration of follow-up (≥ 10 vs. <10 years), geographic location, general population vs. Adventist setting, number of cases, study quality and by adjustment for confounding factors (e.g. age, education, socioeconomic status, smoking, alcohol, physical activity, age at menarche, hormone replacement therapy, oral contraceptive use) to investigate potential sources of heterogeneity when there was at least five studies included in the analysis. These subgroups were chosen as they were considered potential sources of heterogeneity or established risk factors for several cancers, in line with our previous meta-analyses [7, 33]. Publication bias was assessed using Egger’s test [34] and by inspection of funnel plots in analyses with at least five risk estimates. Sensitivity analyses excluding one study at a time were conducted when at least five risk estimates were included in the analysis. Because there was a limited number of studies on vegans we repeated the analyses in vegetarians restricted to the same studies that reported on vegans and compared the result to the overall analysis in vegetarians, to assess whether the studies published on vegan diets were representative for the overall findings. We calculated E-values for the association between vegetarian and vegan diets and cancer incidence, to assess the potential impact of unmeasured or uncontrolled confounding [35]. The statistical analyses were conducted using the software package Stata, version 16.0 (StataCorp, Texas, US).

## Results

**Fig. 1:**
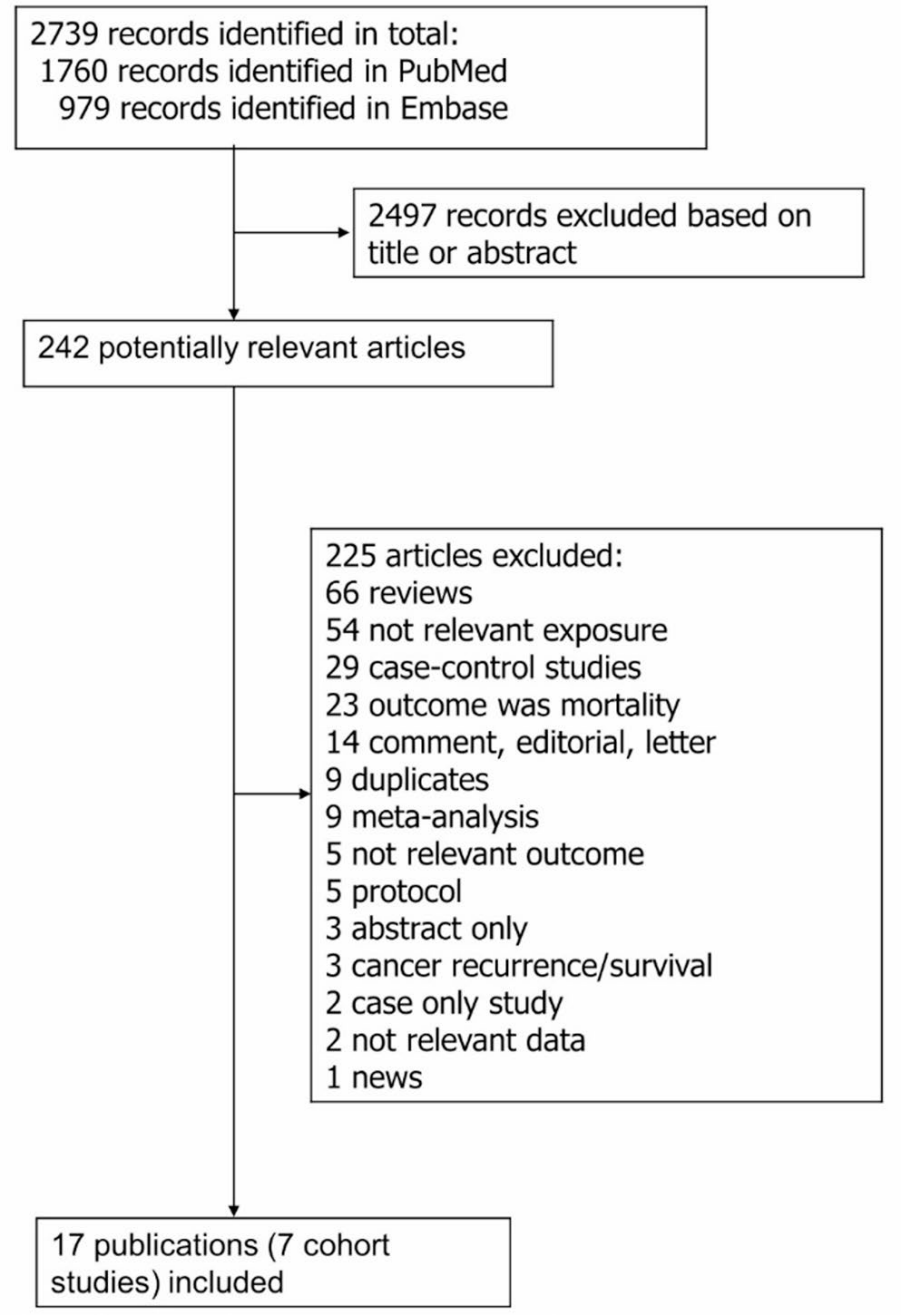
Flow-chart

A total of 2739 records were screened, and of these we identified 17 publications [10–20, 22, 23, 29, 30, 36, 37] covering seven population-based prospective cohort studies that assessed the association between vegetarian or vegan diets and cancer incidence and were included in the analyses (Fig. 1, Supplementary Tables 2–18). The included studies were Oxford Vegetarian Study, European Prospective Investigation into Cancer and Nutrition (EPIC) - Oxford study, UK Women’s Cohort Study, UK Biobank, Netherlands Cohort Study, Adventist Health Study, and Adventist Health Study II. The publication from Oxford Vegetarian Study and EPIC-Oxford Study pooled the two studies together [15], while the remaining studies reported study-specific results. Five studies were from Europe (four from the UK and one from the Netherlands) and two studies were from the USA. All cohorts included both men and women, except for the UK Women’s Cohort Study which only included women. The number of participants ranged from 10,210 to 472,377 and the duration of follow-up ranged from 6 to 20.3 years.

**Fig. 2:**
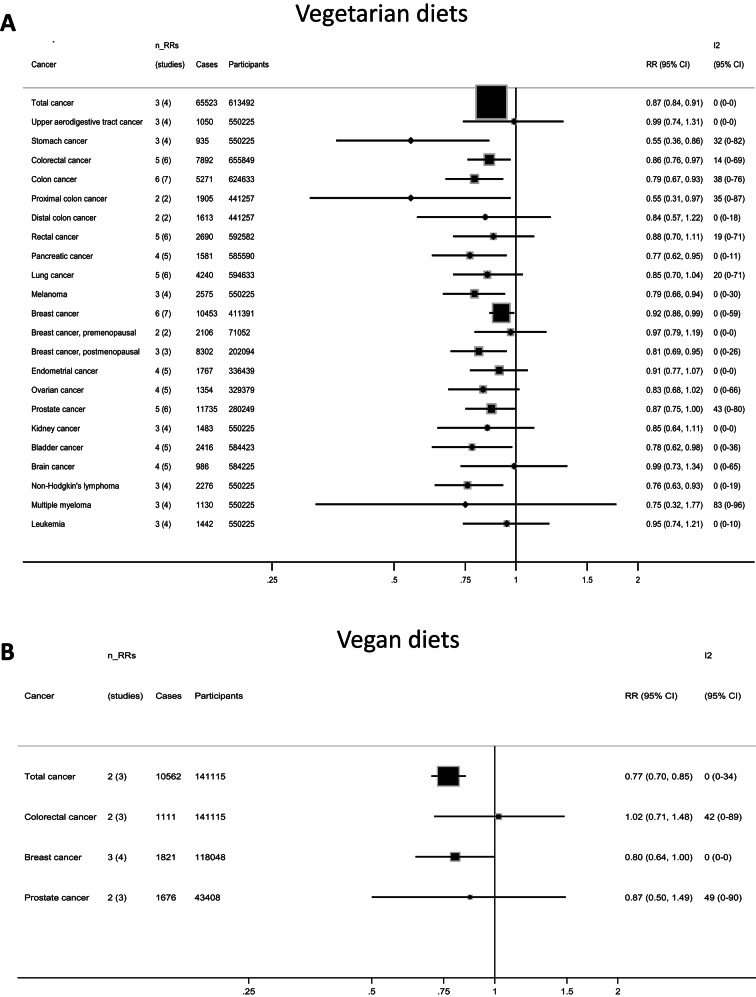
Vegetarian (**a**) and vegan (**b**) diets and cancer incidence, summary estimates across cancers

**Fig. 3:**
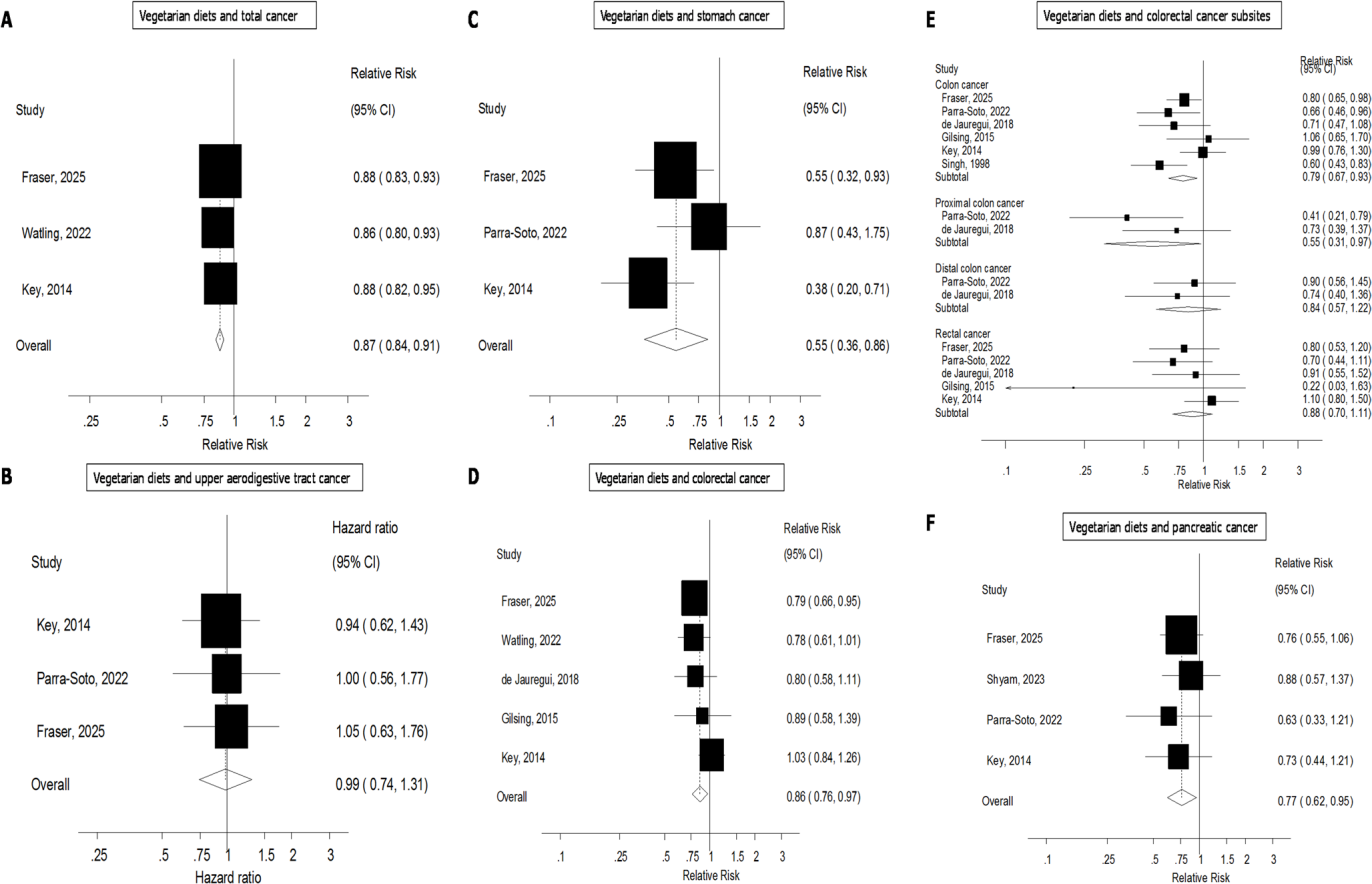
Vegetarian diets and total cancer (**a**), upper aerodigestive cancer (**b**), stomach cancer (**c**), colorectal cancer (**d**), colorectal cancer subsites (**e**), and pancreatic cancer (**f**) incidence

**Fig. 4:**
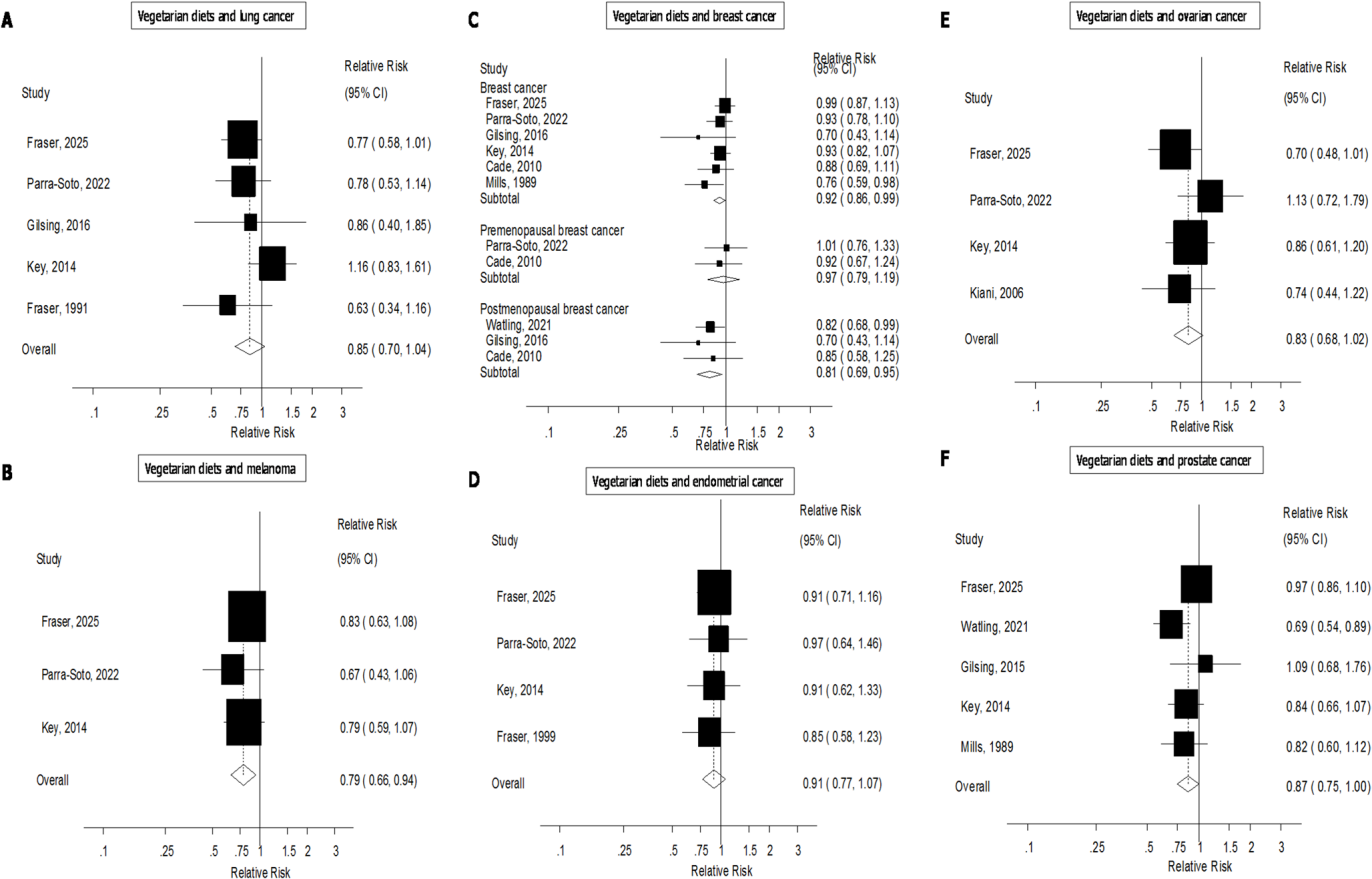
Vegetarian diets and lung cancer (**a**), melanoma (**b**), breast cancer (**c**), endometrial cancer (**d**), ovarian cancer (**e**), and prostate cancer (**f**) incidence

**Fig. 5:**
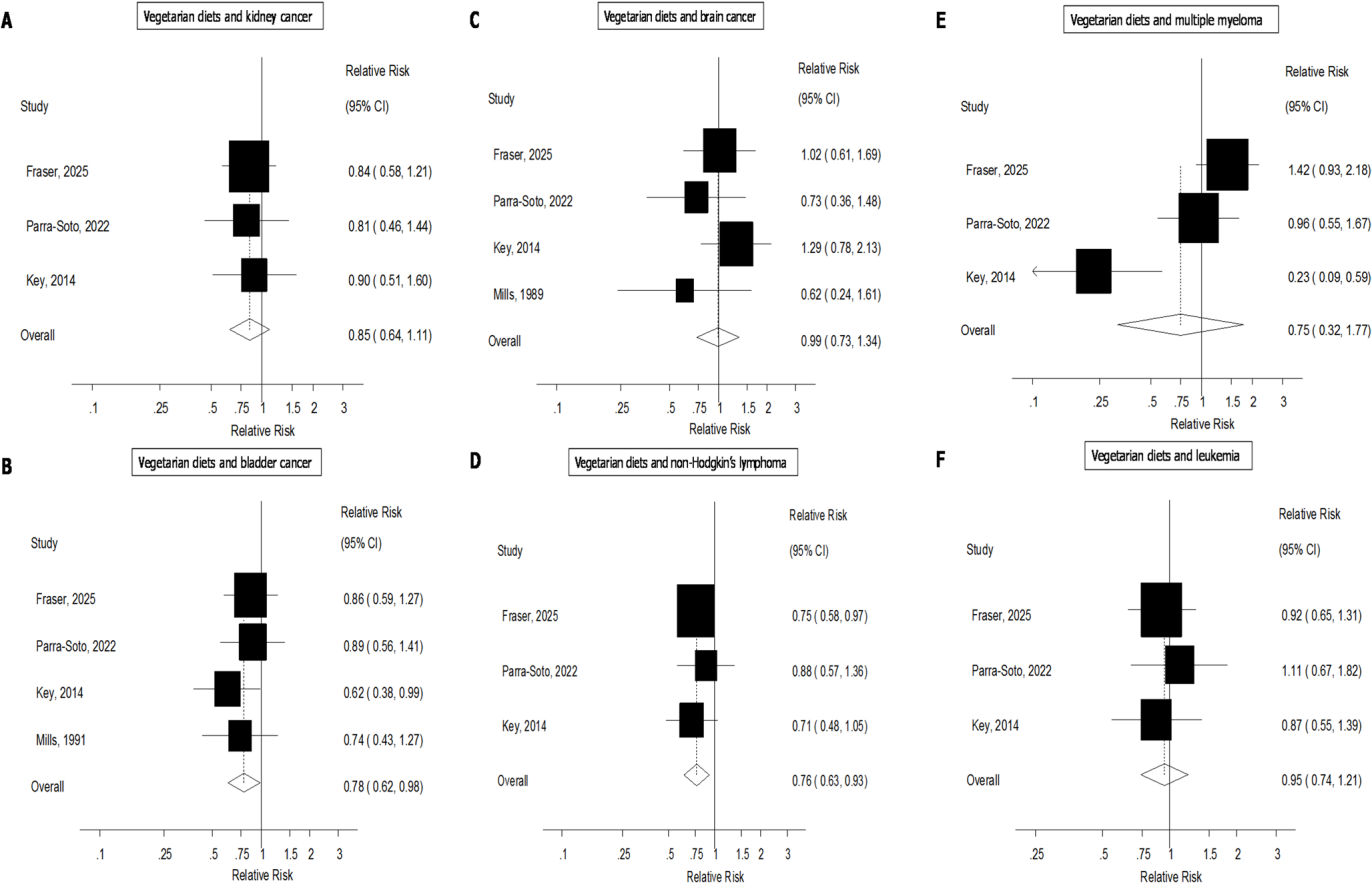
Vegetarian diets and kidney cancer (**a**), bladder cancer (**b**), brain cancer (**c**), non-Hodgkin's lymphoma (**d**), multiple myeloma (**e**) and leukemia (**f**) incidence

**Fig. 6:**
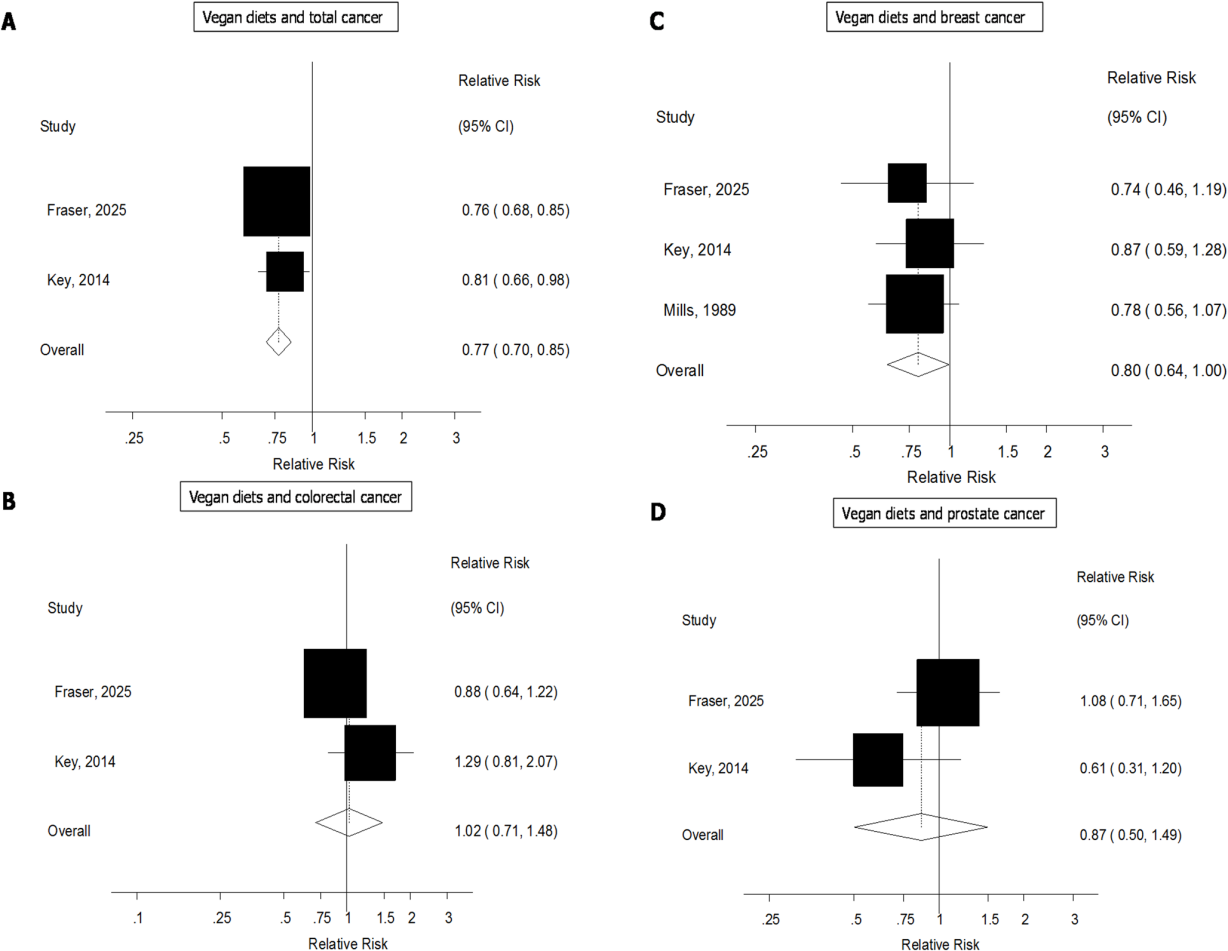
Vegan diets and total cancer (**a**), colorectal cancer (**b**), breast cancer (**c**), and prostate cancer (**d**) incidence

The mean (median) study quality was 7.05 (7.0) out of maximum 8 (Supplementary Table 19). Suboptimal confounder adjustments and lack of reporting on loss to follow-up were the main reasons for suboptimal study quality. Figure 2a and b shows a summary of the results across cancer sites for vegetarians and vegans vs. non-vegetarians, respectively, and the individual forest plots are shown in Figs. 3, 4, 5 and 6.

### Total cancer

Four prospective studies (three risk estimates, three publications, 65,523 cases, 613,492 participants) [12, 14, 15] were included in the analysis of vegetarian diets and total cancer incidence. The summary RR (95% CI) was 0.87 (0.84–0.91, I^2^[95%CI] = 0[0–0]%) for vegetarians vs. non-vegetarians (Figs. 2a and 3a).

Three prospective studies (two risk estimates, two publications, 10,562 cases, 141,115 participants) [12, 15] were included in the analysis of vegan diets and total cancer incidence. The summary RR (95% CI) was 0.77 (0.70–0.85, I^2^ = 0[0–34]%) for vegans vs. non-vegetarians (Figs. 2b and 6c).

### Upper aerodigestive tract cancer

Four prospective studies (three risk estimates, three publications, 1050 cases, 550,225 participants) [12, 15, 19] were included in the analysis of vegetarian diets and upper aerodigestive tract cancer. The summary RR was 0.99 (95% CI: 0.74–1.31, I^2^ = 0[0–0]%) for vegetarians vs. non-vegetarians (Figs. 2a and 3b).

### Stomach cancer

Four prospective studies (three risk estimates, three publications, 935 cases, 550,225 participants) [12, 15, 19] were included in the analysis of vegetarian diets and stomach cancer risk. The summary RR (95% CI) was 0.55 (0.36–0.86, I^2^ = 32[0–82]%) for vegetarians vs. non-vegetarians (Figs. 2a and 3c).

### Colorectal cancer

Six prospective studies (five risk estimates, five publications, 7892 cases, 655,849 participants) [ 12–15, 22] were included in the analysis of vegetarian diets and colorectal cancer incidence. The summary RR for colorectal cancer was 0.86 (0.76–0.97, I^2^ = 14[0–69]%) for vegetarians vs. non-vegetarians (Figs. 2a and 3d). When analysed by subsite, the summary RR was 0.79 (95% CI: 0.67–0.93, I^2^ = 38[0–76]%) for colon cancer [11–15, 22] (5271 cases, 624,633 participants), 0.55 (95% CI: 0.31–0.97, I^2^ = 35[0–87]%) for proximal colon cancer [19, 22] (1905 cases, 441,257 participants), 0.84 (95% CI: 0.57–1.22, I^2^ = 0[0–18]%) for distal colon cancer [19, 22] (1613 cases, 441,257 participants), and 0.88 (95% CI: 0.70–1.11, I^2^ = 19[0–71]%) for rectal cancer [12–15, 22] (2690 cases, 592,582 participants) (Figs. 2a and 3e). There was no indication of publication bias with Egger’s test for colorectal (*p* = 0.45), colon (*p* = 0.85) or rectal cancer (*p* = 0.17) (Supplementary Fig. 1, 2 and 3).

Three cohort studies (two risk estimates, two publications, 1111 cases, 141,115 participants) [12, 15] were included in the analysis of vegan diets and colorectal cancer. The summary RR (95% CI) was 1.02 (0.71–1.48, I^2^ = 42[0–89]%) for vegans vs. non-vegetarians (Figs. 2b and 6b).

### Pancreatic cancer

Five prospective studies (four risk estimates, four publications, 1581 cases and 585,590 participants) [12, 15, 19, 23] were included in the analysis of vegetarian diets and pancreatic cancer. The summary RR (95% CI) was 0.77 (0.62–0.95, I^2^ = 0[0–11]%) for vegetarians vs. non-vegetarians (Figs. 2a and 3f).

### Lung cancer

Six prospective studies (five risk estimates, five publications, 4240 cases, 594,633 participants) [10, 12, 15, 18, 19] were included in the analysis of vegetarian diets and lung cancer. The summary RR (95% CI) was 0.85 (0.70–1.04, I^2^ = 20[0–71]%) for vegetarians vs. non-vegetarians (Figs. 2a and 4a). There was no indication of publication bias with Egger’s test (*p* = 0.75) (Supplementary Fig. 4).

### Melanoma

Four prospective studies (three risk estimates, three publications, 2575 cases, 550,225 participants) [12, 15, 19] were included in the analysis of vegetarian diets and melanoma incidence. The summary RR was 0.79 (95% CI: 0.66–0.94, I^2^ = 0[0–30]%) for vegetarians vs. non-vegetarians (Figs. 2a and 4b).

### Breast cancer

Seven prospective studies (six risk estimates, six publications, 10,453 cases, 411,391 participants) [12, 14–18] were included in the analysis of vegetarian diets and breast cancer. The summary RR (95% CI) was 0.92 (0.86–0.99, I^2^ = 0[0–59]%) for vegetarians vs. non-vegetarians (Figs. 2a and 4c). There was indication of publication bias with Egger’s test (*p* = 0.02) (Supplementary Fig. 5), but this was explained by one outlying study (18) and when excluded Egger’s test was attenuated (*p* = 0.22), and the observed association was slightly attenuated (summary RR = 0.93, 95% CI: 0.86–1.00, I^2^ = 0[0–59]%).

Two [17, 19] and three [17–19] cohort studies were included in the analysis of vegetarian diets and premenopausal and postmenopausal breast cancer risk, respectively, and the summary RR was 0.97 (0.79–1.19, I^2^ = 0[0–0]%) for premenopausal breast cancer and 0.81 (0.69–0.95, I^2^ = 0[0–26]%) for postmenopausal breast cancer (Figs. 2a and 4c).

Four prospective studies (three risk estimates, three publications, 1821 cases, 118,048 participants) [15, 16, 38] were included in the analysis of vegan diets and breast cancer incidence. The summary RR (95% CI) was 0.80 (0.64–1.00, I^2^ = 0[0–0]%) for vegans vs. non-vegetarians (Fig. 6c). When using results at age 65 from the Adventist Health Study II, the summary RR was 0.76 (0.63–0.92, I^2^ = 0[0–41]%).

### Endometrial cancer

Five prospective studies (four risk estimates, four publications, 1767 cases, 336,439 participants) [10, 12, 15, 19] were included in the analysis of vegetarian diets and endometrial cancer. The summary RR (95% CI) was 0.91 (0.77–1.07, I^2^ = 0[0–0]%) for vegetarians vs. non-vegetarians (Figs. 2a and 4d).

### Ovarian cancer

Five prospective studies (four risk estimates, four publications, 1354 cases, 329,379 participants) [12, 15, 19, 29] were included in the analysis of vegetarian diets and ovarian cancer. The summary RR was 0.83 (0.68–1.02, I^2^ = 0[0–66]%) for vegetarians vs. non-vegetarians (Figs. 2a and 4e).

### Prostate cancer

Six prospective studies (five risk estimates, five publications, 11735 cases, 280,249 participants) [10, 14, 15, 18, 21] were included in the analysis of vegetarian diets and prostate cancer. The summary RR (95% CI) was 0.87 (0.75–1.00, I^2^ = 43[0–80]%) for vegetarians vs. non-vegetarians (Figs. 2a and 4f). There was no indication of publication bias with Egger’s test (*p* = 0.50) (Supplementary Fig. 6).

Three prospective studies (two risk estimates, two publications, 1676 cases, 43,408 participants) [15, 21] were included in the analysis of vegan diets and prostate cancer risk. The summary RR (95% CI) was 0.87 (0.50–1.49, I^2^ = 49[0–90]%) for vegans vs. non-vegetarians (Figs. 2b and 6d). When using results at age 65 from the Adventist Health Study II, the summary RR was 0.58 (0.43–0.78, I^2^ = 0[0–0]%).

### Kidney cancer

Four prospective studies (three risk estimates, three publications, 1483 cases, 550,225 participants) [12, 15, 19] were included in the analysis of vegetarian diets and kidney cancer. The summary RR was 0.85 (0.64–1.11, I^2^ = 0[0–0]%) for vegetarians vs. non-vegetarians (Figs. 2a and 5a).

### Bladder cancer

Five prospective studies (four risk estimates, four publications, 2416 cases, 584,423 participants) [12, 15, 19, 30] were included in the analysis of vegetarian diets and bladder cancer. The summary RR was 0.78 (0.63–0.98, I^2^ = 0[0–36]%) for vegetarians vs. non-vegetarians (Figs. 2a and 5b).

### Brain cancer

Five prospective studies (four risk estimates, four publications, 986 cases, 584,225 participants) [12, 15, 19, 36] were included in the analysis of vegetarian diets and brain cancer. The summary RR was (0.99, 0.73–1.34, I^2^ = 0[0–65]%) for vegetarians vs. non-vegetarians (Figs. 2a and 5c).

### Non-Hodgkin’s lymphoma

Four prospective studies (three risk estimates, three publications, 2276 cases, 550,225 participants) [12, 15, 19] were included in the analysis of vegetarian diets and non-Hodgkin’s lymphoma. The summary RR was 0.76, 0.63–0.93, I^2^ = 0[0–19]%) for vegetarians vs. non-vegetarians (Figs. 2a and 5d).

### Multiple myeloma

Four prospective studies (three risk estimates, three publications, 1130 cases, 550,225 participants) [12, 15, 19] were included in the analysis of vegetarian diets and multiple myeloma. The summary RR was 0.75, 0.32–1.77, I^2^ = 0[0–96]%) for vegetarians vs. non-vegetarians (Figs. 2a and 5e).

### Leukemia

Four prospective studies (three risk estimates, three publications, 1442 cases, 550,225 participants) [12, 15, 19] were included in the analysis vegetarian diets and leukemia. The summary RR was 0.95 (0.74–1.21, I^2^ = 0[0–10]%) for vegetarians vs. non-vegetarians (Figs. 2a and 5f).

### Impact of BMI-adjustment, sensitivity analyses, subgroup analyses and E-values

We compared BMI-unadjusted and BMI-adjusted results for the associations between vegetarian and vegan diets and cancer risk and the association between vegetarian diets and total cancer was attenuated from 0.87 (0.84–0.91) to 0.92 (0.87–0.96) and the association between vegan diets and total cancer was attenuated from 0.77 (0.70–0.85) to 0.82 (0.74–0.91) when further adjusted for BMI and similar trends were observed for most individual cancer sites (Supplementary Fig. 13–14).

The studies on vegan diets and cancer appeared to deviate in sensitivity analyses suggesting the results on colorectal and prostate cancer may not have been representative for the overall evidence base (Supplementary text 3).

E-values for vegetarians ranged from 1.39 (lower CI: 1.11) for breast cancer to 3.04 (lower CI: 1.60) for stomach cancer (Supplementary Table 20) and for vegans ranged from 1.81 (lower CI: 1.00) for breast cancer to 1.92 (lower CI: 1.63) for total cancer (Supplementary Table 20).

There was no indication of heterogeneity between subgroups in the analyses of vegetarian diets and colorectal, colon, rectal, lung, breast and prostate cancers with meta-regression analyses (Supplementary Tables 21–22). Heterogeneity was in general low across analyses except for the analysis of vegetarian diets and prostate cancer and multiple myeloma, and of vegan diets and colorectal cancer and prostate cancer (Fig. 2), but out of these, subgroup analyses were only possible for vegetarian diets and prostate cancer because of limited data. In the analysis of vegetarian diets and prostate cancer, the I^2^-value was reduced to 5% and the summary RR was 0.80 (0.69–0.93, *n* = 4 risk estimates) in the subgroup of studies with the highest study quality grading (Supplementary Table 22).

The associations between vegetarian diets and colorectal, lung, breast and prostate cancers were not fully robust to the influence of single studies (Supplementary Fig. 7–12).

### WCRF grading

Using WCRF criteria for evaluating strength of evidence (Supplementary Table 23) we graded the evidence of a causal relationship between vegetarian diets and total cancer, colorectal cancer, colon cancer, total breast cancer, and postmenopausal breast cancer as probable (Supplementary Table 24). We graded the evidence for stomach, proximal colon, pancreatic, bladder, melanoma and non-Hodgkin’s lymphoma to be limited-suggestive of a reduced risk (primarily because of limited data), and for the remaining cancers we considered the evidence limited - no conclusion mainly because of limited data and imprecise risk estimates. We graded the evidence of a causal relationship between vegan diets and total and breast cancer as limited-suggestive, while that for colorectal and prostate cancers was graded as limited-no conclusion (Supplementary Table 25). A justification for the evidence grading is provided in Supplementary Table 26.

## Discussion

This systematic review and meta-analysis of cohort studies found a reduced risk of total cancer (13%), stomach (45%), colorectal (14%) and subsites (colon; 21%, and proximal colon; 45%), pancreatic (23%), breast (8%) and postmenopausal breast (19%), and bladder (21%) cancers, melanoma (21%), and non-Hodgkin’s lymphoma (24%) among vegetarians when compared to non-vegetarians. Suggestive non-statistically significant inverse associations were observed for several other cancer sites (lung, ovarian and prostate cancers), but these associations need further investigation in future studies. In addition, vegan diets were associated with a reduced risk of total cancer (23%) and breast cancer (20%), but no association was observed for colorectal and prostate cancer. When using alternative estimates from the Adventist Health Study II at age 65, the reduction in breast cancer risk was slightly strengthened (24%) and a reduced risk was also observed for prostate cancer (42%) among vegans.

The current findings are consistent with a few previous meta-analyses that reported reduced total cancer incidence in vegetarians and vegans vs. non-vegetarians [39–41], but is to our knowledge the first to analyse all specific cancer sites with available data and to report a reduction in risk of seven specific cancer types in addition to total cancer among vegetarians vs. non-vegetarians.

Several potential mechanisms could explain the observed associations between vegetarian and vegan diets and reduced cancer incidence. Differences in body weight could contribute to the observed associations as adiposity is an important risk factor for at least 12 different cancer sites [5], and possibly several additional cancers [42]. Vegetarian and vegan diets have been shown in randomized controlled trials to lead to weight loss when compared to non-vegetarian diets, with a mean difference of around 3.4 kg reported in one meta-analysis [31], and long-term cohort studies have reported increased weight gain over time with higher intake of red and processed meat and poultry and lower intakes of fruits, vegetables, whole grains and nuts [43–45]. Some of the observed associations between vegetarian and vegan diets and cancer risk were slightly stronger when not adjusted for BMI compared to when adjusted for BMI, and suggested differences in baseline adiposity explains 22% and 42% of the observed reduction in overall cancer risk among vegetarians and vegans, respectively, however, the proportion explained by BMI differed across cancer sites.

There is evidence of reduced risk of type 2 diabetes in vegetarians [46], and type 2 diabetes is an established risk factor for at least 6 cancer types [47]. There is also evidence that inflammation [48] and hormonal factors [49] plays an important role in cancer development. Several studies and reviews found vegetarian diets were associated with reduced insulin resistance and fasting insulin [50], lower C-reactive protein, fibrinogen and leukocytes [51, 52], and lower blood concentrations or urinary levels of estradiol and higher levels of sex-hormone binding globulin (SHBG) [53–57]. An intervention study using a mostly plant-based diet reported significantly increased SHBG, a borderline significant reduction in free estradiol, and reduced insulin, fasting glucose, C-peptide, growth-hormone binding protein, IGFBP-1, and IGFBP-2 [58]. Differences have also been reported in the microbiota of vegetarians compared to non-vegetarians [59–62] that may be beneficial in relation to cancer risk [63, 64].

Dietary differences between vegetarians and non-vegetarians are likely to contribute to the observed associations independently of BMI, with red and processed meat intake being an established risk factor for colorectal cancer [65], and a recent meta-analysis additionally reporting increased risk of liver, lung, breast, and endometrial cancers [9] with red and processed meat intake. Some studies have reported positive associations between red and/or processed meat intake and stomach [66], pancreatic [67], prostate [68, 69], and bladder cancer [70–72], and between red meat or poultry and non-Hodgkin’s lymphoma [69, 73, 74], although the evidence is not entirely consistent [9]. Higher red and processed meat intake can increase cancer risk due to higher levels of heme-iron, saturated fat and cholesterol, heterocyclic amines and polycyclic aromatic hydrocarbons formed during cooking, nitrosamines formed from nitrites and nitrates in processed meat, through adverse impacts on the microbiota, increased weight gain and metabolic perturbations, and through increased risk of various predisposing diseases (e.g. colorectal adenoma, diabetes, liver disease, gallstones, pancreatitis) [75–80]. A higher intake of fiber and whole grains [7, 81] and fruits and vegetables [8, 33, 82, 83] has also been associated with reduced risk of total, colorectal, and breast cancers, and possibly other cancers [84–86]. The slightly stronger association between vegan diets and reduced overall cancer risk and some specific cancers than for vegetarians may be explained by dietary differences between the groups, e.g. either higher intake of plant foods or avoidance of other animal products (such as dairy and eggs) or a combination of these. Dairy products have been associated with reduced colorectal cancer risk [87], but increased prostate cancer risk [88] and could contribute to differences between vegetarians and vegans for these two and possibly other cancers. Calcium and insulin-like growth factor-1 have been thought to contribute to the association between dairy and reduced colorectal [87], and increased prostate cancer risk [88], respectively. The lower levels of circulating IGF-1 observed in vegans [89, 90] is consistent with the positive associations observed between dairy or milk consumption and IGF-1 levels [91–93].

The current analysis may have some limitations that need to be discussed. Although confounding by other risk factors could be an issue, as vegetarians and vegans in general are relatively health conscious, most studies adjusted for other important risk factors such as smoking, alcohol and physical activity. In addition, similar results were observed in the Adventist Health Studies, a population with a very low prevalence of alcohol consumption and tobacco smoking [94], suggesting residual confounding by alcohol or smoking may be less likely to explain the observed associations in those studies. Among the cancers for which we observed significant associations, the estimated E-values ranged from 1.39 (lower CI: 1.11) for breast cancer to 3.04 (lower CI: 1.60) for stomach cancer, with most of the estimates around 1.8–1.9, suggesting an unadjusted confounder (or the joint impact of several unadjusted confounders) would have to be relatively strongly associated with both a vegetarian diet and cancer risk to fully explain away the observed associations. Although we cannot exclude such a possibility with complete certainty, we consider this less likely. Although we were not able to assess the quality of the vegetarian and vegan diets (e.g. if diets contained more whole plant foods or unhealthier items like refined grains, fast foods and sugary drinks), it seems likely given the results, that the majority may have had a decent diet quality, and the findings are in general consistent with reported food group intakes between diet groups [10, 95–97]. Heterogeneity between studies is expected as studies have been conducted in different populations with different background dietary patterns, different detail of the diet assessment tools, differences in methodological issues like adjustment for confounders and differences in the adherence to these diets over time. However, we observed little heterogeneity in the results both overall and in subgroup analyses. Publication bias can also affect meta-analyses of published studies, but we found little evidence of publication bias in these analyses. Only in the analysis of vegetarian diets and breast cancer did we find some indication of publication bias, but this seemed to be driven by one outlying study, which did not substantially influence the summary estimate when excluded. The limited number of studies limited our ability to test for publication bias and conduct subgroup and sensitivity analyses.

Measurement errors in dietary intake at baseline and changes in dietary intake during follow-up could have affected the results. Most of the studies used food frequency questionnaires to assess dietary intake. However, few studies to date have had repeated measures of dietary intake. In the EPIC-Oxford study and Oxford Vegetarian Study 73% of participants who completed a second dietary assessment 5 years after baseline remained in the same diet groups [98], while 27% changed diet group, and it is likely that a larger proportion of participants may have changed their diet with additional follow-up. A stronger inverse association was observed between a vegetarian diet and overall cancer mortality when excluding subjects who changed diet groups during follow-up instead of using only baseline data (HRs of 0.81, 0.71–0.93 vs. 0.90, 0.80–1.03) [98]. Other studies have reported stronger associations between red and processed meat consumption and total cancer mortality when using repeated vs. baseline only measurements (HRs of 1.19, 1.17, and 1.14 when using repeated measures vs. 1.08, 1.04, and 1.08 when using baseline only measures for the highest vs. lowest quintile of total red meat, unprocessed red meat, and processed red meat consumption, respectively) [99]. In the Swedish Mammography Cohort, the HRs for high red meat intake in relation to pancreatic cancer were 1.30 (0.77–2.31) when using only a baseline measurement, 1.73 (0.99–2.98) with updated averages of two measurements and 2.63 (1.02–6.84) when comparing participants with a consistently high intake with those with a consistently low intake [100]. Since all studies included in this meta-analysis only used a baseline dietary assessment to analyse the association between vegetarian and vegan diets and cancer risk it is likely that the observed associations reported here are conservative estimates of the true underlying association. Any additional studies using repeated dietary assessments and addressing the impact of long-term adherence to vegetarian and vegan diets (also throughout the follow-up) in relation to cancer incidence may be informative.

We had a limited number of studies included in most analyses and may have had insufficient statistical power to detect statistically significant associations with some cancers (e.g. distal colon, rectal, lung, endometrial, ovarian, prostate, and kidney cancers). This also precluded subgroup and sensitivity analyses as well as testing for publication bias in several analyses, and particularly in the analysis of vegans. Heterogeneity expressed by small I^2^-values, was generally low, however, several of the meta-analyses were based on few studies, and therefore, the ability of I^2^ to reliably detect heterogeneity is limited. Given the low number of studies in vegans, we also cannot exclude some degree of chance variation as the two studies reporting on vegan diets were not completely representative of the overall results for vegetarians in relation to colorectal and prostate cancer. For vegetarian diets and colorectal cancer, most studies showed associations in the direction of reduced risk, except for the combined analysis of EPIC-Oxford and Oxford Vegetarian Study, which appeared to deviate. Additional larger studies on vegans and cancer risk are needed as the current results may be considered preliminary due to the few available studies. Lastly, although we did develop a protocol for this project as part of a grant application for funding for the project, this was not registered a-priori.

Strengths of the current analysis includes the comprehensive search, analyses across a large number of cancer types, multiple subgroup and sensitivity analyses including use of E-values, and the high study quality of the included studies. These findings may have important public health implications and provide further support for the adoption of primarily plant-based diets in cancer prevention. Given the relatively low prevalence of vegetarians and vegans generally in many populations, the attributable risk could be sizeable, e.g. around 13% and 23% for total cancer, respectively if the results from the studies included are representative for the general population, however, red and processed meat consumption among the nonvegetarians in these studies is lower and intake of plant foods is higher than in the general population [10, 95–97], thus it is possible that these estimates could be conservative. Further and larger studies are needed to further investigate the observed associations, associations across less common and less investigated cancers and associations with vegan diets, which were investigated in a limited number of studies, and in general there were relatively few vegans included across studies. Nevertheless, the findings are largely consistent with current evidence of food groups and cancer risk, suggesting the adoption of much more plant-based diets such as vegetarian and vegan diets could play an important role in cancer prevention.

In conclusion, vegetarian diets were associated with reductions in the relative risk of total cancer (13%), stomach (43%), colorectal (14%), pancreatic (23%), breast (10%), and bladder (21%) cancers, melanoma (21%) and non-Hodgkin’s lymphoma (23%). Vegan diets were associated with a 23% reduction in risk of total cancer and a 20% reduction in breast cancer risk. Although further studies are needed to clarify the associations between vegetarian and vegan diets across less investigated cancer sites, these findings support a beneficial role of vegetarian and vegan diets in cancer prevention and provide further support for recommendations to adopt much more plant-based dietary patterns in the general population.

## Supplementary Information

Below is the link to the electronic supplementary material.

Supplementary Material 1

## Declarations

### Research involving human and animal participants

Patients and/or the public were not involved in the design, or conduct, or reporting, or dissemination plans of this research.
