# Supplementary material for: Vegetarian and vegan diets and cancer incidence: a systematic review and meta-analysis of prospective studies
Source: Eur J Epidemiol. 2026 Mar 25;41(5):531–45. doi: 10.1007/s10654-026-01380-8 (PMC13332899; doi:10.1007/s10654-026-01380-8)
Supplement: Supplementary file 1 — Supplementary Material 1 [file 10654_2026_1380_MOESM1_ESM.docx]

Supplement for:

Aune D, Schlesinger S, Sobiecki JG. Vegetarian and vegan diets and cancer incidence: a systematic review and meta-analysis of prospective studies.

Supplementary text 1. Search strategy in PubMed and Embase databases

PubMed search strategy:

1) (vegetarian or vegan or adventist)

2) (Oral or pharyngeal or pharynx or oropharyngeal or oropharynx or hypopharyngeal or hypopharynx or nasal or paranasal sinus or Nasopharyngeal or nasopharynx or Laryngeal or larynx or Esophageal or esophagus or oesophageal or oesophagus or Upper aerodigestive tract or head and neck or Lung or respiratory or Stomach or gastric or Small intestinal or small intestine or small bowel or Pancreatic or pancreas or Liver or hepatocellular or Gallbladder or Bile duct or Colon or rectal or rectum or colorectal or colorectum or large bowel or Breast or mammary or Ovarian or ovary or Endometrial or endometrium or corpus uteri or uterine or Cervical or cervix or Prostate or Testicular or testes or penis or penile or Kidney or renal or renal cell or adrenal or Bladder or urothelial or urinary tract or Brain or Thyroid or anal)

3) (Cancer or carcinoma or neoplasm or tumor or tumour)

4) (Cholangiocarcinoma or lymphoma or non-Hodgkins lymphoma or non-Hodgkin lymphoma or Hodgkins lymphoma or Hodgkin lymphoma or Hodgkin disease or leukemia or myeloma or melanoma or glioma or meningioma or sarcoma)

5) 2 AND 3

6) 5 OR 3 OR 4

7) 1 AND 6

Embase search strategy:

1) vegetarian/ or vegan/ or adventist/

2) (vegetarian or vegan or adventist).ab,ti.

3) Oral/ or pharyngeal/ or pharynx/ or oropharyngeal/ or oropharynx/ or hypopharyngeal/ or hypopharynx/ or nasal/ or paranasal sinus/ or Nasopharyngeal/ or nasopharynx/ or Laryngeal/ or larynx/ or Esophageal/ or esophagus/ or oesophageal/ or oesophagus/ or Upper aerodigestive tract/ or Lung/ or respiratory/ or Stomach/ or gastric/ or Small intestinal/ or small intestine/ or small bowel/ or Pancreatic/ or pancreas/ or Liver/ or hepatocellular/ or Gallbladder/ or Bile duct/ or Colon/ or rectal/ or rectum/ or colorectal/ or colorectum/ or large bowel/ or Breast/ or mammary/ or Ovarian/ or ovary/ or Endometrial/ or endometrium/ or corpus uteri/ or uterine/ or Cervical/ or cervix/ or Prostate/ or Testicular/ or testes/ or penis/ or penile/ or Kidney/ or renal/ or renal cell/ or adrenal/ or Bladder/ or urothelial/ or urinary tract/ or Brain/ or Thyroid/ or anal/

4) (Oral or pharyngeal or pharynx or oropharyngeal or oropharynx or hypopharyngeal or hypopharynx or nasal or paranasal sinus or Nasopharyngeal or nasopharynx or Laryngeal or larynx or Esophageal or esophagus or oesophageal or oesophagus or Upper aerodigestive tract or head or neck or Lung or respiratory or Stomach or gastric or Small intestinal or small intestine or small bowel or Pancreatic or pancreas or Liver or hepatocellular or Gallbladder or Bile duct or Colon or rectal or rectum or colorectal or colorectum or large bowel or Breast or mammary or Ovarian or ovary or Endometrial or endometrium or corpus uteri or uterine or Cervical or cervix or Prostate or Testicular or testes or penis or penile or Kidney or renal or renal cell or adrenal or Bladder or urothelial or urinary tract or Brain or Thyroid or anal).ab,ti.

5) Cancer/ or carcinoma/ or neoplasm/ or tumor/ or tumour/

6) (Cancer or carcinoma or neoplasm or tumor or tumour).ab,ti.

7) Cholangiocarcinoma/ or lymphoma/ or non-Hodgkins lymphoma/ or non-Hodgkin lymphoma/ or Hodgkins lymphoma/ or Hodgkin lymphoma/ or Hodgkin disease/ or leukemia/ or myeloma/ or melanoma/ or glioma/ or meningioma/ or sarcoma/

8) (Cholangiocarcinoma or lymphoma or non-Hodgkins lymphoma or non-Hodgkin lymphoma or Hodgkins lymphoma or Hodgkin lymphoma or Hodgkin disease or leukemia or myeloma or melanoma or glioma or meningioma or sarcoma).ab,ti.

9) 1 or 2

10) 3 or 4

11) 5 or 6

12) 7 or 8

13) 10 and 11

14) 11 or 12 or 13

15) 9 and 14

Supplementary text 2. Modified Newcastle Ottawa Scale

The version applied include the following modifications that, in our view, address important limitations of NOS and can potentially add value to the estimated score: 1) the point regarding representativeness was removed, as it is not relevant for study quality; 2) scoring 0.25 point per confounding factor adjusted for, up to a maximum of 2 points, instead for giving 2 points for adjustments for two confounders. No points given for adjustment for potential mediators including BMI, diabetes or food groups characteristic of the diet groups. This was deemed justifiable, as studies with relatively crude adjustment (e.g. for age and sex) could still receive, in the original scale, a maximum score but could still be prone to confounding; and 3) for the outcome assessment, we allocated one point for studies with linkage to cancer registries or medical records. This modified NOS gave a total score range from 0 to 8, instead of the range of 0 to 9 of the original scale.

Supplementary text 3. Sensitivity analysis

We repeated the analysis in vegetarians restricted to the studies included in the analysis of vegan diets to check whether studies reporting on vegan diets showed results consistent with the overall results. The summary RRs for vegetarians vs. non-vegetarians when restricted to the studies in the analysis of vegans was 0.88 (95% CI: 0.84-0.92, I^2^=0%, n=2) for total cancer, 0.90 (95% CI: 0.69-1.17, I^2^=72.5%, n=2) for colorectal cancer, 0.92 (0.82-1.04, I^2^=39.4%, n=3) for breast cancer, and 0.94 (95% CI: 0.83-1.06, I^2^=7.6%, n=2) for prostate cancer, with results being similar for total and breast cancer, while associations were weaker and less clear for colorectal and prostate cancer when compared to the main analysis. This suggests the studies on vegans may not have been representative for the overall evidence base for colorectal and prostate cancer.

Supplementary Table 1. List of excluded studies and exclusion reasons

| Exclusion reason | Reference number |
| --- | --- |
| Abstract | (1-3) |
| Cancer recurrence/survival | (4-6) |
| Case-control study | (7-35) |
| Case only study | (36, 37) |
| Comment, editorial, letter | (38-51) |
| Duplicate | (52-60) |
| Meta-analysis | (61-69) |
| News | (70) |
| Not relevant data | (71, 72) |
| Not relevant exposure | (73-126) |
| Not relevant outcome | (127-154) |
| Protocol | (155-159) |
| Review | (160-225) |

Reference List

(1) Orlich M, Singh P, Sabate J, Fan J, Fraser G. Vegetarian diet patterns and mortality: Early findings from adventist health study 2. American Journal of Epidemiology Conference: 45th Annual Meeting of the Society for Epidemiologic Research, SER 2012;(var.pagings):15.

(2) Gharibvand L, Ghamsary M, Beeson WL, Knutsen R, Soret S, Knutsen S. Does diet modify the association between lung cancer and ambient particulate air pollution? European Respiratory Journal Conference: European Respiratory Society Annual Congress 2016;(Supplement 60):01.

(3) Parra-Soto S, Petermann-Rocha F, Pell JP, Ho FK, Celis-Morales C. The association of type of diet and all-cause cancer incidence and mortality: prospective study from UK Biobank. Clinical Nutrition ESPEN Conference: 42nd ESPEN Virtual Congress Virtual, Online 40 (pp 664), 2020;December.

(4) Kunnavuttivanich V, Pramyothin P, Ithimakin S. Association between dietary patterns and disease recurrence in Thai colorectal cancer patients. Medicine (Baltimore) 2020 Mar;99(11):e19522.

(5) Kunnavuttivanich V, Pramyothin P, Ithimakin S. Association between dietary patterns with disease recurrence in Thai colorectal cancer patients. Annals of Oncology Conference: European Society for Medical Oncology Asia Congress, ESMO 2018;(Supplement 9):November.

(6) Kopanitsa G, Metsker O, Bolgova E, Kovalchuk S. Lifestyle Cancer Survival Predictors: Influence of Vegetarian Diet on the Relapse of Endometrial Cancer. Studies in health technology and informatics 285 (pp 193;27.

(7) Hirayama T. An epidemiological study of oral and pharyngeal cancer in Central and South-East Asia. Bull World Health Organ 1966;34(1):41-69.

(8) Rao DN, Sanghvi LD, Desai PB. Epidemiology of esophageal cancer. Semin Surg Oncol 1989;5(5):351-4.

(9) Rao DN, Ganesh B, Rao RS, Desai PB. Risk assessment of tobacco, alcohol and diet in oral cancer--a case-control study. Int J Cancer 1994 Aug 15;58(4):469-73.

(10) Rao DN, Ganesh B, Desai PB. Role of reproductive factors in breast cancer in a low-risk area: a case-control study. Br J Cancer 1994 Jul;70(1):129-32.

(11) Rao DN, Desai PB. Risk assessment of tobacco, alcohol and diet in cancers of base tongue and oral tongue--a case control study. Indian J Cancer 1998 Jun;35(2):65-72.

(12) Rao DN, Desai PB, Ganesh B. Alcohol as an additional risk factor in laryngopharyngeal cancer in Mumbai--a case-control study. Cancer Detect Prev 1999;23(1):37-44.

(13) Bala DV, Patel DD, Duffy SW, Cherman S, Patel PS, Trivedi J, et al. Role of Dietary Intake and Biomarkers in Risk of Breast Cancer: A Case Control Study. Asian Pac J Cancer Prev 2001;2(2):123-30.

(14) Dos SS, I, Mangtani P, McCormack V, Bhakta D, Sevak L, McMichael AJ. Lifelong vegetarianism and risk of breast cancer: a population-based case-control study among South Asian migrant women living in England. Int J Cancer 2002 May 10;99(2):238-44.

(15) Chen YC, Chiang CI, Lin RS, Pu YS, Lai MK, Sung FC. Diet, vegetarian food and prostate carcinoma among men in Taiwan. Br J Cancer 2005 Oct 31;93(9):1057-61.

(16) Gangane N, Chawla S, Anshu, Gupta SS, Sharma SM. Reassessment of risk factors for oral cancer. Asian Pac J Cancer Prev 2007 Apr;8(2):243-8.

(17) Subapriya R, Thangavelu A, Mathavan B, Ramachandran CR, Nagini S. Assessment of risk factors for oral squamous cell carcinoma in Chidambaram, Southern India: a case-control study. Eur J Cancer Prev 2007 Jun;16(3):251-6.

(18) Madani AH, Jahromi AS, Dikshit M, Bhaduri D. Risk assessment of tobacco types and oral cancer. American Journal of Pharmacology and Toxicology 5(1) (pp 9-13), 2010;2010.

(19) Kang HW, Lee JK, Kim JH, Koh MS, Lee JH. Vegetarianism as a protective factor of development of colorectal adenoma: A cross sectional, case-control study. Gastroenterology Conference: Digestive Disease Week, DDW 2011;(var.pagings):May.

(20) Laroiya I, Pankaja S, Mittal S, Kate V. A study of Helicobacter pylori infection, dietary pattern and habits in patients with gastric cancer in South India. Asian Pacific Journal of Tropical Disease 2(1) (pp 24-26), 2012;February.

(21) Kamath R, Mahajan KS, Ashok L, Sanal TS. A study on risk factors of breast cancer among patients attending the tertiary care hospital, in udupi district. Indian J Community Med 2013 Apr;38(2):95-9.

(22) Bhattacharya S, Bhattacharya S, Basu R, Bera P, Halder A. Colorectal cancer: a study of risk factors in a tertiary care hospital of north bengal. J Clin Diagn Res 2014 Nov;8(11):FC08-FC10.

(23) Gautam KA, Muktanand T, Sankhwar SN, Goel A, Sankhwar PL, Rajender S. Functional polymorphisms in the IL6 gene promoter and the risk of urinary bladder cancer in India. Cytokine 2016 Jan;77:152-6.

(24) Chang YJ, Hou YC, Chen LJ, Wu JH, Wu CC, Chang YJ, et al. Is vegetarian diet associated with a lower risk of breast cancer in Taiwanese women? BMC Public Health 2017 Oct 10;17(1):800.

(25) Lu S, Qian Y, Huang X, Yu H, Yang J, Han R, et al. The association of dietary pattern and breast cancer in Jiangsu, China: A population-based case-control study. PLoS One 2017;12(9):e0184453.

(26) Gathani T, Barnes I, Ali R, Arumugham R, Chacko R, Digumarti R, et al. Lifelong vegetarianism and breast cancer risk: a large multicentre case control study in India. BMC Womens Health 2017 Jan 18;17(1):6.

(27) Hou Y-C, Chen L-J, Wu J-H, Wu C-C, Chang Y-J, Chung K-P. Is vegetarian diet associated with a lower risk of breast cancer in Taiwanese women? BMC public health 17(1) (pp 800), 2017;10.

(28) Jayaram DJ, Hakama M, Rayappa PH, Reddy KR. Dietary Risk Factors For Pharyngeal Cancer In South India: A Case-Control Study. Journal of Cancer Research and Therapeutics Conference: 2nd Indian Cancer Congress, ICC 2017;(Supplement 1):2017.

(29) Shridhar K, Singh G, Dey S, Singh DS, Paul Singh GJ, Goodman M, et al. Dietary Patterns and Breast Cancer Risk: A Multi-Centre Case Control Study among North Indian Women. Int J Environ Res Public Health 2018 Sep 6;15(9).

(30) Mishra K, Behari A, Shukla P, Tsuchiya Y, Endoh K, Asai T, et al. Risk factors for gallbladder cancer development in northern India: A gallstones-matched, case-control study. Indian J Med Res 2021 May;154(5):699-706.

(31) Qayyum MA, Farooq T, Baig A, Bokhari TH, Anjum MN, Mahmood MHUR, et al. Assessment of essential and toxic elemental concentrations in tumor and non-tumor tissues with risk of colorectal carcinoma in Pakistan. Journal of Trace Elements in Medicine and Biology 79 (no pagination), 2023;127234.

(32) Nemati M, Shayanfar M, Almasi F, Mohammad-Shirazi M, Sharifi G, Aminianfar A, et al. Dietary patterns in relation to glioma: a case-control study. Cancer Metab 2024 Mar 18;12(1):8.

(33) Lu S, Huang X, Yu H, Yang J, Han R, Su J, et al. Dietary patterns and risk of breast cancer in Chinese women: A population-based case-control study. The Lancet Conference: Chinese Academy of Medical Sciences Health Summit, CAMS 2016;(SPEC.ISS 1):October.

(34) Gathani T, Barnes I. Lifelong vegetarianism and breast cancer risk in India: A multicentre case control study of 2101 women. Cancer Research Conference: 38th Annual CTRC-AACR San Antonio Breast Cancer Symposium San Antonio, TX United States Conference Publication: (var pagings) 76(4 SUPPL 1) (no pagination), 2016;15.

(35) Verma S, Choudhary JK, Singh N, Gupta N, Tripathi MK, Sapna, et al. Epidemiological factors in gallbladder carcinoma in northern India. Indian Journal of Gastroenterology Conference: 54th Annual Conference of the Indian Society of Gastroenterology, ISGCON - 2013;(var.pagings):November.

(36) Kotzev I, Mirchev M, Manevska B, Ivanova I, Kaneva M. Risk and protective factors for development of colorectal polyps and cancer (Bulgarian experience). Hepatogastroenterology 2008 Mar;55(82-83):381-7.

(37) Sinha R, Doval DC, Hussain S, Kumar K, Singh S, Basir SF, et al. Lifestyle and Sporadic Colorectal Cancer in India. Asian Pacific journal of cancer prevention : APJCP 16(17) (pp 7683-7688), 2015;2015.

(38) Malberg K. Are there more cases of colon cancer among vegetarians?. [German]. MMW-Fortschritte der Medizin 151(15) (pp 21), 2009;09.

(39) D'Angelo J. Vegetarian diet affects cancer risk. Nature Reviews Endocrinology 5(8) (pp 415), 2009;2009.

(40) Ernst E. The cancer risk of vegetarians. [German]. MMW-Fortschritte der Medizin 151(37) (pp 23), 2009;2009.

(41) Printz C. Vegetarian diet associated with lower risk of colorectal cancer. Cancer 2015 Aug 15;121(16):2667.

(42) McBride D. Vegetarian Diets May Decrease Risk of Colorectal Cancer. ONS Connect 2015 Jun;30(2):51.

(43) Wise J. Vegetarians have lower risk of colorectal cancers, study finds. BMJ 2015 Mar 9;350:h1313.

(44) Rukavina M. Colorectal cancer: Which vegetarianism lowers the risk of disease? Tumor Diagnostik und Therapie 37(1) (pp 10-12), 2016;2016.

(45) Weller M. Vegetarian diets and cancer risk. BMC Medicine 2020;(1):81.

(46) Key T. Diet and the risk of cancer. BMJ 2007 Nov 3;335(7626):897.

(47) Lindbloom EJ. Long-term benefits of a vegetarian diet. American Family Physician 79(7) (pp 541-542), 2009;01.

(48) Friedrich NA, Freedland SJ, Csizmadi I. Plant-based diets to reduce prostate cancer risk and improve prostate cancer outcomes-ready for prime time? Prostate Cancer Prostatic Dis 2023 Sep;26(3):445-6.

(49) Zevola SA. Re: "Animal product consumption and subsequent fatal breast cancer risk among Seventh-day Adventists". Am J Epidemiol 1989 Feb;129(2):450.

(50) Orlich MJ, Fraser GE. Diet and Colorectal Cancer Incidence-Reply. JAMA Intern Med 2015 Oct;175(10):1727.

(51) Bhopal RS. Diet and Colorectal Cancer Incidence. JAMA Intern Med 2015 Oct;175(10):1726-7.

(52) Sanjoaquin MA, Appleby PN, Thorogood M, Mann JI, Key TJ. Nutrition, lifestyle and colorectal cancer incidence: a prospective investigation of 10998 vegetarians and non-vegetarians in the United Kingdom. Br J Cancer 2004 Jan 12;90(1):118-21.

(53) Key TJ, Appleby PN, Spencer EA, Travis RC, Allen NE, Thorogood M, et al. Cancer incidence in British vegetarians. Br J Cancer 2009 Jul 7;101(1):192-7.

(54) Travis RC, Allen NE, Appleby PN, Spencer EA, Roddam AW, Key TJ. A prospective study of vegetarianism and isoflavone intake in relation to breast cancer risk in British women. Int J Cancer 2008 Feb 1;122(3):705-10.

(55) Key TJ, Appleby PN, Spencer EA, Travis RC, Roddam AW, Allen NE. Cancer incidence in vegetarians: results from the European Prospective Investigation into Cancer and Nutrition (EPIC-Oxford). Am J Clin Nutr 2009 May;89(5):1620S-6S.

(56) Correction to Key et al. Cancer in British vegetarians: updated analyses of 4998 incident cancers in a cohort of 32,491 meat eaters, 8612 fish eaters, 18,298 vegetarians, and 2246 vegans. Am J Clin Nutr 2014;100:378S-85S. Am J Clin Nutr 2022 Jun 7;115(6):1658-9.

(57) Tantamango-Bartley Y, Jaceldo-Siegl K, Fan J, Fraser G. Vegetarian diets and the incidence of cancer in a low-risk population. Cancer Epidemiol Biomarkers Prev 2013 Feb;22(2):286-94.

(58) Orlich MJ, Singh PN, SabatÃ© J, Fan J, Sveen L, Bennett H, et al. Vegetarian dietary patterns and the risk of colorectal cancers. JAMA Intern Med 2015 May;175(5):767-76.

(59) Penniecook-Sawyers JA, Jaceldo-Siegl K, Fan J, Beeson L, Knutsen S, Herring P, et al. Vegetarian dietary patterns and the risk of breast cancer in a low-risk population. Br J Nutr 2016 May 28;115(10):1790-7.

(60) Tantamango-Bartley Y, Knutsen SF, Knutsen R, Jacobsen BK, Fan J, Beeson WL, et al. Are strict vegetarians protected against prostate cancer? Am J Clin Nutr 2016 Jan;103(1):153-60.

(61) Huang T, Yang B, Zheng J, Li G, Wahlqvist ML, Li D. Cardiovascular disease mortality and cancer incidence in vegetarians: a meta-analysis and systematic review. Ann Nutr Metab 2012;60(4):233-40.

(62) Godos J, Bella F, Sciacca S, Galvano F, Grosso G. Vegetarianism and breast, colorectal and prostate cancer risk: an overview and meta-analysis of cohort studies. J Hum Nutr Diet 2017 Jun;30(3):349-59.

(63) Dinu M, Abbate R, Gensini GF, Casini A, Sofi F. Vegetarian, vegan diets and multiple health outcomes: A systematic review with meta-analysis of observational studies. Crit Rev Food Sci Nutr 2017 Nov 22;57(17):3640-9.

(64) Molina-Montes E, Salamanca-FernÃ¡ndez E, Garcia-Villanova B, SÃ¡nchez MJ. The Impact of Plant-Based Dietary Patterns on Cancer-Related Outcomes: A Rapid Review and Meta-Analysis. Nutrients 2020 Jul 6;12(7).

(65) Zhao Y, Zhan J, Wang Y, Wang D. The Relationship Between Plant-Based Diet and Risk of Digestive System Cancers: A Meta-Analysis Based on 3,059,009 Subjects. Front Public Health 2022;10:892153.

(66) Bai T, Peng J, Zhu X, Wu C. Vegetarian diets and the risk of gastrointestinal cancers: a meta-analysis of observational studies. Eur J Gastroenterol Hepatol 2023 Nov 1;35(11):1244-52.

(67) Selinger E, Neuenschwander M, Koller A, Gojda J, KÃ¼hn T, Schwingshackl L, et al. Evidence of a vegan diet for health benefits and risks - an umbrella review of meta-analyses of observational and clinical studies. Crit Rev Food Sci Nutr 2023;63(29):9926-36.

(68) Wang Y, Liu B, Han H, Hu Y, Zhu L, Rimm EB, et al. Correction: Associations Between Plant-Based Dietary Patterns and Risks of Type 2 Diabetes, Cardiovascular Disease, Cancer, and Mortality - A Systematic Review and Meta-analysis (Nutrition Journal, (2023), 22, 1, (46), 10.1186/s12937-023-00877-2). Nutrition Journal 23(1) (no pagination), 2024;6.

(69) Dinu M, Pagliai G, Casini A, Sofi F. Vegetarian, vegan diets and multiple health outcomes: A systematic review with meta-analysis of observational studies. European Heart Journal Conference: European Society of Cardiology, ESC Congress 2016;(Supplement 1):August.

(70) Blackburn GL. "Vegging out" for better health? Vegetarians may have healthier, longer lives than their carnivorous counterparts. Health News 2003 Nov;9(11):8-9.

(71) Paymaster JC, Sanghvi LD, Gangadharan P. Cancer in the gastrointestinal tract in western india. epidemiological study. Cancer 21(2) (pp 279-288), 1968;1968.

(72) Vaishali S. Diet and gastro-intestinal cancer incidence-phase II project. Annals of Oncology Conference: 13th World Congress on Gastrointestinal Cancer, ESMO 2011;(var.pagings):June.

(73) Wynder EL, Lemon FR. Cancer, coronary artery disease and smoking: a preliminary report on differences in incidence between Seventh-day Adventists and others. Calif Med 1958 Oct;89(4):267-72.

(74) Wynder EL, Lemon FR, BROSS IJ. Cancer and coronary artery disease among Seventh-Day Adventists. Cancer 1959 Sep;12:1016-28.

(75) Lemon FR, Walden RT, WOODS RW. CANCER OF THE LUNG AND MOUTH IN SEVENTH-DAY ADVENTISTS. PRELIMINARY REPORT ON A POPULATION STUDY. Cancer 1964 Apr;17:486-97.

(76) Lemon FR, Walden RT. Death from respiratory system disease among Seventh-Day Adventist men. JAMA 1966 Oct 10;198(2):117-26.

(77) Phillips RL. Role of life-style and dietary habits in risk of cancer among seventh-day adventists. Cancer Res 1975 Nov;35(11 Pt. 2):3513-22.

(78) Phillips RL, Kuzma JW. Rationale and methods for an epidemiologic study of cancer among Seventh-Day Adventists. Natl Cancer Inst Monogr 1977 Dec;47:107-12.

(79) Phillips RL, Garfinkel L, Kuzma JW, Beeson WL, Lotz T, Brin B. Mortality among California Seventh-Day Adventists for selected cancer sites. J Natl Cancer Inst 1980 Nov;65(5):1097-107.

(80) Phillips RL. Cancer among Seventh-Day Adventists. J Environ Pathol Toxicol 1980 Mar;3(4 Spec No):157-69.

(81) Waaler HT, Hjort PF. [Longevity among Norwegian Adventists 1960-1977: a message of life style and health?]. Tidsskr Nor Laegeforen 1981 Apr 20;101(11):623-7.

(82) Berkel J, de WF. Mortality pattern and life expectancy of Seventh-Day Adventists in the Netherlands. Int J Epidemiol 1983 Dec;12(4):455-9.

(83) Jensen OM. Cancer risk among Danish male Seventh-Day Adventists and other temperance society members. J Natl Cancer Inst 1983 Jun;70(6):1011-4.

(84) Halvorsen BA, Vellar OD, Svendsen B. [Reduced mortality of cancer and cardiovascular diseases among Seventh Day Adventists. Health benefits of better life style?]. Tidsskr Nor Laegeforen 1985 Aug 30;105(24):1620-5.

(85) Murphy FG, Blumenthal DS, ckson-Smith J, Peay RP. The mortality profile of Black Seventh-day Adventists residing in Metropolitan Atlanta: A pilot study. American Journal of Public Health 80(8) (pp 984-985), 1990;1990.

(86) Fonnebo¸ V, Helseth A. Cancer incidence in Norwegian Seventh-Day Adventists 1961 to 1986. Is the cancer-life-style association overestimated? Cancer 1991 Aug 1;68(3):666-71.

(87) Ullmann D, Phillips RL, Beeson WL, Dewey HG, Brin BN, Kuzma JW, et al. Cause-specific mortality among physicians with differing life-styles. JAMA 1991 May 8;265(18):2352-9.

(88) Fonnebo¸ V. Mortality in Norwegian Seventh-Day Adventists 1962-1986. J Clin Epidemiol 1992 Feb;45(2):157-67.

(89) Grundmann E. Cancer morbidity and mortality in USA Mormons and Seventh-day Adventists. Arch Anat Cytol Pathol 1992;40(2-3):73-8.

(90) Mills PK, Beeson WL, Phillips RL, Fraser GE. Cancer incidence among California Seventh-Day Adventists, 1976-1982. Am J Clin Nutr 1994 May;59(5 Suppl):1136S-42S.

(91) Fonnebo¸ V. The healthy Seventh-Day Adventist lifestyle: what is the Norwegian experience? Am J Clin Nutr 1994 May;59(5 Suppl):1124S-9S.

(92) Thygesen L, Hoff A, Nylandsted L, Hvidt N, Johansen C. Cancer incidence among Danish seventh day adventists. Psycho-Oncology Conference: 11th World Congress of Psycho-Oncology of the International Psycho-Oncology Society, IPOS Vienna Austria Conference Publication: (var pagings) 18(SUPPL 2) (pp S38-S39), 2009;June.

(93) Thygesen LC, Hvidt NC, Hansen HP, Hoff A, Ross L, Johansen C. Cancer incidence among Danish Seventh-day Adventists and Baptists. Cancer Epidemiol 2012 Dec;36(6):513-8.

(94) Oliveira ER, Cade NV, Velten AP, Silva GA, Faerstein E. Comparative study of cardiovascular and cancer mortality of Adventists and non-Adventists from EspÃ­rito Santo State, in the period from 2003 to 2009. Rev Bras Epidemiol 2016 Mar;19(1):112-21.

(95) Thygesen L, Hansen HP, Andreas H, Lone R, Christian HN, Christoffer J. Cancer incidence among Danish Seventh-day Adventists and Baptists. European Journal of Epidemiology Conference: IEA-EEF European Congress of Epidemiology 2012;(var.pagings):September.

(96) Oliveira ERA, Velten APC, Cade NV, De Oliveira CS. Comparison of seventh day adventist mortality with a general population in the Espirito Santo state, Brazil. European Journal of Epidemiology Conference: EuroEpi 2013;(var.pagings):August.

(97) Fraser GE, Shavlik D. Risk factors, lifetime risk, and age at onset of breast cancer. Ann Epidemiol 1997 Aug;7(6):375-82.

(98) Fraser GE, Cosgrove CM, Mashchak AD, Orlich MJ, Altekruse SF. Lower rates of cancer and all-cause mortality in an Adventist cohort compared with a US Census population. Cancer 2020 Mar 1;126(5):1102-11.

(99) Martinez CF, Di Castelnuovo A, Costanzo S, Panzera T, Esposito S, Cerletti C, et al. Pro-Vegetarian Food Patterns and Cancer Risk among Italians from the Moli-Sani Study Cohort. Nutrients 2023 Sep 14;15(18).

(100) Sankar V, Parthasarathy R, Sivakumar V. Association of Dietary Factors and Physical Inactivity with Molecular Subtypes of Breast Cancer- Hospital Based Case-Control Study. Nutr Cancer 2023;75(10):1883-91.

(101) Nadeem S, Dinesh K, Tasneef Z, Bhavna S, Rahul S, Kiran B. Dietary risk factors in gastrointestinal cancers: A case-control study in North India. J Cancer Res Ther 2023 Jul;19(5):1385-91.

(102) Castro F, Parikh R, Eustaquio JC, Derkach A, Joseph JM, Lesokhin AM, et al. Pre-Diagnosis Dietary Patterns and Risk of Multiple Myeloma in the NIH-AARP Diet and Health Study. medRxiv 2023 Sep 23.

(103) Nejad ET, Moslemi E, Souni F, Mahmoodi M, Vali M, Vatanpour M, et al. The association between pro-vegetarian dietary pattern and risk of colorectal cancer: a matched case-control study. BMC Res Notes 2023 Nov 9;16(1):326.

(104) Tarei NN, Safakar HY. Relationship between a plantbased diet and breast cancer: A systematic review. Iranian Journal of Basic Medical Sciences Conference: 2nd International Congress Nutrition: From Laboratory Research to Clinical Studies Mashhad Iran, Islamic Republic of 26(Supplement 1) (pp 398), 2023;2023.

(105) Tarei NN, Safakar HY. Between a plant-based diet and breast cancer: A systematic review. Iranian Journal of Basic Medical Sciences Conference: 2nd International Congress Nutrition: From Laboratory Research to Clinical Studies Mashhad Iran, Islamic Republic of 26(Supplement 1) (pp 306), 2023;2023.

(106) Parikh R, Castro F, Eustaquio JC, Derkach A, Joseph J, Lesokhin AM, et al. Pre-diagnosis dietary patterns and risk of multiple myeloma in the NIH-AARP cohort. Journal of Clinical Oncology Conference: 2023;(16 Supplement):01.

(107) Sciacca S, Lo GA, Asmundo MG, Cimino S, Morgia G, Alshatwi AA, et al. Adherence to Healthy or Unhealthy Pro-Vegetarian Plant-Based Diets Have Different Impact on Prostate Cancer Severity: Preliminary Findings. Nutr Cancer 2024;76(1):98-105.

(108) Zhou L, Zhang R, Yang H, Zhang S, Zhang Y, Li H, et al. Association of plant-based diets with total and cause-specific mortality across socioeconomic deprivation level: a large prospective cohort. Eur J Nutr 2024 Apr;63(3):835-46.

(109) Tammi R, Kaartinen NE, Harald K, Maukonen M, Tapanainen H, Smith-Warner SA, et al. Partial substitution of red meat or processed meat with plant-based foods and the risk of colorectal cancer. Eur J Epidemiol 2024 Apr;39(4):419-28.

(110) Hosseini Y, Hadi SP, Moslemi E, Nouri M, Rajabzadeh-Dehkordi M, Jalali S, et al. Pro-vegetarian dietary pattern and risk of breast cancer: a case-control study. Breast Cancer Res Treat 2024 Jun;205(2):395-402.

(111) Oncina-Canovas A, Torres-Collado L, Garcia-de-la-Hera M, Compan-Gabucio LM, Gonzalez-Palacios S, Signes-Pastor AJ, et al. Pro-vegetarian dietary patterns and mortality by all-cause and specific causes in an older Mediterranean population. J Nutr Health Aging 2024 Apr 20;28(7):100239.

(112) Mahmoodi M, Gabal BC, Mohammadi F, Ibrahim FM, Jalilpiran Y, Nouri M, et al. The association between healthy and unhealthy dietary indices with prostate cancer risk: a case-control study. J Health Popul Nutr 2024 Jun 20;43(1):90.

(113) Yarmand S, Rashidkhani B, Alimohammadi A, Shateri Z, Shakeri M, Sohrabi Z, et al. A healthful plant-based diet can reduce the risk of developing colorectal cancer: case-control study. J Health Popul Nutr 2024 Jul 31;43(1):111.

(114) Bock N, Langmann F, Johnston LW, Ibsen DB, Dahm CC. The Association between the Substitution of Red Meat with Legumes and the Risk of Primary Liver Cancer in the UK Biobank: A Cohort Study. Nutrients 2024 Jul 23;16(15).

(115) Di MM, Augustin LSA, Jenkins DJA, Crispo A, Toffolutti F, Negri E, et al. Adherence to a Cholesterol-Lowering Diet and the Risk of Pancreatic Cancer: A Case-Control Study. Nutrients 2024 Aug 1;16(15).

(116) Turati F, Mignozzi S, Esposito G, Bravi F, D'Angelo A, Alicandro G, et al. Indices of healthy and unhealthy plant-based diets and the risk of selected digestive cancers. Clin Nutr 2025 Jan;44:76-85.

(117) Wei W, Wang S, Yuan Z, Ren Y, Wu J, Gao X, et al. Plant-based diets and the risk of lung cancer: a large prospective cohort study. Eur J Nutr 2025 Feb 1;64(2):73.

(118) Zhu W, Shi Z, Yan X, Lei Z, Wang Q, Lei L, et al. Plant-based dietary patterns, genetic risk, proteome, and lung cancer risk: a large prospective cohort study. Eur J Nutr 2025 Feb 11;64(2):89.

(119) Dong X, Zhang M, Shu J, Li Y, Tan P, Peng T, et al. The quality of plant-based diets and liver cancer incidence and liver disease mortality in the UK Biobank. Clin Nutr ESPEN 2025 Jun;67:541-8.

(120) Souni F, Mansouri F, Jafari F, Sharifi R, PourvatanDoust S, Shateri Z, et al. The association between plant-based diet indices and the risk of breast cancer: a case-control study. J Health Popul Nutr 2025 Apr 19;44(1):127.

(121) Dai YN, Yu EY, Zeegers MP, Wesselius A. The association between diet and bladder cancer risk: a two-sample mendelian randomization. Eur J Nutr 2025 Jun 18;64(5):223.

(122) Oncina-Canovas A, Gonzalez-Palacios S, Notario-Barandiaran L, Torres-Collado L, Signes-Pastor A, de-Madaria E, et al. Adherence to Pro-Vegetarian Food Patterns and Risk of Oesophagus, Stomach, and Pancreas Cancers: A Multi Case-Control Study (The PANESOES Study). Nutrients 2022 Dec 12;14(24).

(123) Gomez-Donoso C, Martinez-Gonzalez MA, Martinez JA, Gea A, Sanz-Serrano J, Perez-Cueto FJA, et al. A provegetarian food pattern emphasizing preference for healthy plant-derived foods reduces the risk of overweight/obesity in the SUN cohort. Nutrients 11(7) (no pagination), 2019;1553.

(124) Martinez-Gonzalez MA, Sanchez-Tainta A, Corella D, Salas-Salvado J, Ros E, Aros F, et al. A provegetarian food pattern and reduction in total mortality in the PrevenciÃ³n con Dieta MediterrÃ¡nea (PREDIMED) study. Am J Clin Nutr 2014 Jul;100 Suppl 1:320S-8S.

(125) Romanos-Nanclares A, Toledo E, SÃ¡nchez-Bayona R, SÃ¡nchez-Quesada C, MartÃ­nez-GonzÃ¡lez MÃ, Gea A. Healthful and unhealthful provegetarian food patterns and the incidence of breast cancer: Results from a Mediterranean cohort. Nutrition 2020 Nov;79-80:110884.

(126) Leone A, MartÃ­nez-GonzÃ¡lez MÃ, Martin-Gorgojo A, SÃ¡nchez-Bayona R, De AR, Bertoli S, et al. Mediterranean diet, Dietary Approaches to Stop Hypertension, and Pro-vegetarian dietary pattern in relation to the risk of basal cell carcinoma: a nested case-control study within the Seguimiento Universidad de Navarra (SUN) cohort. Am J Clin Nutr 2020 Aug 1;112(2):364-72.

(127) Phillips RL, Kuzma JW, Beeson WL, Lotz T. Influence of selection versus lifestyle on risk of fatal cancer and cardiovascular disease among Seventh-day Adventists. Am J Epidemiol 1980 Aug;112(2):296-314.

(128) Kinlen LJ, Hermon C, Smith PG. not relevant outcome - mortality, cohortA proportionate study of cancer mortality among members of a vegetarian society. Br J Cancer 1983 Sep;48(3):355-61.

(129) Phillips RL, Snowdon DA. Association of meat and coffee use with cancers of the large bowel, breast, and prostate among Seventh-Day Adventists: preliminary results. Cancer Res 1983 May;43(5 Suppl):2403s-8s.

(130) Snowdon DA, Phillips RL, Choi W. Diet, obesity, and risk of fatal prostate cancer. Am J Epidemiol 1984 Aug;120(2):244-50.

(131) Hirayama T. Mortality in Japanese with life-styles similar to Seventh-Day Adventists: strategy for risk reduction by life-style modification. Natl Cancer Inst Monogr 1985 Dec;69:143-53.

(132) Kuratsune M, Ikeda M, Hayashi T. Epidemiologic studies on possible health effects of intake of pyrolyzates of foods, with reference to mortality among Japanese Seventh-Day Adventists. Environ Health Perspect 1986 Aug;67:143-6.

(133) Snowdon DA. Animal product consumption and mortality because of all causes combined, coronary heart disease, stroke, diabetes, and cancer in Seventh-day Adventists. Am J Clin Nutr 1988 Sep;48(3 Suppl):739-48.

(134) Mills PK, Beeson WL, Abbey DE, Fraser GE, Phillips RL. Dietary habits and past medical history as related to fatal pancreas cancer risk among Adventists. Cancer 1988 Jun 15;61(12):2578-85.

(135) Mills PK, Annegers JF, Phillips RL. Animal product consumption and subsequent fatal breast cancer risk among Seventh-day Adventists. Am J Epidemiol 1988 Mar;127(3):440-53.

(136) Frentzel-Beyme R, Claude J, Eilber U. Mortality among German vegetarians: first results after five years of follow-up. Nutr Cancer 1988;11(2):117-26.

(137) Chang-Claude J, Frentzel-Beyme R, Eilber U. Mortality pattern of German vegetarians after 11 years of follow-up. Epidemiology 1992 Sep;3(5):395-401.

(138) Chang-Claude J, Frentzel-Beyme R. Dietary and lifestyle determinants of mortality among German vegetarians. Int J Epidemiol 1993 Apr;22(2):228-36.

(139) Thorogood M, Mann J, Appleby P, McPherson K. Risk of death from cancer and ischaemic heart disease in meat and non-meat eaters. BMJ 1994 Jun 25;308(6945):1667-70.

(140) Frentzel-Beyme R, Chang-Claude J. Vegetarian diets and colon cancer: the German experience. Am J Clin Nutr 1994 May;59(5 Suppl):1143S-52S.

(141) Key TJ, Thorogood M, Appleby PN, Burr ML. Dietary habits and mortality in 11,000 vegetarians and health conscious people: results of a 17 year follow up. BMJ 1996 Sep 28;313(7060):775-9.

(142) Key TJ, Fraser GE, Thorogood M, Appleby PN, Beral V, Reeves G, et al. Mortality in vegetarians and non-vegetarians: a collaborative analysis of 8300 deaths among 76,000 men and women in five prospective studies. Public Health Nutr 1998 Mar;1(1):33-41.

(143) Key TJ, Fraser GE, Thorogood M, Appleby PN, Beral V, Reeves G, et al. Mortality in vegetarians and nonvegetarians: detailed findings from a collaborative analysis of 5 prospective studies. Am J Clin Nutr 1999 Sep;70(3 Suppl):516S-24S.

(144) Appleby PN, Key TJ, Thorogood M, Burr ML, Mann J. Mortality in British vegetarians. Public Health Nutr 2002 Feb;5(1):29-36.

(145) Key TJ, Appleby PN, Davey GK, Allen NE, Spencer EA, Travis RC. Mortality in British vegetarians: review and preliminary results from EPIC-Oxford. Am J Clin Nutr 2003 Sep;78(3 Suppl):533S-8S.

(146) Chang-Claude J, Hermann S, Eilber U, Steindorf K. Lifestyle determinants and mortality in German vegetarians and health-conscious persons: results of a 21-year follow-up. Cancer Epidemiol Biomarkers Prev 2005 Apr;14(4):963-8.

(147) Key TJ, Appleby PN, Spencer EA, Travis RC, Roddam AW, Allen NE. Mortality in British vegetarians: results from the European Prospective Investigation into Cancer and Nutrition (EPIC-Oxford). Am J Clin Nutr 2009 May;89(5):1613S-9S.

(148) Orlich MJ, Singh PN, SabatÃ© J, Jaceldo-Siegl K, Fan J, Knutsen S, et al. Vegetarian dietary patterns and mortality in Adventist Health Study 2. JAMA Intern Med 2013 Jul 8;173(13):1230-8.

(149) Appleby PN, Crowe FL, Bradbury KE, Travis RC, Key TJ. Mortality in vegetarians and comparable nonvegetarians in the United Kingdom. Am J Clin Nutr 2016 Jan;103(1):218-30.

(150) Appleby PN, Thorogood M, Mann JI, Key TJ. The Oxford Vegetarian Study: an overview. Am J Clin Nutr 1999 Sep;70(3 Suppl):525S-31S.

(151) Lee CG, Hahn SJ, Song MK, Lee JK, Kim JH, Lim YJ, et al. Vegetarianism as a protective factor for colorectal adenoma and advanced adenoma in Asians. Dig Dis Sci 2014 May;59(5):1025-35.

(152) Blackie K, Bobe G, Takata Y. Vegetarian diets and risk of all-cause mortality in a population-based prospective study in the United States. J Health Popul Nutr 2023 Nov 23;42(1):130.

(153) Elshami M, Albandak M, Alser M, Al-Slaibi I, Ayyad M, Dwikat MF, et al. Differences in Colorectal Cancer Awareness Between Vegetarians and Nonvegetarians: A National Cross-Sectional Study From Palestine. JCO Glob Oncol 2024 Feb;10:e2300400.

(154) Kim J, Wilkens LR, Haiman CA, Le ML, Park SY. Plant-based dietary patterns and mortality from all causes, cardiovascular disease, and cancer: The Multiethnic Cohort Study. Clin Nutr 2024 Jun;43(6):1447-53.

(155) Ferguson JJA, Austin G, Oldmeadow C, Garg ML. Plant-based dietary patterns and cardiovascular disease risk in Australians: the Plant-Based Diet Cohort study protocol. Proceedings of the Nutrition Society Conference: 46th Annual Scientifi c Meeting of the Nutrition Society ofAustralia Perth, WA Australia 82(OCE2) (pp E180), 2023;2023.

(156) Dunneram Y, Lee JY, Watling CZ, Fraser GE, Miles F, Prabhakaran D, et al. Participant characteristics in the Health in Vegetarians Consortium: a collaborative analysis of 11 prospective studies. Proceedings of the Nutrition Society Conference: Summer Conference 2023;(Supplement OCE5):2023.

(157) Dunneram Y, Lee JY, Watling CZ, Fraser GE, Miles F, Prabhakaran D, et al. Methods and participant characteristics in the Cancer Risk in Vegetarians Consortium: a cross-sectional analysis across 11 prospective studies. BMC Public Health 2024 Aug 2;24(1):2095.

(158) Cohort Study on Plant-based Diets (COPLANT Study). clinicaltrials gov (no pagination), 2024;01.

(159) Gilsing AM, Weijenberg MP, Goldbohm RA, Dagnelie PC, van den Brandt PA, Schouten LJ. The Netherlands Cohort Study Meat Investigation Cohort; a population-based cohort over-represented with vegetarians, pescetarians and low meat consumers. Nutr J 2013 Nov 29;12:156.

(160) Loeb S, Borin JF, Venigalla G, Narasimman M, Gupta N, Cole AP, et al. Plant-based diets and urological health. Nature Reviews Urology 22(4) (pp 199;e0119313.

(161) Melina V, Craig W, Levin S. Position of the Academy of Nutrition and Dietetics: Vegetarian Diets. Journal of the Academy of Nutrition and Dietetics 116(12) (pp 1970;01.

(162) Bailie IE. THe first international congress on vegetarian nutrition. Journal of Applied Nutrition 39(2) (pp 97-105), 1987;1987.

(163) Dwyer JT. Health aspects of vegetarian diets. Am J Clin Nutr 1988 Sep;48(3 Suppl):712-38.

(164) Willett WC. Epidemiologic studies of diet and cancer. Medical Oncology and Tumor Pharmacotherapy 7(2-3) (pp 93-97), 1990;1990.

(165) Willett WC, Hunter DJ. Prospective studies of diet and breast cancer. Cancer 1994 Aug 1;74(3 Suppl):1085-9.

(166) Nair P, Mayberry JF. Vegetarianism, dietary fibre and gastro-intestinal disease. Dig Dis 1994 May;12(3):177-85.

(167) Lin C-L. Vegetarianism. Tzu Chi Medical Journal 9(2) (pp 81-89), 1997;1997.

(168) Schlienger J-L, Pradignac A, Boichot G, Simon C. Cancer and vegetarian diet. [French]. Cahiers de Nutrition et de Dietetique 33(2) (pp 83-88), 1998;April.

(169) Segasothy M, Phillips PA. Vegetarian diet: panacea for modern lifestyle diseases? QJM 1999 Sep;92(9):531-44.

(170) Key TJ, Davey GK, Appleby PN. Health benefits of a vegetarian diet. Proc Nutr Soc 1999 May;58(2):271-5.

(171) Willett WC. Convergence of philosophy and science: The Third International Congress on Vegetarian Nutrition. American Journal of Clinical Nutrition 70(3 SUPPL ) (pp 434S-438S), 1999;1999.

(172) Truswell AS. Meat consumption and cancer of the large bowel. Eur J Clin Nutr 2002 Mar;56 Suppl 1:S19-S24.

(173) Sabate J. The contribution of vegetarian diets to human health. Forum Nutr 2003;56:218-20.

(174) Sinha R, Anderson DE, McDonald SS, Greenwald P. Cancer risk and diet in India. J Postgrad Med 2003 Jul;49(3):222-8.

(175) American Dietetic Association, Dietitians of Canada. Position of the American Dietetic Association and Dietitians of Canada: Vegetarian diets. J Am Diet Assoc 2003 Jun;103(6):748-65.

(176) Leitzmann C. Vegetarian diets: what are the advantages? Forum Nutr 2005;(57):147-56.

(177) Penkov A. Vegetarian diet in human health. Acta Medica Bulgarica 32(1) (pp 22-34), 2005;2005.

(178) Phillips F. Vegetarian nutrition. Nutrition Bulletin 30(2) (pp 132-167), 2005;June.

(179) Key TJ, Appleby PN, Rosell MS. Health effects of vegetarian and vegan diets. Proc Nutr Soc 2006 Feb;65(1):35-41.

(180) Ginter E. Vegetarian diets, chronic diseases and longevity. Bratisl Lek Listy 2008;109(10):463-6.

(181) Craig WJ, Mangels AR. Position of the American Dietetic Association: vegetarian diets. J Am Diet Assoc 2009 Jul;109(7):1266-82.

(182) Fraser GE. Vegetarian diets: what do we know of their effects on common chronic diseases? Am J Clin Nutr 2009 May;89(5):1607S-12S.

(183) Freedland SJ, Aronson WJ. Dietary intervention strategies to modulate prostate cancer risk and prognosis. Curr Opin Urol 2009 May;19(3):263-7.

(184) Craig WJ. Health effects of vegan diets. Am J Clin Nutr 2009 May;89(5):1627S-33S.

(185) Lanou AJ, Svenson B. Reduced cancer risk in vegetarians: an analysis of recent reports. Cancer Manag Res 2010 Dec 20;3:1-8.

(186) Madry E, Walkowiak J, mczak-Ratajczak A. Vegan diet-what the physician should bear in mind. Family Medicine and Primary Care Review 12(3) (pp 936-938), 2010;July-September.

(187) Craig WJ, Mangels AR. Position of the American Dietetic Association: Vegetarian Diets. [Spanish]. Actividad Dietetica 14(1) (pp 10-26), 2010;January.

(188) McEvoy CT, Temple N, Woodside JV. Vegetarian diets, low-meat diets and health: a review. Public Health Nutr 2012 Dec;15(12):2287-94.

(189) Lamisse F. Vegetarian diet. [French]. Medecine des Maladies Metaboliques 7(2) (pp 109-113), 2013;March.

(190) Matada NPK, Philippe MM, Koneri R. A study on plant based dietary patterns and cancer risk. International Journal of Pharmaceutical Sciences Review and Research 23(2) (pp 265-278), 2013;45.

(191) Pilis W, Stec K, Zych M, Pilis A. Health benefits and risk associated with adopting a vegetarian diet. Rocz Panstw Zakl Hig 2014;65(1):9-14.

(192) Orlich MJ, Fraser GE. Vegetarian diets in the Adventist Health Study 2: a review of initial published findings. Am J Clin Nutr 2014 Jul;100 Suppl 1(1):353S-8S.

(193) Le LT, Sabate J. Beyond meatless, the health effects of vegan diets: findings from the Adventist cohorts. Nutrients 2014 May 27;6(6):2131-47.

(194) Li D. Effect of the vegetarian diet on non-communicable diseases. J Sci Food Agric 2014 Jan 30;94(2):169-73.

(195) Appleby PN, Key TJ. The long-term health of vegetarians and vegans. Proc Nutr Soc 2016 Aug;75(3):287-93.

(196) Midha S, Chawla S, Garg PK. Modifiable and non-modifiable risk factors for pancreatic cancer: A review. Cancer Letters 381(1) (pp 269-277), 2016;10.

(197) Strohle A, Hahn A. Health outcomes of vegetarian nutrition - An update. [German]. Therapeutische Umschau 73(11) (pp 659-672), 2016;November.

(198) Sobiecki JG. Vegetarianism and colorectal cancer risk in a low-selenium environment: effect modification by selenium status? A possible factor contributing to the null results in British vegetarians. Eur J Nutr 2017 Aug;56(5):1819-32.

(199) Orlich MJ, Chiu THT, Dhillon PK, Key TJ, Fraser GE, Shridhar K, et al. Vegetarian Epidemiology: Review and Discussion of Findings from Geographically Diverse Cohorts. Adv Nutr 2019 Nov 1;10(Suppl_4):S284-S295.

(200) Segovia-Siapco G, Sabate J. Health and sustainability outcomes of vegetarian dietary patterns: a revisit of the EPIC-Oxford and the Adventist Health Study-2 cohorts. European Journal of Clinical Nutrition 72(Supplement 1) (pp 60-70), 2019;July.

(201) Jibu RM, Gayatri DR, Jyothipriya A. A survey on cancer risk among vegetarians and non-vegetarians. Drug Invention Today 12(4) (pp 746-749), 2019;01.

(202) Rivas F, Diaz L, Gimenez R. Dietary intake and cancer risk. [Spanish]. Nutricion Clinica y Dietetica Hospitalaria 25(2) (pp 2019;2005.

(203) Oussalah A, Levy J, Berthezene C, Alpers DH, Gueant JL. Health outcomes associated with vegetarian diets: An umbrella review of systematic reviews and meta-analyses. Clin Nutr 2020 Nov;39(11):3283-307.

(204) Zheng Y, Meng L, Liu H, Sun L, Nie Y, Wu Q, et al. Let food be thy medicine: The role of diet in colorectal cancer: A narrative review. Journal of Gastrointestinal Oncology 13(4) (pp 2020;August.

(205) Struncova D, Dymackova R. Diet and its effect on prostate cancer, with a focus on plant-based diet. [Czech]. Klinicka Onkologie 33(Supplement 1) (pp S52-S59), 2020;2020.

(206) Molina-Montes E, Ubago-Guisado E, Petrova D, Amiano P, Chirlaque MD, Agudo A, et al. The Role of Diet, Alcohol, BMI, and Physical Activity in Cancer Mortality: Summary Findings of the EPIC Study. Nutrients 2021 Nov 28;13(12).

(207) Key TJ, Papier K, Tong TYN. Plant-based diets and long-term health: findings from the EPIC-Oxford study. Proc Nutr Soc 2022 May;81(2):190-8.

(208) DeClercq V, Nearing JT, Sweeney E. Plant-Based Diets and Cancer Risk: What is the Evidence? Curr Nutr Rep 2022 Jun;11(2):354-69.

(209) Karamnova NS, Izmailova OV, Shvabskaya OB, Drapkina OM. Plant-based diets: human and planetary health. [Russian]. Profilakticheskaya Meditsina 25(11) (pp 113-123), 2022;2022.

(210) Gupta N, Patel HD, Taylor J, Borin JF, Jacobsohn K, Kenfield SA, et al. Systematic review of the impact of a plant-based diet on prostate cancer incidence and outcomes. Prostate Cancer and Prostatic Diseases 25(3) (pp 444-452), 2022;September.

(211) Gupta N, Taylor J, Borin J, Jacobsohn K, Kenfield S, Eggener S, et al. Systematic review of the impact of a plant-based diet on prostate cancer incidence and outcomes. Journal of Urology Conference: 117th Annual Meeting of the American Urological Association, AUA 2022;(SUPPL 5):May.

(212) Wang T, Masedunskas A, Willett WC, Fontana L. Vegetarian and vegan diets: benefits and drawbacks. Eur Heart J 2023 Jul 14.

(213) Stefanini M, Bianchelli D, Scandurra F, Mazzetti M, Sangiorgi M. Vegetarian diet and oral health: a narrative review. [Italian]. Dental Cadmos 91(6) (pp 458-469), 2023;June.

(214) Luszczki E, Boakye F, Zielinska M, Deren K, Bartosiewicz A, Oleksy L, et al. Vegan diet: nutritional components, implementation, and effects on adults' health. Front Nutr 2023;10:1294497.

(215) Capodici A, Mocciaro G, Gori D, Landry MJ, Masini A, Sanmarchi F, et al. Cardiovascular health and cancer risk associated with plant based diets: An umbrella review. PLoS One 2024;19(5):e0300711.

(216) Baroni L, Rizzo G, Galchenko AV, Zavoli M, Serventi L, Battino M. Health Benefits of Vegetarian Diets: An Insight into the Main Topics. Foods 2024 Jul 29;13(15).

(217) Torres A, Quintanilla F, Barnafi E, Sanchez C, Acevedo F, Walbaum B, et al. Dietary Interventions for Cancer Prevention: An Update to ACS International Guidelines. Nutrients 2024 Aug 29;16(17).

(218) Almasi F, Nemati M, Aminianfar A. Dietary Recommendations for Glioma: A Mini-Review. Curr Nutr Rep 2024 Dec;13(4):966-71.

(219) Konieczna J, Chaplin A, Paz-Graniel I, Croker H, Becerra-Tomas N, Markozannes G, et al. Adulthood dietary and lifestyle patterns and risk of breast cancer: Global Cancer Update Programme (CUP Global) systematic literature review. Am J Clin Nutr 2025 Jan;121(1):14-31.

(220) Mandal S. Vegetarianism and risk of colorectal carcinoma: A systematic review. American Journal of Gastroenterology Conference: 80th Annual Scientific Meeting of the American College of Gastroenterology Honolulu, HI United States 110(Supplement 1) (pp S923), 2015;October.

(221) Davis B. Plant-based diets in the prevention and treatment of chronic disease. Complementary Medicine Research Conference: VegMed - Scientific Congress for Plant-based Nutrition and Medicine Berlin Germany 25(Supplement 1) (pp 9), 2018;01.

(222) Wynder EL, Onderdonk J, Mantel N. An epidemiological investigation of cancer of the bladder. Cancer 16(11) (pp 1388-1407), 1963;1963.

(223) Ritter MM, Richter WO. [Effects of a vegetarian life style on health]. Fortschr Med 1995 Jun 10;113(16):239-42.

(224) de Luis Roman D, Aller R, Castano O. [Vegetarian diets; effect on health]. Rev Clin Esp 2007 Mar;207(3):141-3.

(225) Fields H, Millstine D, Agrwal N. Just plants? Impact of a vegetarian diet on mortality. J Womens Health (Larchmt ) 2014 Nov;23(11):987-8.

Supplementary table 2. Cohort studies on vegetarian and vegan diets and total cancer

| Author, publication year, country/region | Study name | Period of recruitment, year of censoring, duration of follow-up | Number of participants, sex, age: number of cases | Dietary assessment method | Comparison | RR (95% CI) | Adjustment for confounding factors |
| --- | --- | --- | --- | --- | --- | --- | --- |
| Key TJ, 2014, United Kingdom | EPIC-Oxford Study and Oxford Vegetarian Study | 1980-1984 - 2010  1993-1999 - 2010,  14.9 years follow-up | 61647 men and women, age 20-89 years: 4998 cancer cases | FFQ, 45 items (OVS)  Validated FFQ, 130 food items (EPIC-Oxford) | Vegetarian/vegans vs. meat eater  Vegetarian/vegans vs. meat eater  Vegetarians vs. meat eaters  Vegetarians vs. meat eaters  Vegans vs. meat eaters  Vegans vs. meat eaters | 0.88 (0.82-0.95)  0.90 (0.83-0.96) + BMI  0.89 (0.83-0.96)  0.90 (0.84-0.97) + BMI  0.81 (0.66-0.98)  0.82 (0.68-1.00) + BMI | Age, sex, study/method of recruitment, smoking status and cigarettes/day, alcohol, physical activity |
| Watling C et al, 2022, United Kingdom | UK Biobank | 2006-2010 - 2021, 11.4 years follow-up | 472377 men and women, age 40-70 years: 54961 cancer cases | Validated FFQ | Vegetarian vs. regular meat eater  Vegetarian vs. regular meat eater | 0.86 (0.80-0.93)  0.88 (0.82-0.96) + BMI | Age, sex, region, height, physical activity, Townsend deprivation index, education, employment status, smoking status, alcohol, ethnicity, diabetes, menopausal status, HRT |
| Fraser G et al, 2025, USA | Adventist Health Study 2 | 2002-2007 - 2010-2013, 7.91 years follow-up | 79468 men and women, age ≥30 years: 5564 cancer cases | Validated FFQ, >200 food items | All vegetarians vs. meat eaters  All vegetarians vs. meat eaters  Vegetarian vs. meat eater  Vegetarian vs. meat eater  Vegan vs. meat eater  Vegan vs. meat eater | 0.88 (0.83-0.93)  0.95 (0.89-1.00) +BMI  0.91 (0.85-0.97)  0.94 (0.89-1.01) +BMI  0.76 (0.68-0.85)  0.82 (0.73-0.92) +BMI | Age, sex, race, education, height, exercise, FH - BC, HRT, age at menopause, menopausal status, parity, OC use, mammography, PSA test, digital rectal examination, colonoscopy, fecal occult blood testing |

Abbreviations: +BMI, also adjusted for body mass index; FFQ, food frequency questionnaire; FH - BC, family history of breast cancer; HRT, hormone replacement therapy; OC use, oral contraceptive use; PSA, prostate-specific antigen.

Supplementary table 3. Cohort studies on vegetarian and vegan diets and upper aerodigestive tract cancer

| Author, publication year, country/region | Study name | Period of recruitment, year of censoring, duration of follow-up | Number of participants, sex, age: number of cases | Dietary assessment method | Comparison | RR (95% CI) | Adjustment for confounding factors |
| --- | --- | --- | --- | --- | --- | --- | --- |
| Key TJ, 2014, United Kingdom | EPIC-Oxford Study and Oxford Vegetarian Study | 1980-1984 - 2010  1993-1999 - 2010,  14.9 years follow-up | 61647 men and women, age 20-89 years: 134 upper gastrointestinal tract cancer cases | FFQ, 45 items (OVS)  Validated FFQ, 130 food items (EPIC-Oxford) | Vegetarian/vegans vs. meat eater  Vegetarian/vegans vs. meat eater | 0.94 (0.62-1.43)  0.93 (0.61-1.43) +BMI | Age, sex, study/method of recruitment, smoking status and cigarettes/day, alcohol, physical activity |
| Parra-Soto S, 2022, United Kingdom | UK Biobank | 2006-2010 - 2020, 10.6 years follow-up | 409110 men and women, age 37-73 years: 848 head and neck cancer cases | Validated FFQ | Vegetarian vs. meat eater  Vegetarian vs. meat eater | 1.00 (0.56-1.77)  0.94 (0.53-1.66) +BMI | Age, sex, deprivation, ethnicity, smoking, alcohol, total physical activity, fruits, vegetables, comorbidity |
| Fraser G et al, 2025, USA | Adventist Health Study 2 | 2002-2007 - 2010-2013, 7.91 years follow-up | 79468 men and women, age ≥30 years: 68 mouth, pharynx and larynx cancer cases | Validated FFQ, >200 food items | All vegetarians vs. meat eaters  All vegetarians vs. meat eaters  Vegetarian vs. meat eater  Vegetarian vs. meat eater  Vegan vs. meat eater  Vegan vs. meat eater | 1.05 (0.63-1.76)  1.00 (0.59-1.69) +BMI  0.95 (0.52-1.75)  0.91 (0.49-1.68) +BMI  1.22 (0.51-2.94)  1.11 (0.45-2.73) +BMI | Age, sex, race, education, cigarette smoking (status, cessation duration) |

Abbreviations: +BMI, also adjusted for body mass index; FFQ, food frequency questionnaire.

Supplementary table 4. Cohort studies on vegetarian and vegan diets and stomach cancer

| Author, publication year, country/region | Study name | Period of recruitment, year of censoring, duration of follow-up | Number of participants, sex, age: number of cases | Dietary assessment method | Comparison | RR (95% CI) | Adjustment for confounding factors |
| --- | --- | --- | --- | --- | --- | --- | --- |
| Key TJ, 2014, United Kingdom | EPIC-Oxford Study and Oxford Vegetarian Study | 1980-1984 - 2010  1993-1999 - 2010,  14.9 years follow-up | 61647 men and women, age 20-89 years: 70 stomach cancer cases | FFQ, 45 items (OVS)  Validated FFQ, 130 food items (EPIC-Oxford) | Vegetarian/vegans vs. meat eater  Vegetarian/vegans vs. meat eater | 0.37 (0.19-0.69)  0.38 (0.20-0.71) +BMI | Age, sex, study/method of recruitment, smoking status and cigarettes/day, alcohol, physical activity |
| Parra-Soto S, 2022, United Kingdom | UK Biobank | 2006-2010 - 2020, 10.6 years follow-up | 409110 men and women, age 37-73 years: 801 stomach cancer cases | Validated FFQ | Vegetarian vs. meat eater  Vegetarian vs. meat eater | 0.87 (0.43-1.75)  0.94 (0.47-1.90) +BMI | Age, sex, deprivation, ethnicity, smoking, alcohol, total physical activity, fruits, vegetables, comorbidity |
| Fraser G et al, 2025, USA | Adventist Health Study 2 | 2002-2007 - 2010-2013, 7.91 years follow-up | 79468 men and women, age ≥30 years: 64 stomach cancer cases | Validated FFQ, >200 food items | All vegetarians vs. meat eaters  All vegetarians vs. meat eaters  Vegetarian vs. meat eater  Vegetarian vs. meat eater  Vegan vs. meat eater  Vegan vs. meat eater | 0.55 (0.32-0.93)  0.59 (0.34-1.01) +BMI  0.52 (0.28-0.98)  0.55 (0.29-1.05) +BMI  0.86 (0.36-2.04)  0.95 (0.39-2.31) +BMI | Age, sex, race, education, cigarette smoking (status, cessation duration) |

Abbreviations: +BMI, also adjusted for body mass index; FFQ, food frequency questionnaire.

Supplementary table 5. Cohort studies on vegetarian and vegan diets and colorectal cancer

| Author, publication year, country/region | Study name | Period of recruitment, year of censoring, duration of follow-up | Number of participants, sex, age: number of cases | Dietary assessment method | Comparison | | RR (95% CI) | Adjustment for confounding factors |
| --- | --- | --- | --- | --- | --- | --- | --- | --- |
| Singh PN et al, 1998, USA | Adventist Health Study | 1976-1982, 6 years follow-up | 32051 men and women, age ≥25 years: 135 CC cases | FFQ, 55 food items | Total meat (red meat, poultry, fish) | Never  >0-<1/week  ≥1/week | 1.00  1.50 (0.92-2.45)  1.85 (1.16-2.87) | Age, sex, BMI, physical activity, parental history of CC, smoking status, alcohol, aspirin use |
| Key TJ, 2014, United Kingdom | EPIC-Oxford Study and Oxford Vegetarian Study | 1980-1984 - 2010  1993-1999 - 2010,  14.9 years follow-up | 61647 men and women, age 20-89 years: 579 CRC cases  364 CC cases  215 RC cases | FFQ, 45 items (OVS)  Validated FFQ, 130 food items (EPIC-Oxford) | Vegetarian/vegans vs. meat eater, CRC  Vegetarian/vegans vs. meat eater  Vegetarian/vegans vs. meat eater, CC  Vegetarian/vegans vs. meat eater  Vegetarian/vegans vs. meat eater, RC  Vegetarian/vegans vs. meat eater  Vegetarians vs. meat eaters, CRC  Vegetarians vs. meat eaters  Vegans vs. meat eaters, CRC  Vegans vs. meat eaters | | 1.03 (0.84-1.26)  1.04 (0.84-1.28) +BMI  0.99 (0.76-1.30)  1.01 (0.77-1.33) +BMI  1.10 (0.80-1.50)  1.08 (0.79-1.48) +BMI  1.00 (0.81-1.24)  1.01 (0.81-1.25) +BMI  1.29 (0.81-2.07)  1.31 (0.82-2.11) +BMI | Age, sex, study/method of recruitment, smoking status and cigarettes/day, alcohol, physical activity |
| Gilsing AMJ et al, 2015, Netherlands | Netherlands Cohort Study - Meat Investigation Cohort | 1986 - NA, 20.3 years follow-up | 10210 men and women, age 55-69 years: 437 CRC cases  307 CC cases  92 RC cases | FFQ, 150 food items | Vegetarian vs. non-vegetarian, CRC  Vegetarian vs. non-vegetarian, CC  Vegetarian vs. non-vegetarian, RC | | 0.89 (0.58-1.39)  1.06 (0.65-1.70)  0.22 (0.03-1.63) | Age, sex, total energy, cigarette smoking status, alcohol, BMI, non-occupational physical activity, education |
| de Jauregui DRF et al, 2018, United Kingdom | UK Women's Cohort Study | 1995-1998 - NA, 17.2 years follow-up | 32147 women, age 35-69 years: 462 CRC cases  335 CC cases  172 PC cases  119 DC cases  152 RC cases | Validated FFQ, 217 food items | Vegetarian vs. red meat eater, CRC  Vegetarian vs. red meat eater, CC  Vegetarian vs. red meat eater, PCC  Vegetarian vs. red meat eater, DCC  Vegetarian vs. red meat eater, RC | | 0.80 (0.58-1.11)  0.71 (0.47-1.08)  0.73 (0.39-1.37)  0.74 (0.40-1.36)  0.91 (0.55-1.52) | Age, BMI, energy intake, physical activity, smoking status, family history of colorectal cancer in first degree relative, socio-economic status |
| Watling C et al, 2022, United Kingdom | UK Biobank | 2006-2010 - 2021, 11.4 years follow-up | 472377 men and women, age 40-70 years: 5882 CRC cases | Validated FFQ | Vegetarian vs. regular meat eater  Vegetarian vs. regular meat eater | | 0.78 (0.61-1.01)  0.81 (0.63-1.04) +BMI | Age, sex, region, height, physical activity, Townsend deprivation index, education, employment status, smoking status, alcohol, ethnicity, diabetes, menopausal status, HRT, NSAIDS |
| Parra-Soto S, 2022, United Kingdom | UK Biobank | 2006-2010 - 2020, 10.6 years follow-up | 409110 men and women, age 37-73 years:  3450 CC cases  1733 PCC cases  1494 DCC cases  2122 RC cases | Validated FFQ | Vegetarian/vegan vs. meat eater, CC  Vegetarian/vegan vs. meat eater, CC  Vegetarian/vegan vs. meat eater, PCC  Vegetarian/vegan vs. meat eater, PCC  Vegetarian/vegan vs. meat eater, DCC  Vegetarian/vegan vs. meat eater, DCC  Vegetarian/vegan vs. meat eater, RC  Vegetarian/vegan vs. meat eater, RC | | 0.66 (0.46-0.96)  0.69 (0.48-0.99) +BMI  0.41 (0.21-0.79)  0.43 (0.22-0.82) +BMI  0.90 (0.56-1.45)  0.94 (0.58-1.51) +BMI  0.70 (0.44-1.11)  0.72 (0.45-1.15) +BMI | Age, sex, deprivation, ethnicity, smoking, alcohol, total physical activity, fruits, vegetables, comorbidity |
| Fraser et al, 2025, USA | Adventist Health Study 2 | 2002-2007 - 2010-2013, 7.91 years follow-up | 79468 men and women, age ≥30 years: 532 CRC cases  423 CC cases  109 RC cases | Validated FFQ, >200 food items | All vegetarians vs. meat eaters, CRC  All vegetarians vs. meat eaters, CRC  All vegetarians vs. meat eaters, CC  All vegetarians vs. meat eaters, CC  All vegetarians vs. meat eaters, RC  All vegetarians vs. meat eaters, RC  Vegetarian vs. meat eater, CRC  Vegetarian vs. meat eater, CRC  Vegetarian vs. meat eater, CC  Vegetarian vs. meat eater, CC  Vegetarian vs. meat eater, RC  Vegetarian vs. meat eater, RC  Vegan vs. meat eater, CRC  Vegan vs. meat eater, CRC  Vegan vs. meat eater, CC  Vegan vs. meat eater, CC  Vegan vs. meat eater, RC  Vegan vs. meat eater, RC | | 0.79 (0.66-0.95)  0.87 (0.72-1.04) +BMI  0.80 (0.65-0.98)  0.86 (0.57-1.30) +BMI  0.80 (0.53-1.20)  0.87 (0.71-1.07) +BMI  0.83 (0.68-1.02)  0.88 (0.72-1.09) +BMI  0.85 (0.67-1.06)  0.90 (0.71-1.13) +BMI  0.80 (0.51-1.27)  0.85 (0.53-1.35) +BMI  0.88 (0.64-1.22)  0.98 (0.71-1.36) +BMI  0.82 (0.56-1.19)  0.91 (0.63-1.33) +BMI  1.13 (0.60-2.13)  1.26 (0.66-2.40) +BMI | Age, sex, race, education, height, physical activity, recent aspirin use, colonoscopy, fecal occult blood testing |

Abbreviations: +BMI, also adjusted for body mass index; CC, colon cancer; CRC, colorectal cancer; DCC, distal colon cancer; FFQ, food frequency questionnaire; HRT, hormone replacement therapy; PCC, proximal colon cancer, RC, rectal cancer.

Supplementary table 6. Cohort studies on vegetarian and vegan diets and pancreatic cancer

| Author, publication year, country/region | Study name | Period of recruitment, year of censoring, duration of follow-up | Number of participants, sex, age: number of cases | Dietary assessment method | Comparison | RR (95% CI) | Adjustment for confounding factors |
| --- | --- | --- | --- | --- | --- | --- | --- |
| Key TJ, 2014, United Kingdom | EPIC-Oxford Study and Oxford Vegetarian Study | 1980-1984 - 2010  1993-1999 - 2010,  14.9 years follow-up | 61647 men and women, age 20-89 years: 112 pancreatic cancer cases | FFQ, 45 items (OVS)  Validated FFQ, 130 food items (EPIC-Oxford) | Vegetarian/vegan vs. meat eater  Vegetarian/vegan vs. meat eater | 0.73 (0.44-1.21)  0.70 (0.42-1.17) +BMI | Age, sex, study/method of recruitment, smoking status and cigarettes/day, alcohol, physical activity |
| Parra-Soto S, 2022, United Kingdom | UK Biobank | 2006-2010 - 2020, 10.6 years follow-up | 409110 men and women, age 37-73 years: 1169 pancreatic cancer | Validated FFQ | Vegetarian/vegan vs. meat eater  Vegetarian/vegan vs. meat eater | 0.63 (0.33-1.21)  0.65 (0.34-1.26) +BMI | Age, sex, deprivation, ethnicity, smoking, alcohol, total physical activity, fruits, vegetables, comorbidity |
| Shyam S et al, 2023, United Kingdom | UK Women's Cohort Study | 1995-1998 - NA,  18.5 years follow-up | 35365 women, age 35-69 years: 136 pancreatic cancer cases | Validated FFQ, 217 food items | Omnivore vs. vegetarian/vegan | 1.13 (0.73-1.76) | Age, smoking, education, physical activity |
| Fraser G et al, 2025, USA | Adventist Health Study 2 | 2002-2007 - 2010-2013, 7.91 years follow-up | 79468 men and women, age ≥30 years: 164 pancreatic cancer cases | Validated FFQ, >200 food items | All vegetarians vs. meat eaters  All vegetarians vs. meat eaters  Vegetarian vs. meat eater  Vegetarian vs. meat eater  Vegan vs. meat eater  Vegan vs. meat eater | 0.76 (0.55-1.06)  0.84 (0.61-1.18) +BMI  0.67 (0.45-0.98)  0.72 (0.49-1.06) +BMI  0.60 (0.30-1.18)  0.69 (0.35-1.38) +BMI | Age, sex, race, education, height, cigarette smoking (status, cessation duration) |

Abbreviations: +BMI, also adjusted for body mass index; FFQ, food frequency questionnaire.

Supplementary table 7. Cohort studies on vegetarian and vegan diets and lung cancer

| Author, publication year, country/region | Study name | Period of recruitment, year of censoring, duration of follow-up | Number of participants, sex, age: number of cases | Dietary assessment method | Comparison | | RR (95% CI) | Adjustment for confounding factors |
| --- | --- | --- | --- | --- | --- | --- | --- | --- |
| Fraser G et al, 1991, USA | Adventist Health Study | 1976 - 1982, 6 years follow-up | 34198 men and women, age ≥25 years: 61 lung cancer cases | FFQ, 51 food items | Meat, poultry, fish | Never  ≤2/week  >2/week | 1.00  1.86 (0.82-4.23)  1.31 (0.52-3.28) | Age, sex, smoking history |
| Key TJ, 2014, United Kingdom | EPIC-Oxford Study and Oxford Vegetarian Study | 1980-1984 - 2010  1993-1999 - 2010,  14.9 years follow-up | 61647 men and women, age 20-89 years: 236 lung cancer cases | FFQ, 45 items (OVS)  Validated FFQ, 130 food items (EPIC-Oxford) | Vegetarian/vegans vs. meat eater  Vegetarian/vegans vs. meat eater | | 1.16 (0.83-1.61)  1.09 (0.78-1.53) +BMI | Age, sex, study/method of recruitment, smoking status and cigarettes/day, alcohol, physical activity |
| Gilsing AMJ et al, 2016, Netherlands | Netherlands Cohort Study - Meat Investigation Cohort | 1986 - NA, 20.3 years follow-up | 9773 men and women, age 55-69 years: 279 lung cancer cases | FFQ, 150 food items | Vegetarian vs. non-vegetarian | | 0.86 (0.40-1.85) | Age, total energy, cigarette smoking status, cigarettes/day, duration of smoking, alcohol, BMI, non-occupational physical activity, education |
| Parra-Soto S, 2022, United Kingdom | UK Biobank | 2006-2010 - 2020, 10.6 years follow-up | 409110 men and women, age 37-73 years: 3407 lung cancer | Validated FFQ | Vegetarian/vegan vs. meat eater  Vegetarian/vegan vs. meat eater | | 0.78 (0.53-1.14)  0.76 (0.52-1.11) +BMI | Age, sex, deprivation, ethnicity, smoking, alcohol, total physical activity, fruits, vegetables, comorbidity |
| Fraser G et al, 2025, USA | Adventist Health Study 2 | 2002-2007 - 2010-2013, 7.91 years follow-up | 79468 men and women, age ≥30 years: 257 lung cancer cases | Validated FFQ, >200 food items | All vegetarians vs. meat eaters  All vegetarians vs. meat eaters  Vegetarian vs. meat eater  Vegetarian vs. meat eater  Vegan vs. meat eater  Vegan vs. meat eater | | 0.77 (0.58-1.01)  0.74 (0.56-0.99) +BMI  0.82 (0.60-1.13)  0.80 (0.58-1.10) +BMI  0.61 (0.34-1.11)  0.58 (0.32-1.06) +BMI | Age, sex, race, education, physical activity, cigarette smoking (status, cessation duration) |

Abbreviations: +BMI, also adjusted for body mass index; FFQ, food frequency questionnaire.

Supplementary table 8. Cohort studies on vegetarian and vegan diets and melanoma

| Author, publication year, country/region | Study name | Period of recruitment, year of censoring, duration of follow-up | Number of participants, sex, age: number of cases | Dietary assessment method | Comparison | RR (95% CI) | Adjustment for confounding factors |
| --- | --- | --- | --- | --- | --- | --- | --- |
| Key TJ, 2014, United Kingdom | EPIC-Oxford Study and Oxford Vegetarian Study | 1980-1984 - 2010  1993-1999 - 2010,  14.9 years follow-up | 61647 men and women, age 20-89 years: 299 melanoma cases | FFQ, 45 items (OVS)  Validated FFQ, 130 food items (EPIC-Oxford) | Vegetarian/vegan vs. meat eater  Vegetarian/vegan vs. meat eater | 0.79 (0.59-1.07)  0.78 (0.57-1.05) +BMI | Age, sex, study/method of recruitment, smoking status and cigarettes/day, alcohol, physical activity |
| Parra-Soto S, 2022, United Kingdom | UK Biobank | 2006-2010 - 2020, 10.6 years follow-up | 409110 men and women, age 37-73 years: 2043 melanoma cases | Validated FFQ | Vegetarian/vegan vs. meat eater  Vegetarian/vegan vs. meat eater | 0.67 (0.43-1.06)  0.68 (0.43-1.07) +BMI | Age, sex, deprivation, ethnicity, smoking, alcohol, total physical activity, fruits, vegetables, comorbidity |
| Fraser G et al, 2025, USA | Adventist Health Study 2 | 2002-2007 - 2010-2013, 7.91 years follow-up | 79468 men and women, age ≥30 years: 233 melanoma cases | Validated FFQ, >200 food items | All vegetarians vs. meat eaters  All vegetarians vs. meat eaters  Vegetarian vs. meat eater  Vegetarian vs. meat eater  Vegan vs. meat eater  Vegan vs. meat eater | 0.83 (0.63-1.08)  0.82 (0.62-1.09) +BMI  0.86 (0.64-1.16)  0.84 (0.62-1.14) +BMI  0.75 (0.45-1.28)  0.73 (0.43-1.25) +BMI | Age, sex, race, education, alcohol |

Abbreviations: +BMI, also adjusted for body mass index; FFQ, food frequency questionnaire.

Supplementary table 9. Cohort studies on vegetarian and vegan diets and breast cancer

| Author, publication year, country/region | Study name | Period of recruitment, year of censoring, duration of follow-up | Number of participants, sex, age: number of cases | Dietary assessment method | Comparison | RR (95% CI) | Adjustment for confounding factors |
| --- | --- | --- | --- | --- | --- | --- | --- |
| Mills PK et al, 1989, USA | Adventist Health Study | 1976 - 1982, 6 years follow-up | 20341 women, age ≥25 years: 215 breast cancer cases | FFQ | Lacto-ovo vegetarian vs. omnivore  Pure vegetarian vs. omnivore | 0.72 (0.47-1.09)  0.78 (0.56-1.07) | Age, age at first live birth, age at menarche, menopausal status, history of benign breast disease, maternal history of breast cancer, education, BMI |
| Cade JE et al, 2010, United Kingdom | UK Women's Cohort Study | 1995-1998 - 2006, 9 years follow-up | 33725 women, age 35-69 years: 330/453 pre/post-menopausal breast cancer cases | Validated FFQ, 217 food items | Vegetarian vs. red meat eater, all  Vegetarian vs. red meat eater, premenopausal  Vegetarian vs. red meat eater, postmenopausal | 0.88 (0.69-1.11)  0.92 (0.67-1.24)  0.85 (0.58-1.25) | Age, energy intake, menopausal status, calorie adjusted fat intake, BMI, physical activity, OC use, HRT, smoking status, parity, age at menarche, ethanol, total days breastfeeding, socioeconomic class, education |
| Key TJ, 2014, United Kingdom | EPIC-Oxford Study and Oxford Vegetarian Study | 1980-1984 - 2010  1993-1999 - 2010,  14.9 years follow-up | 46053 women, age 20-89 years: 1452 breast cancer cases | FFQ, 45 items (OVS)  Validated FFQ, 130 food items (EPIC-Oxford) | Vegetarian/vegans vs. meat eater  Vegetarian/vegans vs. meat eater  Vegetarians vs. meat eaters  Vegetarians vs. meat eaters  Vegans vs. meat eaters  Vegans vs. meat eaters | 0.93 (0.82-1.07)  0.96 (0.83-1.10) + BMI  0.94 (0.82-1.08)  0.96 (0.84-1.10) +BMI  0.87 (0.59-1.28)  0.91 (0.61-1.34) +BMI | Age, study/method of recruitment, smoking status and cigarettes/day, alcohol, physical activity, parity, OC use |
| Gilsing AMJ et al, 2016, Netherlands | Netherlands Cohort Study - Meat Investigation Cohort | 1986 - , 20.3 years follow-up | 5218 post-menopausal women, age 55-69 years: 312 breast cancer cases | FFQ, 150 food items | Vegetarian vs. non-vegetarian | 0.70 (0.43-1.14) | Age, total energy, cigarette smoking status, alcohol, BMI, non-occupational physical activity, education, family history of breast cancer, age at menarche, age at menopause, age at first child, HRT, OC use, number of children |
| Watling C et al, 2022, United Kingdom | UK Biobank | 2006-2010 - 2021, 11.4 years follow-up | 179098 post-menopausal women, age 40-70 years: 7537 breast cancer cases | Validated FFQ | Vegetarian vs. regular meat eater  Vegetarian vs. regular meat eater | 0.82 (0.68-0.99)  0.87 (0.72-1.05) +BMI | Age, region, height, physical activity, Townsend deprivation index, education, employment status, smoking status, alcohol, ethnicity, diabetes, menopausal status, HRT, age at menarche, age at first birth, parity |
| Parra-Soto S, 2022, United Kingdom | UK Biobank | 2006-2010 - 2020, 10.6 years follow-up | 218391 women, age 37-73 years: 6895 breast cancer cases  1776 premenopausal breast cancer cases | Validated FFQ | Vegetarian/vegan vs. meat eater, all  Vegetarian/vegan vs. meat eater, all  Vegetarian/vegan vs. meat eater, premenopausal  Vegetarian/vegan vs. meat eater, premenopausal | 0.93 (0.78-1.10)  0.95 (0.80-1.13) +BMI  1.01 (0.76-1.33)  1.00 (0.75-1.33) +BMI | Age, deprivation, ethnicity, smoking, alcohol, total physical activity, fruits, vegetables, comorbidity |
| Fraser G et al, 2025, USA | Adventist Health Study 2 | 2002-2007 - 2010-2013, 7.91 years follow-up | 51654 women, age ≥30 years: 1027 breast cancer cases | Validated FFQ, >200 food items | All vegetarians vs. meat eaters  All vegetarians vs. meat eaters, age 65  All vegetarians vs. meat eaters, age 85  Vegetarian vs. meat eater, age 65  Vegetarian vs. meat eater, age 85  Vegetarian vs. meat eater, age 65  Vegetarian vs. meat eater, age 85  Vegan vs. meat eater, age 65  Vegan vs. meat eater, age 85  Vegan vs. meat eater, age 65  Vegan vs. meat eater, age 85 | 0.99 (0.87-1.13)  1.06 (0.93-1.21) +BMI  0.96 (0.77-1.33) +BMI  1.08 (0.93-1.25)  0.99 (0.78-1.27)  1.11 (0.96-1.29) +BMI  1.02 (0.80-1.30) +BMI  0.69 (0.51-0.92)  0.74 (0.46-1.19)  0.73 (0.54-0.98) +BMI  0.78 (0.48-1.25) +BMI | Age, race, education, menopausal status, age at menarche, HRT, OC use, FH - BC, physical activity, breastfeeding, height |

Abbreviations: +BMI, also adjusted for body mass index; FFQ, food frequency questionnaire; FH - BC, family history of breast cancer; HRT, hormone replacement therapy; OC use, oral contraceptive use.

Supplementary table 10. Cohort studies on vegetarian and vegan diets and endometrial cancer

| Author, publication year, country/region | Study name | Period of recruitment, year of censoring, duration of follow-up | Number of participants, sex, age: number of cases | Dietary assessment method | Comparison | RR (95% CI) | Adjustment for confounding factors |
| --- | --- | --- | --- | --- | --- | --- | --- |
| Fraser GE, 1999, USA | Adventist Health Study | 1976 - 1982, 6 years follow-up | 20341 women, age ≥25 years: 116 uterine cancer cases | FFQ, 51 food items | Non-vegetarians vs. vegetarians | 1.17 (0.81-1.71) | Age |
| Key TJ, 2014, United Kingdom | EPIC-Oxford Study and Oxford Vegetarian Study | 1980-1984 - 2010  1993-1999 - 2010,  14.9 years follow-up | 46053 women, age 20-89 years: 177 endometrial cancer | FFQ, 45 items (OVS)  Validated FFQ, 130 food items (EPIC-Oxford) | Vegetarian/vegans vs. meat eater  Vegetarian/vegans vs. meat eater | 0.91 (0.62-1.33)  0.99 (0.67-1.45) +BMI | Age, study/method of recruitment, smoking status and cigarettes/day, alcohol, physical activity, parity, oral contraceptive use |
| Parra-Soto S, 2022, United Kingdom | UK Biobank | 2006-2010 - 2020, 10.6 years follow-up | 218391 women, age 37-73 years: 1202 uterine cancer cases | Validated FFQ | Vegetarian/vegan vs. meat eater  Vegetarian/vegan vs. meat eater | 0.97 (0.64-1.46)  1.15 (0.76-1.74) +BMI | Age, deprivation, ethnicity, smoking, alcohol, total physical activity, fruits, vegetables, comorbidity |
| Fraser G et al, 2025, USA | Adventist Health Study 2 | 2002-2007 - 2010-2013, 7.91 years follow-up | 51654 women, age ≥30 years: 272 uterine cancer cases | Validated FFQ, >200 food items | All vegetarians vs. meat eaters  All vegetarians vs. meat eaters  Vegetarian vs. meat eater  Vegetarian vs. meat eater  Vegan vs. meat eater  Vegan vs. meat eater | 0.91 (0.71-1.16)  1.22 (0.94-1.57) +BMI  0.85 (0.64-1.14)  1.09 (0.81-1.46) +BMI  1.02 (0.66-1.59)  1.59 (1.01-2.49) +BMI | Age, race, education, height, physical activity |

Abbreviations: +BMI, also adjusted for body mass index; FFQ, food frequency questionnaire.

Supplementary table 11. Cohort studies on vegetarian and vegan diets and ovarian cancer

| Author, publication year, country/region | Study name | Period of recruitment, year of censoring, duration of follow-up | Number of participants, sex, age: number of cases | Dietary assessment method | Comparison | | RR (95% CI) | Adjustment for confounding factors |
| --- | --- | --- | --- | --- | --- | --- | --- | --- |
| Kiani F et al, 2006, USA | Adventist Health Study | 1976 - 1992, 16 years follow-up | 13281 women, age ≥25 years: 71 ephitelial ovarian cancer cases | FFQ, 55 food items | Total meat index (red meat, poultry, fish), all cases  Total meat index (red meat, poultry, fish), postmenopausal cases | Never  >0-<1/week  ≥1/week  Never  >0-<1/week  ≥1/week | 1.00  0.97 (0.43-2.19)  1.69 (0.88-3.24)  1.00  1.03 (0.39-2.71)  2.42 (1.16-5.08) | Age, parity, BMI, age at menopause, HRT in postmenopausal women |
| Key TJ, 2014, United Kingdom | EPIC-Oxford Study and Oxford Vegetarian Study | 1980-1984 - 2010  1993-1999 - 2010,  14.9 years follow-up | 46053 women, age 20-89 years: 221 ovarian cancer cases | FFQ, 45 items (OVS)  Validated FFQ, 130 food items (EPIC-Oxford) | Vegetarian/vegans vs. meat eater  Vegetarian/vegans vs. meat eater | | 0.86 (0.61-1.20)  0.87 (0.61-1.22) +BMI | Age, study/method of recruitment, smoking status and cigarettes/day, alcohol, physical activity, parity, OC use |
| Parra-Soto S, 2022, United Kingdom | UK Biobank | 2006-2010 - 2020, 10.6 years follow-up | 218391 women, age 37-73 years: 935 ovarian cancer cases | Validated FFQ | Vegetarian/vegan vs. meat eater  Vegetarian/vegan vs. meat eater | | 1.13 (0.72-1.79)  1.14 (0.72-1.80) +BMI | Age, deprivation, ethnicity, smoking, alcohol, total physical activity, fruits, vegetables, comorbidity, BMI |
| Fraser G et al, 2025, USA | Adventist Health Study 2 | 2002-2007 - 2010-2013, 7.91 years follow-up | 51654 women, age ≥30 years: 127 ovarian cancer cases | Validated FFQ, >200 food items | All vegetarians vs. meat eaters  All vegetarians vs. meat eaters  Vegetarian vs. meat eater  Vegetarian vs. meat eater  Vegan vs. meat eater  Vegan vs. meat eater | | 0.70 (0.48-1.01)  0.74 (0.51-1.09) +BMI  0.79 (0.52-1.18)  0.82 (0.54-1.25) +BMI  0.57 (0.26-1.24)  0.62 (0.28-1.36) +BMI | Age, race, education, height, breastfeeding months, OC use, HRT |

Abbreviations: +BMI, also adjusted for body mass index; FFQ, food frequency questionnaire; HRT, hormone replacement therapy; OC use, oral contraceptive use.

Supplementary table 12. Cohort studies on vegetarian and vegan diets and prostate cancer

| Author, publication year, country/region | Study name | Period of recruitment, year of censoring, duration of follow-up | Number of participants, sex, age: number of cases | Dietary assessment method | Comparison | | RR (95% CI) | Adjustment for confounding factors |
| --- | --- | --- | --- | --- | --- | --- | --- | --- |
| Mills PK et al, 1989, USA | Adventist Health Study | 1976-1982, 6 years follow-up | 14000 men, age ≥25 years: 152 prostate cancer cases | FFQ | Meat, poultry, fish | Never  <daily  ≥daily | 1.00  1.15 (0.79-1.69)  1.41 (0.79-2.51) | Age, education, fish, beans, legumes/peas, citrus fruit, dry fruit, nuts, tomatoes |
| Key TJ, 2014, United Kingdom | EPIC-Oxford Study and Oxford Vegetarian Study | 1980-1984 - 2010  1993-1999 - 2010,  14.9 years follow-up | 15594 men, age 20-89 years: 457 prostate cancer cases | FFQ, 45 items (OVS)  Validated FFQ, 130 food items (EPIC-Oxford) | Vegetarian/vegans vs. meat eater  Vegetarian/vegans vs. meat eater  Vegetarians vs. meat eaters  Vegetarians vs. meat eaters  Vegans vs. meat eaters  Vegans vs. meat eaters | | 0.84 (0.66-1.07)  0.83 (0.64-1.06) +BMI  0.87 (0.68-1.12)  0.86 (0.66-1.11) +BMI  0.62 (0.31-1.22)  0.61 (0.31-1.20) +BMI | Age, study/method of recruitment, smoking status and cigarettes/day, alcohol, physical activity |
| Gilsing AMJ et al, 2016, Netherlands | Netherlands Cohort Study - Meat Investigation Cohort | 1986 - NA, 20.3 years follow-up | 4864 men, age 55-69 years: 399 prostate cancer cases  136 advanced prostate cancer cases | FFQ, 150 food items | Vegetarian vs. non-vegetarian, all prostate cancer  Vegetarian vs. non-vegetarian, advanced prostate cancer | | 1.09 (0.68-1.76)  1.25 (0.57-2.74) | Age, total energy, cigarette smoking status, alcohol, BMI, non-occupational physical activity, education, family history of prostate cancer |
| Watling C et al, 2022, United Kingdom | UK Biobank | 2006-2010 - 2021, 11.4 years follow-up | 217977 men, age 40-70 years: 9508 prostate cancer cases | Validated FFQ | Vegetarian vs. regular meat eater  Vegetarian vs. regular meat eater | | 0.69 (0.54-0.89)  0.69 (0.54-0.89) + BMI | Age, region, height, physical activity, Townsend deprivation index, education, employment status, smoking status, alcohol, ethnicity, diabetes, marital status |
| Fraser G et al, 2025, USA | Adventist Health Study 2 | 2002-2007 - 2010-2013, 7.91 years follow-up | 27814 men, age ≥30 years: 1219 prostate cancer cases | Validated FFQ, >200 food items | All vegetarians vs. meat eaters  All vegetarians vs. meat eaters  Vegetarian vs. meat eater, age 65  Vegetarian vs. meat eater, age 65  Vegetarian vs. meat eater, age 85  Vegetarian vs. meat eater, age 85  Vegan vs. meat eater, age 65  Vegan vs. meat eater, age 65  Vegan vs. meat eater, age 85  Vegan vs. meat eater, age 85 | | 0.97 (0.86-1.10)  1.00 (0.88-1.13) +BMI  1.01 (0.87-1.18)  1.02 (0.87-1.19) +BMI  0.97 (0.76-1.25)  0.98 (0.76-1.26) +BMI  0.57 (0.41-0.80)  0.58 (0.41-0.81) +BMI  1.08 (0.71-1.65)  1.09 (0.71-1.67) +BMI | Age, race, education, height |

Abbreviations: +BMI, also adjusted for body mass index; FFQ, food frequency questionnaire.

Supplementary table 13. Cohort studies on vegetarian and vegan diets and kidney cancer

| Author, publication year, country/region | Study name | Period of recruitment, year of censoring, duration of follow-up | Number of participants, sex, age: number of cases | Dietary assessment method | Comparison | RR (95% CI) | Adjustment for confounding factors |
| --- | --- | --- | --- | --- | --- | --- | --- |
| Key TJ, 2014, United Kingdom | EPIC-Oxford Study and Oxford Vegetarian Study | 1980-1984 - 2010  1993-1999 - 2010,  14.9 years follow-up | 61647 men and women, age 20-89 years: 80 kidney cancer cases | FFQ, 45 items (OVS)  Validated FFQ, 130 food items (EPIC-Oxford) | Vegetarian/vegan vs. meat eater  Vegetarian/vegan vs. meat eater | 0.90 (0.51-1.60)  1.02 (0.58-1.78) +BMI | Age, sex, study/method of recruitment, smoking status and cigarettes/day, alcohol, physical activity |
| Parra-Soto S, 2022, United Kingdom | UK Biobank | 2006-2010 - 2020, 10.6 years follow-up | 409110 men and women, age 37-73 years: 1268 kidney cancer cases | Validated FFQ | Vegetarian/vegan vs. meat eater  Vegetarian/vegan vs. meat eater | 0.81 (0.46-1.44)  0.88 (0.50-1.56) +BMI | Age, sex, deprivation, ethnicity, smoking, alcohol, total physical activity, fruits, vegetables, comorbidity |
| Fraser G et al, 2025, USA | Adventist Health Study 2 | 2002-2007 - 2010-2013, 7.91 years follow-up | 79468 men and women, age ≥30 years: 135 renal cancer cases | Validated FFQ, >200 food items | All vegetarians vs. meat eaters  All vegetarians vs. meat eaters  Vegetarian vs. meat eater  Vegetarian vs. meat eater  Vegan vs. meat eater  Vegan vs. meat eater | 0.84 (0.58-1.21)  1.00 (0.68-1.45) +BMI  0.85 (0.56-1.30)  0.98 (0.64-1.51) +BMI  0.90 (0.46-1.76)  1.18 (0.60-2.33) +BMI | Age, sex, race, education, cigarette smoking (status and cessation), height |

Abbreviations: +BMI, also adjusted for body mass index; FFQ, food frequency questionnaire.

Supplementary table 14. Cohort studies on vegetarian and vegan diets and bladder cancer

| Author, publication year, country/region | Study name | Period of recruitment, year of censoring, duration of follow-up | Number of participants, sex, age: number of cases | Dietary assessment method | Comparison | | RR (95% CI) | Adjustment for confounding factors |
| --- | --- | --- | --- | --- | --- | --- | --- | --- |
| Mills PK et al, 1991, USA | Adventist Health Study | 1976 - 1982, 6 years follow-up | 34198 men and women, age ≥25 years: 52 bladder cancer cases | FFQ, 51 food items | Meat, poultry, fish  Meat, poultry, fish, never smokers  Meat, poultry, fish, ever smokers | None  <3/week  ≥3/week  None  <3/week  ≥3/week  None  <3/week  ≥3/week | 1.00  0.97 (0.44-2.14)  1.85 (0.87-3.95)  1.00  1.05 (0.39-2.80)  1.52 (0.52-4.44)  1.00  0.84 (0.24-3.02)  2.40 (0.79-7.36) | Age, sex, smoking |
| Key TJ, 2014, United Kingdom | EPIC-Oxford Study and Oxford Vegetarian Study | 1980-1984 - 2010  1993-1999 - 2010,  14.9 years follow-up | 61647 men and women, age 20-89 years: 124 bladder cancer cases | FFQ, 45 items (OVS)  Validated FFQ, 130 food items (EPIC-Oxford) | Vegetarian/vegans vs. meat eater  Vegetarian/vegans vs. meat eater | | 0.62 (0.38-0.99)  0.65 (0.40-1.03) +BMI | Age, sex, study/method of recruitment, smoking status and cigarettes/day, alcohol, physical activity |
| Parra-Soto S, 2022, United Kingdom | UK Biobank | 2006-2010 - 2020, 10.6 years follow-up | 409110 men and women, age 37-73 years: 2123 bladder cancer cases | Validated FFQ | Vegetarian/vegan vs. meat eater  Vegetarian/vegan vs. meat eater | | 0.89 (0.56-1.41)  0.91 (0.57-1.45) +BMI | Age, sex, deprivation, ethnicity, smoking, alcohol, total physical activity, fruits, vegetables, comorbidity |
| Fraser G et al, 2025, USA | Adventist Health Study 2 | 2002-2007 - 2010-2013, 7.91 years follow-up | 79468 men and women, age ≥30 years: 117 urothelial cancer cases | Validated FFQ, >200 food items | All vegetarians vs. meat eaters  All vegetarians vs. meat eaters  Vegetarian vs. meat eater  Vegetarian vs. meat eater  Vegan vs. meat eater  Vegan vs. meat eater | | 0.86 (0.59-1.27)  0.86 (0.58-1.27) +BMI  0.85 (0.55-1.32)  0.84 (0.54-1.31) +BMI  1.04 (0.54-2.00)  1.02 (0.52-1.99) +BMI | Age, sex, race, education, cigarette smoking (status and cessation) |

Abbreviations: +BMI, also adjusted for body mass index; FFQ, food frequency questionnaire.

Supplementary table 15. Cohort studies on vegetarian and vegan diets and brain cancer

| Author, publication year, country/region | Study name | Period of recruitment, year of censoring, duration of follow-up | Number of participants, sex, age: number of cases | Dietary assessment method | Comparison | | RR (95% CI) | Adjustment for confounding factors |
| --- | --- | --- | --- | --- | --- | --- | --- | --- |
| Mills PK et al, 1989, USA | Adventist Health Study | 1976 - 1982, 6 years follow-up | 34000 men and women, age ≥25 years: 21 glioma cases  10 meningioma cases | FFQ, 51 food items | Meat, poultry, fish, glioma  Meat, poultry, fish, meningioma | None  <3/week  ≥3/week  None  <3/week  ≥3/week | 1.00  1.56 (0.52-5.63)  1.75 (0.34-8.54)  1.00  0.36 (0.09-1.46)  - | Age, sex, smoking |
| Key TJ, 2014, United Kingdom | EPIC-Oxford Study and Oxford Vegetarian Study | 1980-1984 - 2010  1993-1999 - 2010,  14.9 years follow-up | 61647 men and women, age 20-89 years: 105 brain cancer cases | FFQ, 45 items (OVS)  Validated FFQ, 130 food items (EPIC-Oxford) | Vegetarian/vegan vs. meat eater  Vegetarian/vegan vs. meat eater | | 1.29 (0.78-2.13)  1.31 (0.79-2.17) +BMI | Age, sex, study/method of recruitment, smoking status and cigarettes/day, alcohol, physical activity |
| Parra-Soto S, 2022, United Kingdom | UK Biobank | 2006-2010 - 2020, 10.6 years follow-up | 409110 men and women, age 37-73 years: 790 brain cancer cases | Validated FFQ | Vegetarian/vegan vs. meat eater  Vegetarian/vegan vs. meat eater | | 0.73 (0.36-1.48)  0.73 (0.36-1.47) +BMI | Age, sex, deprivation, ethnicity, smoking, alcohol, total physical activity, fruits, vegetables, comorbidity |
| Fraser G et al, 2025, USA | Adventist Health Study 2 | 2002-2007 - 2010-2013, 7.91 years follow-up | 79468 men and women, age ≥30 years: 70 central nervous system cancer cases | Validated FFQ, >200 food items | All vegetarians vs. meat eaters  All vegetarians vs. meat eaters  Vegetarian vs. meat eater  Vegetarian vs. meat eater  Vegan vs. meat eater  Vegan vs. meat eater | | 1.07 (0.64-1.80)  1.02 (0.61-1.69) +BMI  1.22 (0.71-2.09)  1.25 (0.72-2.17) +BMI  -  - | Age, sex, race, education |

Abbreviations: +BMI, also adjusted for body mass index; FFQ, food frequency questionnaire.

Supplementary table 16. Cohort studies on vegetarian and vegan diets and non-Hodgkin's lymphoma

| Author, publication year, country/region | Study name | Period of recruitment, year of censoring, duration of follow-up | Number of participants, sex, age: number of cases | Dietary assessment method | Comparison | RR (95% CI) | Adjustment for confounding factors |
| --- | --- | --- | --- | --- | --- | --- | --- |
| Key TJ, 2014, United Kingdom | EPIC-Oxford Study and Oxford Vegetarian Study | 1980-1984 - 2010  1993-1999 - 2010,  14.9 years follow-up | 61647 men and women, age 20-89 years: 194 non-Hodgkin's lymphoma cases | FFQ, 45 items (OVS)  Validated FFQ, 130 food items (EPIC-Oxford) | Vegetarian/vegans vs. meat eater  Vegetarian/vegans vs. meat eater | 0.71 (0.48-1.05)  0.70 (0.47-1.04) +BMI | Age, sex, study/method of recruitment, smoking status and cigarettes/day, alcohol, physical activity |
| Parra-Soto S, 2022, United Kingdom | UK Biobank | 2006-2010 - 2020, 10.6 years follow-up | 409110 men and women, age 37-73 years: 1811 non-Hodgkin's lymphoma cases | Validated FFQ | Vegetarian/vegan vs. meat eater  Vegetarian/vegan vs. meat eater | 0.96 (0.72-1.28)  0.89 (0.58-1.38) +BMI | Age, sex, deprivation, ethnicity, smoking, alcohol, total physical activity, fruits, vegetables, comorbidity |
| Fraser G et al, 2025, USA | Adventist Health Study 2 | 2002-2007 - 2010-2013, 7.91 years follow-up | 79468 men and women, age ≥30 years: 271 lymphoma cases | Validated FFQ, >200 food items | All vegetarians vs. meat eaters  All vegetarians vs. meat eaters  Vegetarian vs. meat eater, age 65  Vegetarian vs. meat eater, age 65  Vegetarian vs. meat eater, age 85  Vegetarian vs. meat eater, age 85  Vegan vs. meat eater, age 65  Vegan vs. meat eater, age 65  Vegan vs. meat eater, age 85  Vegan vs. meat eater, age 85 | 0.75 (0.59-0.97)  0.79 (0.61-1.03) +BMI  0.64 (0.46-0.91)  0.67 (0.47-0.95) +BMI  0.61 (0.40-0.95)  0.63 (0.41-0.98) +BMI  0.90 (0.56-1.46)  0.97 (0.60-1.59) +BMI  0.44 (0.20-0.98)  0.47 (0.21-1.05) +BMI | Age, sex, race, education, cigarette smoking (status and cessation) |

Abbreviations: +BMI, also adjusted for body mass index; FFQ, food frequency questionnaire.

Supplementary table 17. Cohort studies on vegetarian and vegan diets and multiple myeloma

| Author, publication year, country/region | Study name | Period of recruitment, year of censoring, duration of follow-up | Number of participants, sex, age: number of cases | Dietary assessment method | Comparison | RR (95% CI) | Adjustment for confounding factors |
| --- | --- | --- | --- | --- | --- | --- | --- |
| Key TJ, 2014, United Kingdom | EPIC-Oxford Study and Oxford Vegetarian Study | 1980-1984 - 2010  1993-1999 - 2010,  14.9 years follow-up | 61647 men and women, age 20-89 years: 65 multiple myeloma cases | FFQ, 45 items (OVS)  Validated FFQ, 130 food items (EPIC-Oxford) | Vegetarian/vegans vs. meat eater  Vegetarian/vegans vs. meat eater | 0.23 (0.09-0.59)  0.23 (0.09-0.60) +BMI | Age, sex, study/method of recruitment, smoking status and cigarettes/day, alcohol, physical activity |
| Parra-Soto S, 2022, United Kingdom | UK Biobank | 2006-2010 - 2020, 10.6 years follow-up | 409110 men and women, age 37-73 years: 966 multiple myeloma cases | Validated FFQ | Vegetarian/vegan vs. meat eater  Vegetarian/vegan vs. meat eater | 0.96 (0.55-1.67)  0.99 (0.57-1.72) +BMI | Age, sex, deprivation, ethnicity, smoking, alcohol, total physical activity, fruits, vegetables, comorbidity |
| Fraser G et al, 2025, USA | Adventist Health Study 2 | 2002-2007 - 2010-2013, 7.91 years follow-up | 79468 men and women, age ≥30 years: 99 myeloma cases | Validated FFQ, >200 food items | All vegetarians vs. meat eaters  All vegetarians vs. meat eaters  Vegetarian vs. meat eater  Vegetarian vs. meat eater  Vegan vs. meat eater  Vegan vs. meat eater | 1.42 (0.93-2.18)  1.36 (0.88-2.10) +BMI  1.35 (0.82-2.21)  1.29 (0.78-2.12) +BMI  1.25 (0.58-2.69)  1.14 (0.52-2.49) +BMI | Age, sex, race, education |

Abbreviations: +BMI, also adjusted for body mass index; FFQ, food frequency questionnaire.

Supplementary table 18. Cohort studies on vegetarian and vegan diets and leukemia

| Author, publication year, country/region | Study name | Period of recruitment, year of censoring, duration of follow-up | Number of participants, sex, age: number of cases | Dietary assessment method | Comparison | RR (95% CI) | Adjustment for confounding factors |
| --- | --- | --- | --- | --- | --- | --- | --- |
| Key TJ, 2014, United Kingdom | EPIC-Oxford Study and Oxford Vegetarian Study | 1980-1984 - 2010  1993-1999 - 2010,  14.9 years follow-up | 61647 men and women, age 20-89 years: 128 leukemia cases | FFQ, 45 items (OVS)  Validated FFQ, 130 food items (EPIC-Oxford) | Vegetarian/vegan vs. meat eater  Vegetarian/vegan vs. meat eater | 0.87 (0.55-1.39)  0.85 (0.53-1.36) +BMI | Age, sex, study/method of recruitment, smoking status and cigarettes/day, alcohol, physical activity |
| Parra-Soto S, 2022, United Kingdom | UK Biobank | 2006-2010 - 2020, 10.6 years follow-up | 409110 men and women, age 37-73 years: 1168 leukemia cases | Validated FFQ | Vegetarian/vegan vs. meat eater  Vegetarian/vegan vs. meat eater | 1.11 (0.67-1.82)  1.14 (0.69-1.87) +BMI | Age, sex, deprivation, ethnicity, smoking, alcohol, total physical activity, fruits, vegetables, comorbidity |
| Fraser G et al, 2025, USA | Adventist Health Study 2 | 2002-2007 - 2010-2013, 7.91 years follow-up | 79468 men and women, age ≥30 years: 78 lymphoid leukemia cases  68 myeloid leukemia cases | Validated FFQ, >200 food items | All vegetarians vs. meat eaters, lymphoid leukemia  All vegetarians vs. meat eaters  Vegetarian vs. meat eater  Vegetarian vs. meat eater  Vegan vs. meat eater  Vegan vs. meat eater  All vegetarians vs. meat eaters, myeloid leukemia  All vegetarians vs. meat eaters  Vegetarian vs. meat eater  Vegetarian vs. meat eater  Vegan vs. meat eater  Vegan vs. meat eater | 0.77 (0.48-1.25)  0.80 (0.49-1.30) +BMI  0.72 (0.41-1.24)  0.72 (0.41-1.26) +BMI  -  -  1.21 (0.71-2.04)  1.12 (0.67-1.88) +BMI  1.20 (0.68-2.11)  1.27 (0.72-2.25) +BMI  0.97 (0.37-2.52)  1.07 (0.40-2.86) +BMI | Age, sex, race, education |

Abbreviations: +BMI, also adjusted for body mass index; FFQ, food frequency questionnaire.

Supplementary Table 19. Study quality assessment using modified Newcastle-Ottawa scale

| Author, publication year, country/ region | | Selection | | | Comparability | Outcome assessment | | | Total |
| --- | --- | --- | --- | --- | --- | --- | --- | --- | --- |
|  | Cancer site | Selection of non-exposed cohort | Exposure ascertainment | Demonstration of outcome not present at start | 0.25 points for each adjustment | Outcome assessment | Long enough follow-up (≥3 years) | Adequacy of follow-up (<10% lost) |  |
| Mills PK et al, 1989, USA | Breast | 1 | 1 | 1 | 1.75 | 1 | 1 | 1 | 7.75 |
| Mills PK et al, 1989, USA | Prostate | 1 | 1 | 1 | 0.5 | 1 | 1 | 1 | 6.5 |
| Mills PK et al, 1989, USA | Brain | 1 | 1 | 1 | 0.75 | 1 | 1 | 1 | 6.75 |
| Mills PK et al, 1991, USA | Bladder | 1 | 1 | 1 | 0.75 | 1 | 1 | 1 | 6.75 |
| Fraser G et al, 1991, USA | Lung | 1 | 1 | 1 | 0.75 | 1 | 1 | 1 | 6.75 |
| Singh PN et al, 1998, USA | Colon | 1 | 1 | 1 | 1.75 | 1 | 1 | 1 | 7.75 |
| Fraser GE, 1999, USA | Uterine | 1 | 1 | 1 | 0.25 | 1 | 1 | 1 | 6.25 |
| Kiani F et al, 2006, USA | Ovarian | 1 | 1 | 1 | 1.25 | 1 | 1 | 1 | 7.25 |
| Cade JE et al, 2010, United Kingdom | Breast | 1 | 1 | 1 | 2 | 1 | 1 | 0 | 7 |
| Key TJ, 2014, United Kingdom | Multiple | 1 | 0.5 | 1 | 1.75 | 1 | 1 | 1 | 7.25 |
|  | Multiple, women-only cancers | 1 | 0.5 | 1 | 2 | 1 | 1 | 1 | 7.5 |
| Gilsing AMJ et al, 2015, Netherlands | Colorectal | 1 | 1 | 1 | 1.75 | 1 | 1 | 1 | 7.75 |
| Gilsing AMJ et al, 2016, Netherlands | Lung | 1 | 1 | 1 | 2 | 1 | 1 | 1 | 8 |
|  | Breast | 1 | 1 | 1 | 2 | 1 | 1 | 1 | 8 |
|  | Prostate | 1 | 1 | 1 | 1.75 | 1 | 1 | 1 | 7.75 |
| de Jauregui DRF et al, 2018, United Kingdom | Colorectal | 1 | 1 | 1 | 1.5 | 1 | 1 | 0 | 6.5 |
| Watling C et al, 2021, United Kingdom | Multiple | 1 | 1 | 1 | 2 | 1 | 1 | 1 | 8 |
| Parra-Soto S, 2022, United Kingdom | Multiple | 1 | 1 | 1 | 2 | 1 | 1 | 1 | 8 |
| Shyam S et al, 2018, United Kingdom | Pancreas | 1 | 1 | 1 | 1 | 1 | 1 | 0 | 6 |
| Fraser G et al, 2025, USA | Total | 1 | 1 | 1 | 2 | 1 | 1 | 0 | 7 |
|  | UADT | 1 | 1 | 1 | 1.5 | 1 | 1 | 0 | 6.5 |
|  | Stomach | 1 | 1 | 1 | 1.5 | 1 | 1 | 0 | 6.5 |
|  | Colorectal | 1 | 1 | 1 | 2 | 1 | 1 | 0 | 7 |
|  | Pancreas | 1 | 1 | 1 | 1.75 | 1 | 1 | 0 | 6.75 |
|  | Lung | 1 | 1 | 1 | 1.75 | 1 | 1 | 0 | 6.75 |
|  | Melanoma | 1 | 1 | 1 | 1.25 | 1 | 1 | 0 | 6.25 |
|  | Breast | 1 | 1 | 1 | 2 | 1 | 1 | 0 | 7 |
|  | Endometrial | 1 | 1 | 1 | 1.25 | 1 | 1 | 0 | 6.25 |
|  | Ovarian | 1 | 1 | 1 | 1.75 | 1 | 1 | 0 | 6.75 |
|  | Prostate | 1 | 1 | 1 | 1 | 1 | 1 | 0 | 6 |
|  | Renal | 1 | 1 | 1 | 1.75 | 1 | 1 | 0 | 6.75 |
|  | Urothelial | 1 | 1 | 1 | 1.5 | 1 | 1 | 0 | 6.5 |
|  | CNS | 1 | 1 | 1 | 1 | 1 | 1 | 0 | 6 |
|  | Lymphoma | 1 | 1 | 1 | 1.5 | 1 | 1 | 0 | 6.5 |
|  | Myeloma | 1 | 1 | 1 | 1 | 1 | 1 | 0 | 6 |
|  | Leukemia | 1 | 1 | 1 | 1 | 1 | 1 | 0 | 6 |

UADT; upper aerogestive tract (mouth, pharynx, larynx), CNS; central nervous system. For adjustment for confounding factors we treated smoking status and cigarettes/day or time since cessation as different variables, thus adjustment for smoking status gave 0.25 points, and additional adjustment for cigarettes/day gave another 0.25 points. No points were given for adjustment for potential mediators (e.g. BMI, diabetes or food groups). For Key et al, 2014 one point was given for loss to follow-up <10% based on personal knowledge of one of the authors (JGS) who had previously worked on analyses in this cohort.

For the articles by Gilsing et al, 2016 and by Fraser et al, 2025, multiple entries were made because analyses of different cancer types were adjusted for different confounders and therefore scored differently.

Supplementary Table 20. E-values for the associations between vegetarian and vegan diets and cancer.

| Vegetarians vs. non-vegetarians | | |
| --- | --- | --- |
| Cancer site | RR (95% CI) | E-value (lower CI) |
| Total cancer | 0.87 (0.84-0.91) | 1.56 (1.43) |
| Upper aerodigestive tract cancer | 0.97 (0.73-1.29) | 1.21 (1.00) |
| Stomach cancer | 0.55 (0.36-0.86) | 3.04 (1.60) |
| Colorectal cancer | 0.86 (0.76-0.97) | 1.60 (1.21) |
| Colon cancer | 0.79 (0.67-0.93) | 1.85 (1.36) |
| Proximal colon cancer | 0.55 (0.31-0.97) | 3.04 (1.21) |
| Distal colon cancer | 0.84 (0.57-1.22) | 1.67 (1.00) |
| Rectal cancer | 0.88 (0.70-1.11) | 1.53 (1.00) |
| Pancreatic cancer | 0.77 (0.62-0.95) | 1.92 (1.29) |
| Lung | 0.85 (0.70-1.04) | 1.63 (1.00) |
| Melanoma | 0.79 (0.66-0.94) | 1.85 (1.32) |
| Breast cancer | 0.92 (0.86-0.99) | 1.39 (1.11) |
| Breast cancer, premenopausal | 0.97 (0.79-1.19) | 1.21 (1.00) |
| Breast cancer, postmenopausal | 0.81 (0.69-0.95) | 1.77 (1.29) |
| Endometrial cancer | 0.91 (0.77-1.07) | 1.43 (1.00) |
| Ovarian cancer | 0.83 (0.68-1.02) | 1.70 (1.00) |
| Prostate cancer | 0.87 (0.57-1.00) | 1.56 (1.00) |
| Kidney cancer | 0.85 (0.64-1.11) | 1.63 (1.00) |
| Bladder cancer | 0.78 (0.62-0.98) | 1.88 (1.16) |
| Brain cancer | 0.99 (0.73-1.34) | 1.11 (1.00) |
| Non-Hodgkin's lymphoma | 0.76 (0.63-0.93) | 1.96 (1.36) |
| Multiple myeloma | 0.75 (0.32-1.77) | 2.00 (1.00) |
| Leukemia | 0.95 (0.74-1.21) | 1.29 (1.00) |
| Vegans vs. non-vegetarians | | |
| Total cancer | 0.77 (0.70-0.85) | 1.92 (1.63) |
| Colorectal cancer | 1.02 (0.71-1.48) | 1.16 (1.00) |
| Breast cancer | 0.80 (0.64-1.00) | 1.81 (1.00) |
| Breast cancer^1^ | 0.78 (0.64-0.94) | 1.88 (1.32) |
| Prostate cancer | 0.87 (0.50-1.49) | 1.56 (1.00) |
| Prostate cancer^1^ | 0.58 (0.43-0.78) | 2.84 (1.88) |

^1^Using alternative estimates at younger ages from Adventist Health Study-2.

Supplementary Table 21. Subgroup analyses of vegetarian diets and colorectal, colon and rectal cancer

|  | | Colorectal cancer | | | | | | Colon cancer | | | | | | Rectal cancer | | | | | |  |
| --- | --- | --- | --- | --- | --- | --- | --- | --- | --- | --- | --- | --- | --- | --- | --- | --- | --- | --- | --- | --- |
|  | | n | RR (95% CI) | | I^2^ | P_h_^1^ | P_h_^2^ | n | RR (95% CI) | I^2^ | P_h_^1^ | P_h_^2^ | | n | | RR (95% CI) | I^2^ | P_h_^1^ | P_h_^2^ |  |
| All studies | | 5 | 0.86 (0.76-0.97) | | 14.2 | 0.32 |  | 6 | 0.79 (0.67-0.93) | 37.7 | 0.16 |  | | 5 | | 0.88 (0.70-1.11) | 18.8 | 0.30 |  |  |
| Duration of follow-up | |  |  | |  |  |  |  |  |  |  |  | |  | |  |  |  |  |  |
| <10 years follow-up | | 1 | 0.79 (0.66-0.95) | |  |  | 0.51 | 2 | 0.71 (0.54-0.94) | 52.7 | 0.15 | 0.42 | | 1 | | 0.80 (0.53-1.20) |  |  | 0.75 |  |
| ≥10 years follow-up | | 4 | 0.89 (0.77-1.03) | | 12.9 | 0.33 |  | 4 | 0.84 (0.67-1.06) | 34.5 | 0.21 |  |  | 4 | | 0.88 (0.64-1.21) | 34.1 | 0.21 |  |  |
| Gender | |  |  | |  |  |  |  |  |  |  |  | |  | |  |  |  |  |  |
| Men | | 1 | 0.57 (0.36-0.91) | |  |  | 0.29/ 0.34^3^ | 0 |  |  |  | 0.69/ NC | | 0 | |  |  |  | 0.91/ NC |  |
| Women | | 2 | 0.89 (0.71-1.11) | | 0 | 0.39 |  | 1 | 0.71 (0.47-1.08) |  |  |  |  | 1 | | 0.91 (0.55-1.52) |  |  |  |  |
| Men and women | | 3 | 0.90 (0.74-1.08) | | 45.1 | 0.16 |  | 5 | 0.80 (0.67-0.97) | 45.3 | 0.12 |  |  | 4 | | 0.86 (0.64-1.16) | 35.7 | 0.20 |  |  |
| Geographic location | |  |  | |  |  |  |  |  |  |  |  | |  | |  |  |  |  |  |
| Europe | | 4 | 0.89 (0.77-1.03) | | 12.9 | 0.33 | 0.51 | 4 | 0.84 (0.67-1.06) | 34.5 | 0.21 | 0.42 | | 4 | | 0.88 (0.64-1.21) | 34.1 | 0.21 | 0.75 |  |
| America | | 1 | 0.79 (0.66-0.95) | |  |  |  | 2 | 0.71 (0.54-0.94) | 52.7 | 0.15 |  |  | 1 | | 0.80 (0.53-1.20) |  |  |  |  |
| Population | |  |  | |  |  |  |  |  |  |  |  | |  | |  |  |  |  |  |
| General population | | 4 | 0.89 (0.77-1.03) | | 12.9 | 0.33 | 0.51 | 4 | 0.84 (0.67-1.06) | 34.5 | 0.21 | 0.42 | | 4 | | 0.88 (0.64-1.21) | 34.1 | 0.21 | 0.75 |  |
| Adventist population | | 1 | 0.79 (0.66-0.95) | |  |  |  | 2 | 0.71 (0.54-0.94) | 52.7 | 0.15 |  |  | 1 | | 0.80 (0.53-1.20) |  |  |  |  |
| Number of cases | |  |  | |  |  |  |  |  |  |  |  | |  | |  |  |  |  |  |
| Cases <500 | | 2 | 0.83 (0.64-1.08) | | 0 | 0.70 | 0.81 | 5 | 0.81 (0.67-0.98) | 42.4 | 0.14 | 0.47 | | 4 | | 0.93 (0.72-1.21) | 16.7 | 0.31 | 0.38 |  |
| Cases 500-<1000 | | 2 | 0.90 (0.69-1.17) | | 72.5 | 0.06 |  | 0 |  |  |  |  |  | 0 | |  |  |  |  |  |
| Cases ≥1000 | | 1 | 0.78 (0.61-1.01) | |  |  |  | 1 | 0.66 (0.46-0.95) |  |  |  |  | 1 | | 0.70 (0.44-1.11) |  |  |  |  |
| Study quality | |  |  | |  |  |  |  |  |  |  |  | |  | |  |  |  |  |  |
| 0-3 stars | | 0 |  | |  |  | NC | 0 |  |  |  | NC | | 0 | |  |  |  | NC |  |
| >3-6 | | 0 |  | |  |  |  | 0 |  |  |  |  |  | 0 | |  |  |  |  |  |
| >6-8 | | 5 | 0.86 (0.76-0.97) | | 14.2 | 0.32 |  | 6 | 0.79 (0.67-0.93) | 37.7 | 0.16 |  |  | 5 | | 0.88 (0.70-1.11) | 18.8 | 0.30 |  |  |
| Adjustments | | | | | | | | | | | | | | | | | | | |  |
| Age | Yes | 5 | | 0.86 (0.76-0.97) | 14.2 | 0.32 | NC | 6 | 0.79 (0.67-0.93) | 37.7 | 0.16 | NC | 5 | | 0.88 (0.70-1.11) | | 18.8 | 0.30 | NC | |
|  | No | 0 | |  |  |  |  | 0 |  |  |  |  | 0 | |  | |  |  |  |  |
| Education | Yes | 2 | | 0.81 (0.65-1.00) | 0 | 0.61 | 0.67 | 2 | 0.81 (0.51-1.29) | 57.5 | 0.13 | 0.89 | 2 | | 0.60 (0.28-1.30) | | 18.3 | 0.27 | 0.24 |  |
|  | No | 3 | | 0.87 (0.73-1.05) | 49.9 | 0.14 |  | 4 | 0.78 (0.64-0.95) | 47.1 | 0.13 |  | 3 | | 0.96 (0.77-1.21) | | 0 | 0.47 |  |  |
| Socioeconomic status | Yes | 2 | | 0.79 (0.65-0.96) | 0 | 0.90 | 0.46 | 2 | 0.68 (0.52-0.90) | 0 | 0.80 | 0.39 | 2 | | 0.79 (0.56-1.11) | | 0 | 0.45 | 0.55 |  |
|  | No | 3 | | 0.90 (0.74-1.08) | 45.1 | 0.16 |  | 4 | 0.83 (0.66-1.03) | 53.9 | 0.09 |  | 3 | | 0.90 (0.61-1.34) | | 44.0 | 0.17 |  |  |
| Smoking | Yes | 4 | | 0.89 (0.77-1.03) | 12.9 | 0.33 | 0.51 | 5 | 0.78 (0.62-0.98) | 50.1 | 0.09 | 0.92 | 4 | | 0.88 (0.64-1.21) | | 34.1 | 0.21 | 0.75 |  |
|  | No | 1 | | 0.79 (0.66-0.95) |  |  |  | 1 | 0.80 (0.65-0.98) |  |  |  | 1 | | 0.80 (0.53-1.20) | |  |  |  |  |
| Alcohol | Yes | 4 | | 0.87 (0.57-1.00) | 32.8 | 0.22 | 0.73 | 5 | 0.80 (0.66-0.97) | 48.2 | 0.10 | 0.71 | 4 | | 0.85 (0.62-1.16) | | 39.0 | 0.18 | 0.88 |  |
|  | No | 1 | | 0.80 (0.58-1.11) |  |  |  | 1 | 0.71 (0.47-1.08) |  |  |  | 1 | | 0.91 (0.55-1.52) | |  |  |  |  |
| Physical activity | Yes | 5 | | 0.86 (0.76-0.97) | 14.2 | 0.32 | NC | 6 | 0.79 (0.67-0.93) | 37.7 | 0.16 | NC | | 5 | 0.88 (0.70-1.11) | | 18.8 | 0.30 | NC |  |
|  | No | 0 | |  |  |  |  | 0 |  |  |  |  |  | 0 |  | |  |  |  |  |

N denotes the number of risk estimates

^1^ P for heterogeneity within each subgroup,

^2^ P for heterogeneity between subgroups with meta-regression analysis,

^3^ P for heterogeneity between men and women (studies with men and women combined were excluded)

^4^ Analysis restricted to studies that reported both BMI-adjusted and BMI-unadjusted estimates

NC = not calculable

Supplementary Table 22. Subgroup analyses of vegetarian diets and lung, breast and prostate cancer

|  | | Lung cancer | | | | | | Breast cancer | | | | | Prostate cancer | | | | | |  |
| --- | --- | --- | --- | --- | --- | --- | --- | --- | --- | --- | --- | --- | --- | --- | --- | --- | --- | --- | --- |
|  | | n | RR (95% CI) | | I^2^ | P_h_^1^ | P_h_^2^ | n | RR (95% CI) | I^2^ | P_h_^1^ | P_h_^2^ | n | | RR (95% CI) | I^2^ | P_h_^1^ | P_h_^2^ |  |
| All studies | | 5 | 0.85 (0.70-1.04) | | 19.6 | 0.29 |  | 6 | 0.92 (0.86-0.99) | 0 | 0.45 |  | 5 | | 0.87 (0.75-1.00) | 43.1 | 0.14 |  |  |
| Duration of follow-up | |  |  | |  |  |  |  |  |  |  |  |  | |  |  |  |  |  |
| <10 years follow-up | | 2 | 0.74 (0.58-0.96) | | 0 | 0.56 | 0.34 | 3 | 0.90 (0.77-1.05) | 43.0 | 0.17 | 0.97 | 2 | | 0.95 (0.85-1.06) | 0 | 0.33 | 0.32 |  |
| ≥10 years follow-up | | 3 | 0.96 (0.73-1.26) | | 18.7 | 0.29 |  | 3 | 0.92 (0.83-1.02) | 0 | 0.54 |  | 3 | | 0.81 (0.65-1.00) | 36.0 | 0.21 |  |  |
| Gender | |  |  | |  |  |  |  |  |  |  |  |  | |  |  |  |  |  |
| Men | | 0 |  | |  |  | NC/ NC^3^ | - |  |  |  | - | 5 | | 0.87 (0.75-1.00) | 43.1 | 0.14 | - |  |
| Women | | 0 |  | |  |  |  | 6 | 0.90 (0.82-0.97) | 16.2 | 0.31 |  | - | |  |  |  |  |  |
| Men and women | | 5 | 0.85 (0.70-1.04) | | 19.6 | 0.29 |  | - |  |  |  |  | - | |  |  |  |  |  |
| Geographic location | |  |  | |  |  |  |  |  |  |  |  |  | |  |  |  |  |  |
| Europe | | 3 | 0.96 (0.73-1.26) | | 18.7 | 0.29 | 0.30 | 4 | 0.91 (0.83-1.00) | 0 | 0.72 | 0.76 | 3 | | 0.81 (0.65-1.00) | 36.0 | 0.21 | 0.32 |  |
| America | | 2 | 0.74 (0.58-0.96) | | 0 | 0.56 |  | 2 | 0.89 (0.69-1.15) | 69.7 | 0.07 |  | 2 | | 0.95 (0.85-1.06) | 0 | 0.33 |  |  |
| Population | |  |  | |  |  |  |  |  |  |  |  |  | |  |  |  |  |  |
| General population | | 3 | 0.96 (0.73-1.26) | | 18.7 | 0.29 | 0.30 | 4 | 0.91 (0.83-1.00) | 0 | 0.72 | 0.76 | 3 | | 0.81 (0.65-1.00) | 36.0 | 0.21 | 0.32 |  |
| Adventist population | | 2 | 0.74 (0.58-0.96) | | 0 | 0.56 |  | 2 | 0.89 (0.69-1.15) | 69.7 | 0.07 |  | 2 | | 0.95 (0.85-1.06) | 0 | 0.33 |  |  |
| Number of cases | |  |  | |  |  |  |  |  |  |  |  |  | |  |  |  |  |  |
| Cases <250 | | 2 | 0.90 (0.50-1.63) | | 66.0 | 0.09 | 0.41 | 2 | 0.75 (0.60-0.94) | 0 | 0.77 | 0.11 | 1 | | 0.82 (0.60-1.12) |  |  | 0.99 |  |
| Cases 250-<500 | | 2 | 0.78 (0.60-1.01) | | 0 | 0.79 |  | 2 | 0.88 (0.69-1.11) |  |  |  | 2 | | 0.89 (0.71-1.10) | 0 | 0.34 |  |  |
| Cases ≥500 | | 1 | 0.78 (0.53-1.14) | |  |  |  | 3 | 0.95 (0.88-1.03) | 0 | 0.77 |  | 2 | | 0.83 (0.60-1.16) | 82.6 | 0.02 |  |  |
| Study quality | |  |  | |  |  |  |  |  |  |  |  |  | |  |  |  |  |  |
| 0-3 stars | | 0 |  | |  |  | NC | 0 |  |  |  | NC | 0 | |  |  |  | 0.15 |  |
| >3-6 | | 0 |  | |  |  |  | 0 |  |  |  |  | 1 | | 0.97 (0.86-1.10) |  |  |  |  |
| >6-8 | | 5 | 0.85 (0.70-1.04) | | 19.6 | 0.29 |  | 6 | 0.92 (0.86-0.99) | 0 | 0.45 |  | 4 | | 0.80 (0.69-0.93) | 4.8 | 0.37 |  |  |
| Adjustments | | | | | | | | | | | | | | | | | | |  |
| Age | Yes | 5 | | 0.85 (0.70-1.04) | 19.6 | 0.29 | NC | 6 | 0.92 (0.86-0.99) | 0 | 0.45 | NC | 5 | 0.87 (0.75-1.00) | | 43.1 | 0.14 | NC | |
|  | No | 0 | |  |  |  |  | 0 |  |  |  |  | 0 |  | |  |  |  |  |
| Education | Yes | 2 | | 0.80 (0.56-1.12) | 0 | 0.82 | 0.78 | 5 | 0.91 (0.82-1.00) | 15.6 | 0.32 | 0.82 | 4 | 0.87 (0.72-1.05) | | 55.4 | 0.08 | 0.89 |  |
|  | No | 3 | | 0.86 (0.62-1.21) | 57.4 | 0.10 |  | 1 | 0.93 (0.82-1.07) |  |  |  | 1 | 0.84 (0.66-1.07) | |  |  |  |  |
| Socioeconomic status | Yes | 1 | | 0.78 (0.53-1.14) |  |  | 0.75 | 2 | 0.91 (0.79-1.05) | 0 | 0.71 | 0.89 | 1 | 0.69 (0.54-0.89) | |  |  | 0.12 |  |
|  | No | 4 | | 0.87 (0.66-1.14) | 36.2 | 0.20 |  | 4 | 0.91 (0.80-1.02) | 34.8 | 0.20 |  | 4 | 0.93 (0.84-1.03) | | 0 | 0.54 |  |  |
| Smoking | Yes | 4 | | 0.88 (0.71-1.09) | 24.0 | 0.27 | 0.42 | 4 | 0.91 (0.83-1.00) | 0 | 0.72 | 0.76 | 3 | 0.81 (0.65-1.00) | | 36.0 | 0.21 | 0.32 |  |
|  | No | 1 | | 0.63 (0.34-1.16) |  |  |  | 2 | 0.89 (0.69-1.15) | 69.7 | 0.07 |  | 2 | 0.95 (0.85-1.06) | | 0 | 0.33 |  |  |
| Alcohol | Yes | 3 | | 0.96 (0.73-1.26) | 18.7 | 0.29 | 0.30 | 4 | 0.91 (0.83-1.00) | 0 | 0.72 | 0.76 | 3 | 0.81 (0.65-1.00) | | 36.0 | 0.21 | 0.32 |  |
|  | No | 2 | | 0.74 (0.58-0.96) | 0 | 0.56 |  | 2 | 0.89 (0.69-1.15) | 69.7 | 0.07 |  | 2 | 0.95 (0.85-1.06) | | 0 | 0.33 |  |  |
| Physical activity | Yes | 4 | | 0.88 (0.71-1.09) | 24.0 | 0.27 | 0.42 | 5 | 0.94 (0.87-1.01) | 0 | 0.67 | 0.19 | 3 | 0.81 (0.65-1.00) | | 36.0 | 0.21 | 0.32 |  |
|  | No | 1 | | 0.63 (0.34-1.16) |  |  |  | 1 | 0.76 (0.59-0.98) |  |  |  | 2 | 0.95 (0.85-1.06) | | 0 | 0.33 |  |  |
| Age at menarche | Yes | - | |  |  |  |  | 4 | 0.88 (0.76-1.02) | 36.4 | 0.19 | 0.72 | - |  | |  |  |  |  |
|  | No | - | |  |  |  |  | 2 | 0.93 (0.84-1.03) | 0 | 0.99 |  | - |  | |  |  |  |  |
| Hormone replacement therapy | Yes | - | |  |  |  |  | 3 | 0.94 (0.82-1.07) | 12.5 | 0.32 | 0.58 | - |  | |  |  |  |  |
|  | No | - | |  |  |  |  | 3 | 0.90 (0.82-1.00) | 3.6 | 0.35 |  | - |  | |  |  |  |  |
| Oral contraceptive use | Yes | - | |  |  |  |  | 3 | 0.94 (0.82-1.07) | 12.5 | 0.32 | 0.58 | - |  | |  |  |  |  |
|  | No | - | |  |  |  |  | 3 | 0.90 (0.82-1.00) | 3.6 | 0.35 |  | - |  | |  |  |  |  |

N denotes the number of risk estimates

^1^ P for heterogeneity within each subgroup,

^2^ P for heterogeneity between subgroups with meta-regression analysis,

^3^ P for heterogeneity between men and women (studies with men and women combined were excluded)

^4^ Analysis restricted to studies with both BMI-adjusted and BMI-unadjusted estimates.

NC = not calculable

Cut-off for number of cases for breast cancer is <500, 500-<1000, and ≥1000

Supplemental Table 23. World Cancer Research Fund grading criteria

| Grading | Criteria |
| --- | --- |
| Convincing | A convincing relationship should be robust enough to be highly unlikely to be modified in the foreseeable future as new evidence accumulates. All of the following are generally required:  - Evidence from more than one study type  - Evidence from at least two independent cohort studies  - No substantial unexplained heterogeneity within or between study types or in different populations relating to the presence or absence of an association, or direction of effect  - Good quality studies to exclude with confidence the possibility that the observed association results from random or systematic error, including confounding, measurement error, and selection bias  - Presence of a plausible biological gradient in the association. Such a gradient need not be linear or even in the same direction across different levels of exposure, so long as this can be explained plausibly  - Strong and plausible experimental evidence, either from human studies or relevant animal models, that typical human exposures can lead to relevant outcomes |
| Probable | All of the following are generally required:  - Evidence from at least two independent cohort studies, or at least five case-control studies  - No substantial unexplained heterogeneity within or between study types or in different populations relating to the presence or absence of an association, or direction of effect  - Good quality studies to exclude with confidence the possibility that the observed association results from random or systematic error, including confounding, measurement error, and selection bias  - Evidence for biological plausibility |
| Limited - suggestive | All of the following are generally required:  - Evidence from at least two independent cohort studies, or at least five case-control studies  - The direction of effect is generally consistent though some unexplained heterogeneity may be present  - Evidence for biological plausibility |
| Limited - no conclusion | Evidence is so limited that no firm conclusion can be made, but this does not mean that there is evidence of no relationship. The evidence might be graded "limited - no conclusion" for several reasons:  - limited number of studies  - inconsistency of direction of effect  - poor quality of studies (e.g. lack of adjustment for known confounders)  - or any combination of these factors |
| Substantial effect on risk unlikely | All of the following are generally required:  - Evidence from more than one study type  - Evidence from at least two independent cohort studies  - Summary estimate of effect close to 1.0 for comparison of high versus low exposure categories  - No substantial unexplained heterogeneity within or between study types or in different populations  - Good quality studies to exclude with confidence the possibility that the absence of association results from random or systematic error, including inadequate power, imprecision or error in exposure measurement, inadequate range of exposure, confounding, and selection bias  - Absence of a demonstrable biological gradient (dose response)  - Absence of strong and plausible experimental evidence, either from human studies or relevant animal models, that typical human exposures lead to relevant outcomes |

Specific upgrading factors:

1) Presence of a plausible biological gradient (dose response) in the association. Such a gradient need not be linear or even in the same direction across the different levels of exposure, so long as this can be explained plausibly.

2) A particularly large summary effect size (an odds ratio or relative risk of 2.0 or more, depending on the unit of exposure) after appropriate control for confounders.

3) Evidence from randomised trials in humans.

4) Evidence from appropriately controlled experiments demonstrating one or more plausible and specific mechanisms actually operating in humans.

5) Robust and reproducible evidence from experimental studies in appropriate animal models showing that typical human exposures can lead to relevant health outcomes.

Supplemental Table 24. Evidence grading for vegetarian diets and cancer risk

|  | Reduced risk | Increased risk |
| --- | --- | --- |
| Convincing | - | - |
| Probable | Total, colorectal, colon, breast, postmenopausal breast | - |
| Limited-suggestive | Stomach, proximal colon, pancreas, melanoma, bladder, non-Hodgkin's lymphoma | - |
| Limited - no conclusion | Upper aerodigestive tract, distal colon, rectal, lung, premenopausal breast, endometrial, ovarian, prostate, kidney, brain, multiple myeloma, leukemia | |

Supplemental Table 25. Evidence grading for vegan diets and cancer risk

|  | Reduced risk | Increased risk |
| --- | --- | --- |
| Convincing | - | - |
| Probable | - | - |
| Limited-suggestive | Total, breast | - |
| Limited - no conclusion | Colorectal, prostate | |

Supplementary Table 26. Justification for evidence grading for vegetarian diets and cancer risk

| Requirements for grading of convincing | Total cancer | Colorectal cancer | Colon cancer | Breast cancer | Postmenopausal breast cancer |
| --- | --- | --- | --- | --- | --- |
| Statistically significant and robust association | Statistically significant inverse association | Statistically significant inverse association | Statistically significant inverse association | Statistically significant inverse association | Statistically significant inverse association |
| Evidence from at least two independent cohort studies | 3 cohort studies included | 6 cohort studies (5 risk estimates) | 7 cohort studies (6 risk estimates) | 7 cohort studies (6 risk estimates) | 4 cohort studies (3 risk estimates) |
| No substantial unexplained heterogeneity within or between study types or in different populations relating to the presence or absence of an association, or direction of effect | There is no heterogeneity (I^2^=0%) | Low heterogeneity (I^2^=14.2%) | No heterogeneity (I^2^=0%) | Low heterogeneity (I^2^=16.2%) | No heterogeneity (I^2^=0%) |
| Good quality studies to exclude with confidence the possibility that the observed association results from random or systematic error, including confounding, measurement error, and selection bias | Too few studies to assess publication bias and to do subgroup analyses.  Studies have adjusted for most important confounders.  All studies have used a baseline assessment of diet, which likely may have attenuated the observed associations.  All studies excluded prevalent cancer cases at baseline. Exposed and non-exposed participants were selected from the same populations. | No indication of publication bias.  Most studies have adjusted for important confounders. Little heterogeneity in subgroup analyses by study characteristics and adjustments, though estimates are not completely robust to the influence of individual studies.  All studies have used a baseline assessment of diet, which likely may have attenuated the observed associations. | No indication of publication bias.  Most studies have adjusted for important confounders. Little heterogeneity in subgroup analyses by study characteristics and adjustments, and estimates are not completely robust to the influence of individual studies.  All studies have used a baseline assessment of diet, which likely may have attenuated the observed associations. | Publication bias (Egger's test, p=0.04), however, this is driven by one study, which when excluded attenuated Egger's test to p=0.22, however, the inverse association remained (RR=0.90, 0.81-0.99).  Most studies have adjusted for important confounders and there is little evidence of heterogeneity by adjustment for confounders in subgroup analyses. Estimates are largely robust to the influence of individual studies.  All studies have used a baseline assessment of diet, which likely may have attenuated the observed associations. | Too few studies to assess publication bias and to do subgroup analyses.  Studies have adjusted for the most important confounders.  All studies have used a baseline assessment of diet, which likely may have attenuated the observed associations.  All studies excluded prevalent cancer cases at baseline. Exposed and non-exposed participants were selected from the same populations. |
| Presence of a plausible biological gradient in the association. Such a gradient need not be linear or even in the same direction across different levels of exposure, so long as this can be explained plausibly | Cannot be assessed as the exposure is dichotomous. | Cannot be assessed as the exposure is dichotomous. | Cannot be assessed as the exposure is dichotomous. | Cannot be assessed as the exposure is dichotomous. | Cannot be assessed as the exposure is dichotomous. |
| Strong and plausible experimental evidence, either from human studies or relevant animal models, that typical human exposures can lead to relevant outcomes | - Reduced adiposity and insulin resistance  - Reduced inflammation  - Improvements in microbiota  - Avoidance of meat-related carcinogens (heme iron, heterocyclic amines, polycyclic aromatic hydrocarbons)  - Increased intake of food components with established benefits - fruits, vegetables, whole grains, fibre | - Reduced adiposity and insulin resistance  - Reduced inflammation  - Improvements in microbiota. Production of short-chain fatty acids (e.g. butyrate) from fermentation of fibre, which have benefits on the colorectum.  - Avoidance of meat-related carcinogens (heme iron, heterocyclic amines, polycyclic aromatic hydrocarbons)  - Increased intake of food components with established benefits - fruits, vegetables, whole grains, fibre | - Reduced adiposity and insulin resistance  - Reduced inflammation  - Improvements in microbiota. Production of short-chain fatty acids (e.g. butyrate) from fermentation of fibre, which have benefits on the colon.  - Avoidance of meat-related carcinogens (heme iron, heterocyclic amines, polycyclic aromatic hydrocarbons)  - Increased intake of food components with established benefits - fruits, vegetables, whole grains, fibre | - Reduced adiposity and insulin resistance  - Reduced estrogen and increased sex-hormone binding globulin levels  - Reduced inflammation  - Improvements in microbiota.  - Avoidance of meat-related carcinogens (heme iron, heterocyclic amines, polycyclic aromatic hydrocarbons)  - Increased intake of food components with benefits - fruits, vegetables, fibre, soy | - Reduced adiposity and insulin resistance  - Reduced estrogen and increased sex-hormone binding globulin levels  - Reduced inflammation  - Improvements in microbiota.  - Avoidance of meat-related carcinogens (heme iron, heterocyclic amines, polycyclic aromatic hydrocarbons)  - Increased intake of food components with benefits - fruits, vegetables, fibre, soy |
| Final grading and justification for overall assessment. | Probable evidence that vegetarian diets reduce total cancer risk.  Justification: Primarily based on highly significant main analysis, with no heterogeneity. Biologically plausible mechanisms exist. Results are also consistent with evidence on food groups and cancer risk. | Probable evidence that vegetarian diets reduce colorectal cancer risk.  Justification: Primarily based on highly significant main analysis, with no heterogeneity. Biologically plausible mechanisms exist. Results are also consistent with evidence on food groups and cancer risk. | Probable evidence that vegetarian diets reduce colon cancer risk.  Justification: Primarily based on highly significant main analysis, with no heterogeneity. Biologically plausible mechanisms exist. Results are also consistent with evidence on food groups and cancer risk. | Probable evidence that vegetarian diets reduce breast cancer risk.  Justification: Primarily based on significant main analysis, with no heterogeneity. Biologically plausible mechanisms exist. Results are also consistent with evidence on food groups and cancer risk. | Probable evidence that vegetarian diets reduce postemenopausal breast cancer risk.  Justification: Primarily based on highly significant main analysis with no heterogeneity. Biologically plausible mechanisms exist. Results are also consistent with evidence on food groups and cancer risk. |

Please see the discussion of the main article for relevant references regarding underlying mechanisms.

Supplementary Figure 1. Funnel plot for the association between vegetarian diets and colorectal cancer

Supplementary Figure 2. Funnel plot for the association between vegetarian diets and colon cancer

Supplementary Figure 3. Funnel plot for the association between vegetarian diets and rectal cancer

Supplementary Figure 4. Funnel plot for the association between vegetarian diets and lung cancer

Supplementary Figure 5. Funnel plot for the association between vegetarian diets and breast cancer

Supplementary Figure 6. Funnel plot for the association between vegetarian diets and prostate cancer

Supplementary Figure 7. Influence analysis of vegetarian diets and colorectal cancer

------------------------------------------------------------------------------

Study omitted | e^coef. [95% Conf. Interval]

-------------------+----------------------------------------------------------

Fraser, 2025 | 0.89191478 0.76908475 1.034362

Watling, 2022 | 0.87704051 0.75769478 1.0151845

de Jauregui, 2018 | 0.86582571 0.74734628 1.0030881

Gilsing, 2015 | 0.8532111 0.73928249 0.98469687

Key, 2014 | 0.79711008 0.70102918 0.90635955

-------------------+----------------------------------------------------------

Combined | 0.85726103 0.76024001 0.96666377

------------------------------------------------------------------------------

Supplementary Figure 8. Influence analysis of vegetarian diets and colon cancer

------------------------------------------------------------------------------

Study omitted | e^coef. [95% Conf. Interval]

-------------------+----------------------------------------------------------

Fraser, 2025 | 0.77950972 0.62000537 0.9800486

Parra-Soto, 2022 | 0.80910283 0.67142123 0.97501743

de Jauregui, 2018 | 0.79657519 0.65672821 0.96620184

Gilsing, 2015 | 0.76246738 0.64157629 0.90613765

Key, 2014 | 0.7420013 0.63355166 0.86901498

Singh, 1998 | 0.83023244 0.71196544 0.96814513

-------------------+----------------------------------------------------------

Combined | 0.78618799 0.66538632 0.92892133

------------------------------------------------------------------------------

Supplementary Figure 9. Influence analysis of vegetarian diets and rectal cancer

------------------------------------------------------------------------------

Study omitted | e^coef. [95% Conf. Interval]

-------------------+----------------------------------------------------------

Fraser, 2025 | 0.88440931 0.64413345 1.2143133

Parra-Soto, 2022 | 0.93106353 0.71926564 1.2052284

de Jauregui, 2018 | 0.8499583 0.62331307 1.1590148

Gilsing, 2015 | 0.90684301 0.7409901 1.109818

Key, 2014 | 0.77608573 0.59834808 1.0066199

-------------------+----------------------------------------------------------

Combined | 0.87762278 0.69529187 1.1077675

------------------------------------------------------------------------------

Supplementary Figure 10. Influence analysis of vegetarian diets and lung cancer

------------------------------------------------------------------------------

Study omitted | e^coef. [95% Conf. Interval]

-------------------+----------------------------------------------------------

Fraser, 2025 | 0.89024508 0.6769641 1.1707212

Parra-Soto, 2022 | 0.86899799 0.66401118 1.1372663

Gilsing, 2016 | 0.84799182 0.66698492 1.0781206

Key, 2014 | 0.76187706 0.62168115 0.93368876

Fraser, 1991 | 0.88100642 0.7097441 1.0935946

-------------------+----------------------------------------------------------

Combined | 0.85237903 0.69639277 1.0433049

------------------------------------------------------------------------------

Supplementary Figure 11. Influence analysis of vegetarian diets and breast cancer

------------------------------------------------------------------------------

Study omitted | e^coef. [95% Conf. Interval]

-------------------+----------------------------------------------------------

Fraser, 2025 | 0.89214712 0.81661195 0.97466922

Parra-Soto, 2022 | 0.91183656 0.83072698 1.0008653

Gilsing, 2016 | 0.92767233 0.8614074 0.99903476

Key, 2014 | 0.90851152 0.82175028 1.004433

Cade, 2010 | 0.921538 0.84602588 1.00379

Mills, 1989 | 0.93811971 0.86900401 1.0127325

-------------------+----------------------------------------------------------

Combined | 0.92179014 0.85666624 0.99186477

------------------------------------------------------------------------------

Supplementary Figure 12. Influence analysis of vegetarian diets and prostate cancer

------------------------------------------------------------------------------

Study omitted | e^coef. [95% Conf. Interval]

-------------------+----------------------------------------------------------

Fraser, 2025 | 0.80266333 0.69148993 0.93171042

Watling, 2021 | 0.93425053 0.84442228 1.0336345

Gilsing, 2016 | 0.84543765 0.72051471 0.99201971

Key, 2014 | 0.86745197 0.71523482 1.0520643

Mills, 1989 | 0.87069619 0.72872549 1.0403256

-------------------+----------------------------------------------------------

Combined | 0.86516949 0.74784183 1.0009045

------------------------------------------------------------------------------

Supplementary Figure 13. Summary estimates from cohort studies on vegetarian diets and cancer that reported both BMI-unadjusted and BMI-adjusted results.

Supplementary Figure 14. Summary estimates from cohort studies on vegan diets and cancer that reported both BMI-unadjusted and BMI-adjusted results.
